# Influence of Polyfluorinated Side Chains and Soft‐Template Method on the Surface Morphologies and Hydrophobic Properties of Electrodeposited Films from Fluorene Bridged Dicarbazole Monomers

**DOI:** 10.1002/cphc.202200371

**Published:** 2022-10-18

**Authors:** David Possetto, Ilir Pecnikaj, Gabriela Marzari, Simonetta Orlandi, Silvia Sereno, Marco Cavazzini, Gianluca Pozzi, Fernando Fungo

**Affiliations:** ^1^ Instituto de Investigaciones en Tecnologías Energéticas y Materiales Avanzados IITEMA-UNRC-CONICET) Departamento de Química Universidad Nacional de Río Cuarto Agencia Postal 3 X5804BYA Río Cuarto Argentina; ^2^ University of Medicine Tirana Department of Pharmacy Rruga e Dibrës Nr. 371 AL1005 Tiranë Albania; ^3^ CNR Institute of Chemical Sciences and Technologies “Giulio Natta” (CNR SCITEC) UOS Golgi, via Golgi 19 20133 Milan Italy

**Keywords:** cyclic voltammetry, hydrophobic effect, nanostructures, nanotubes, electropolymerization

## Abstract

A clear case of relationship between the monomer molecular structure and the capability of tuning the morphology of electrodeposited gas bubbles template polymer thin films is shown. To this end, a series of fluorene‐bridged dicarbazole derivatives containing either linear or terminally branched polyfluorinated side chains connected to the fluorene subunit were synthesized and their electrochemical properties were investigated. The new compounds underwent electrochemical polymerization over indium tin oxide electrodes to give hydrophobic films with nanostructural and morphological properties strongly dependent on the nature of the side chains. Gas bubbles templated electropolymerization was next achieved by the addition of tiny amounts of water to the monomer solutions, without using surfactants. Within the investigated set of molecules, the nanostructural properties of the soft‐templated films obtained from monomers bearing linear side chains could be fine‐tuned by adjusting electrochemical parameters, leading to superhydrophobic surfaces.

## Graphical abstract

**Electrochemically generated gas microbubbles** have a templating action on the formation of electrodeposited polymer films from organic solutions of fluorene‐bridged dicarbazole monomers bearing polyfluorinated side chains, leading to superhydrophobic surfaces. The soft‐templated mechanism only depends on the nature of the side chains, and not on the formation of aqueous micelles.

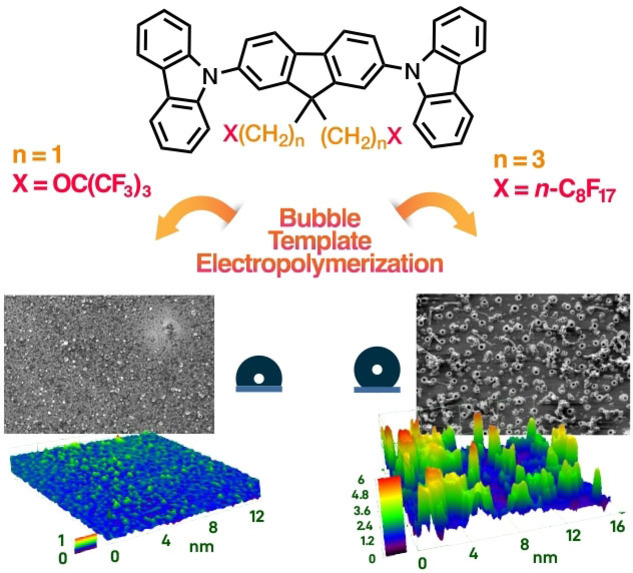

## Introduction

Conducting polymers are unique organic materials able to form solid thin films with many desirable properties, such as the ability to conduct charge, specific optical parameters, and capability to form nanostructured surfaces.^[1]^ These materials are at the core of many important applications, including electrochromic devices,^[2]^ solar cells,^[3]^ supercapacitors^[4]^ and batteries.^[5]^ In addition, rapid advances in surface science and design of materials with controlled morphology, both in shape and dimension, have pushed toward an intensive exploitation of new possibilities concerning chemical,^[6]^ physical,^[7]^ biological,^[8]^ electrochemical sensors,^[9]^ and electronic applications.^[10]^ This broad potential demands multifunctional polymers with specific physicochemical properties (light interaction, charge transport, solubility, adequate chemical stability for the device operating exigency, ability to form solid thin films, right relative position in the energy scale of the valence‐conduction bands regarding the device's contacts work function, biocompatibility, and so on), which can be customized thanks to the infinite versatility of organic chemistry.^[11]^

In this scenery, conducting polymers continuously draw significant academic and technological interest due to the combination of the electronic and optical proprieties of inorganic semiconductors with the attractive feature typically associated to conventional polymers such as mechanical flexibility.^[12]^ Moreover, countless conducting polymers materials able to form films with specific surface properties (anti‐bacterial,^[13]^ separation of oil from water,^[14]^ hydrophobic‐hydrophilic,^[15]^ anti‐icing,^[16]^ cell growth compatibility,^[17]^ among others) have been developed by manipulating the monomers structure at the molecular level or/and the film morphology at the micro‐nanoscale.^[1]^

Electrochemical polymerization is a convenient engineering route to make conducting polymers films.^[18]^ The electropolymerization reaction occurs on the electrodes surfaces and is particularly useful when thin films are desired on conducting substrates. This technique only requires an electropolymerizable monomer that is soluble in the electrolyte solution, and it allows forming films in one‐step at room temperature, with film thickness control and morphological surface tuning (e. g. smoothness, roughness and porosity).[^17^, ^19^] Indeed, the progressive growth of the polymer film and its properties can be controlled by adjustable parameters such as cell setup, electrolyte composition and variables related to the used electrochemical method (potentiodynamic polarization, galvanostatic, pulse deposition, etc.).^[20]^ High voltages or the use of catalyst/initiators for the polymerization are not necessary, so this electrochemical technique represents a convenient approach to the fabrication of unique surface structures.[^19^, ^20d^]

While much research has been performed to develop electropolymerizable monomers able to generate organic conducting polymers with different capabilities or physicochemical properties,[^17^, ^21^] building out controlled electropolymerization processes that lead to films structured at the micro‐nanoscale can further expand the potential of these materials. In this regard, electrodeposition processes with the concomitant generation of gas microbubbles at the electrode surface by electrochemically discharge of a gas precursor as water are particularly intriguing.[^20a^, ^20d^, ^22^] Indeed, the generated microbubbles work as soft template that makes that the polymer precipitate around them forming ordered pores or tubular structures. By means of the electrochemical parameters control, it is possible to obtain a precise tuning of conducting polymer structures at the micro‐nanoscale. This technique allows adjusting the surface properties for applications like wettability, among others, and it is arousing ever increasing interest.[^20d^, ^23^] So far, much attention has been paid to the optimization of the electrolyte composition in order to stabilize gas microbubbles during the polymerization processes. Several examples of this technique thus rely on the use of aqueous solvents combined with surfactants, and of monomers soluble in such a reaction environment.[^23^, ^24^] Soft‐template electropolymerization in standard organic solvents containing traces of water, which would allow the use of a wider range of non‐aqueous soluble monomers, is emerging as a promising alternative method.^[25]^ In this case, the operative mechanism through which nanostructured surfaces are formed is a matter of debate. Fradin et.al. have recently proposed that reverse aqueous micelles formed prior to the onset of polymerization, and not gas microbubbles, would act as the actual template responsible for the formation of porous nanostructures.^[25d]^ In their mechanism, the monomer and electrolyte assume the role of a surfactant in the reverse micelle system adsorbed onto the electrode; the inner water is then oxidized or reduced, forming gases that are released during the electrodeposition. In this scenery, the monomer ability to form reverse micelles would be crucial, whereas a direct relationship between its molecular structure features and the morphology of the electrodeposited polymeric films could be ruled out.[^20a^, ^25d^]

**Scheme 1:**
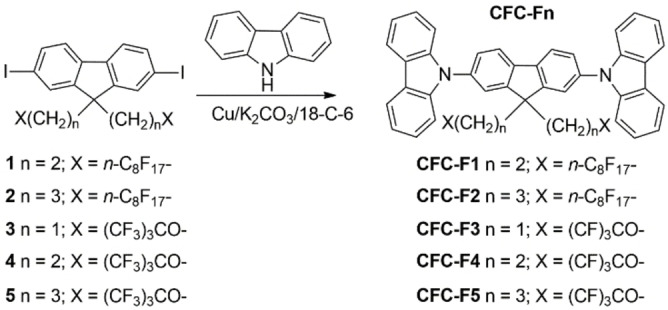
Functionalization of fluorene derivatives and molecular structure of **CFC‐Fn** monomers.

Carbazole–Fluorene–Carbazole conjugated monomers (**CFC**) are a class of interesting precursors of electrodeposited coatings films over conductive substrates, thanks to repetitive electrochemically promoted oxidative homocoupling reactions occurring at the carbazole sites (see Figure S39, Supporting Information).[^26^, ^27^] Their molecular design easily allows to bond tailored substituents to the sp^3^ carbon atom at the 9‐position of fluorene ring. We recently showed that the introduction of two pendant 1*H*,1*H*,2*H*,2*H*‐perfluorodecyl chains at 9‐position in **CFC** materials (compound **CFC‐F1**, Scheme 1) significantly facilitates the **CFC** thin‐film electrodeposition improving their surface morphology, water repellency, and electrochromic behavior regarding alkyl chains of similar lengths.^[27]^ We have now extended the **CFC** monomers range by synthesizing compounds having linear or terminally branched polyfluorinated pendant chains (**CFC‐Fn**, Scheme 1), with the aim to evaluate the effect of this structural aspect on the electrodeposition processes and film properties when using in‐situ generated microbubbles from the discharge of water traces in an organic electrolyte. Our findings show an incontestable direct relationship between the nature of the side chains of the monomer and the obtained morphology of the electrodeposited polymer thin films, which also allowed us to test the proposed crucial role of reverse micelles formation. In parallel, we reveal the potential of electrodeposited **CFC‐Fn** films as superhydrophobic coatings, deriving from the optimization of the electropolymerization process. By combining molecular structure variations and control of electrochemical parameters it turned out possible to tune the morphology of the **CFC** electrode coating and modulate through this the film surface hydrophobicity.

## Results and Discussion

### Synthesis of CFC‐Fn Monomers

The **CFC‐Fn** monomers were obtained in high yields (76–97 %) by bis *N*‐arylation of the corresponding polyfluorinated 2,7 diodofluorene intermediates **1**–**5** with carbazole under mild Ullmann‐type conditions, as previously reported in the case of **CFC‐F1** (Scheme 1).^[27]^

**Scheme 2:**
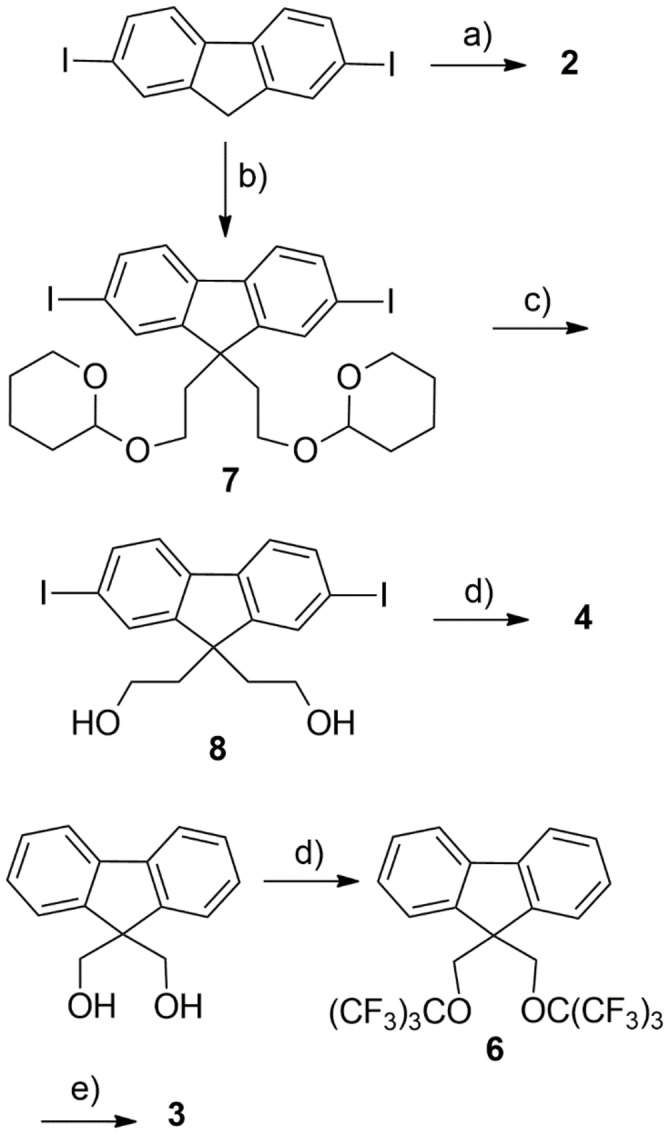
Synthesis of **CFC‐Fn** precursors. a) C_8_F_17_(CH_2_)_3_I, t‐BuOK, THF; b) 2‐(2‐Bromoethoxy)tetrahydro‐2*H*‐pyran, TBABr, NaOH_aq_, Toluene; c) HCl_aq_/EtOH; d) (CF_3_)_3_COH, DIAD, PPh_3_, THF; e) I_2_, HIO_3_, H_2_SO_4_, AcOH.

Compounds **1** and **5** were available from earlier studies,[^27^, ^28^] whereas **2**, **3** and **4** were prepared as outlined in Scheme 2. Synthesis details are reported in the Supporting Information. In brief, 2,7‐diiodofluorene was reacted with 1*H*,1*H*,2*H*,2*H*,3*H*,3*H*‐perfluoroundecyl iodide under basic conditions to give **2** in 72 % yield, whereas Mitsunobu reaction between perfluoro‐*tert*‐butanol and 9,9‐bis(hydroxymethyl)fluorene, followed by iodination of the polyfluorinated intermediate **6** afforded **3** in 60 % overall yield. Finally, compound **4** was conveniently prepared in three steps from 2,7‐diiodofluorene. Nucleophilic functionalization of the 9‐position of the latter with 2‐(2‐bromoethoxy)tetrahydro‐2*H*‐pyran afforded the protected diol **7** in 77 % yield. After acidic cleavage of tetrahydropyranyl protections, the diol **8** was reacted with perfluoro‐*tert*‐butanol under Mitsunobu conditions to give **4** in 61 % yield.

### Electrochemical Properties of CFC‐Fn Monomers

**Figure 1:**
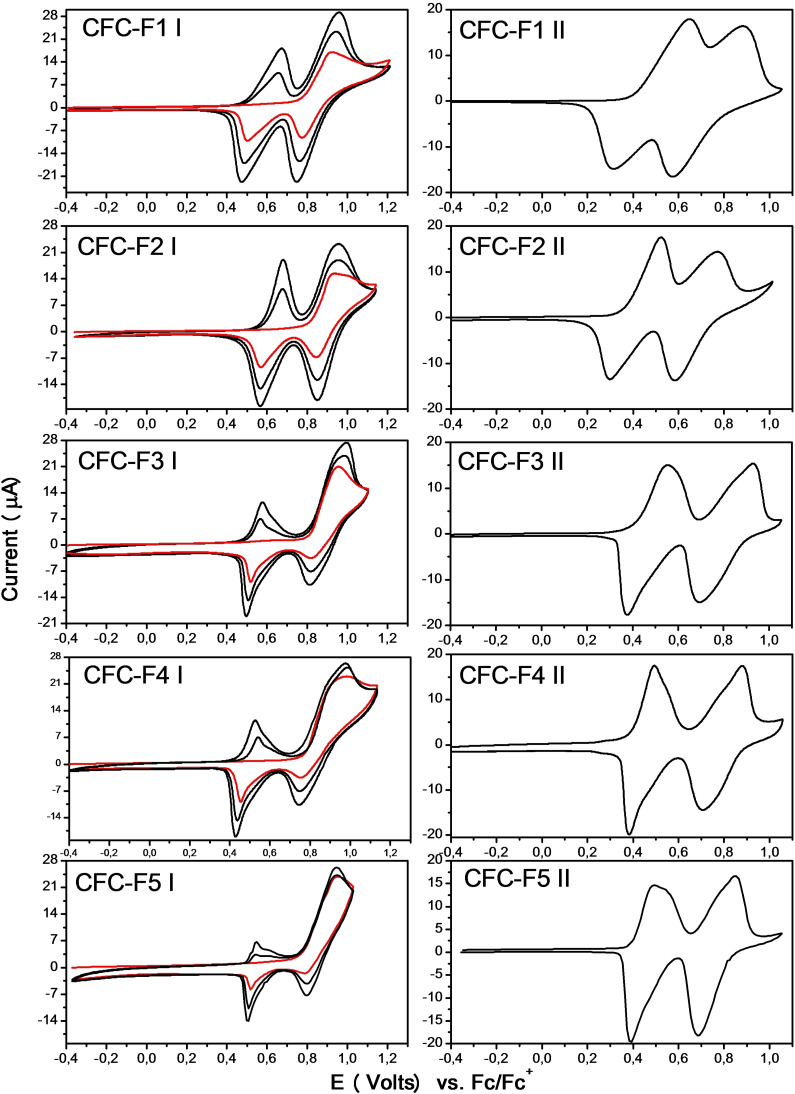
Series I: first (red) and 2nd–3rd (black) cyclic voltammograms of **CFC‐Fn**. Series II: electrochemical response of the **CFC‐Fn** electrodeposited films. Conditions: Pt working electrode, electrolyte TBAPF_6_ 0.1 M solution in DCE, v=0.1 V/s.

The electrochemical properties of the monomers and their capability to electropolymerize through the well‐known selective mechanism involving the reactive carbazole sites (Figure S39) were studied by cyclic voltammetry in anhydrous electrolyte and the correspondent results are shown in Figure 1. The **CFC‐Fn** cyclic voltammograms obtained on polished Pt electrode surface show similar features with an oxidation process in the first scan to positive potentials (red line in **CFC‐Fn** |, Figure 1) with a current peak at around 0.93 V. Then, when the potential sweep direction is inverted, two reduction waves are detected at ∼0.95 V and ∼0.6 V. During the second voltammetry cycle (see black line in **CFC‐Fn** |, Figure 1) a new oxidation process appears at 0.62 V, which is complementary to the peak at 0.54 V registered in the first potential sweep.

The **CFC‐Fn** monomer electrochemical polymerization ability was studied by applying multiple potential scans and as consequence, it was observed a comparable electropolymerization rate with similar progressive current growth for all the systems studied (see black line in **CFC‐Fn** |, Figure 1). This behavior is indicative of the formation of a conductive and electroactive film on the working electrode. In order to further support this hypothesis, the electrodes were removed from the cell and tested in a monomer‐free supporting electrolyte solution, where electrochemical signals characteristic of redox‐active modified electrodes were observed (see **CFC‐Fn** || in Figure 1). Relevant electrochemical parameters of **CFC‐Fn** monomers and corresponding deposited films are reported in Table 1. It can be appreciated from Figure 1 that the oxidation of all **CFC‐Fn** electrodeposited films produces two well defined and reversible voltammetric waves that appear in the range between 0.4 V and 1.0 V with similar film oxidation onset potential close at 0.40 V.

**Table 1:** Electrochemical parameters of CFC−Fn monomers.

	Oxidation Potentials	
Monomer	E_monomer_ [V]^[a]^	E_film_ [V]^[b]^
**CFC‐F1**	0.86	0.42
**CFC‐F2**	0.92	0.38
**CFC‐F3**	0.94	0.39
**CFC‐F4**	0.95	0.40
**CFC‐F5**	0.94	0.40

[a] Peak potential. [b] Onset potentials of the electrodeposited films determined from intersection between the baseline and the current signal. The potentials values are expressed in volt vs Fc/Fc+.^[29]^

The electrochemical behavior of **CFC** derivatives bearing either fully hydrogenated or partly fluorinated linear alkyl chains in the 9‐position has been recently established.^[27]^ The electropolymerization mechanism was found to involve the reaction between two carbazole radical cations to form dicarbazole linkages between fluorene units as polymer chain propagation path. It was also observed that the films growth rate and film surface morphology were heavily dependent on the chemical nature of the pendant chains on the fluorene bridge. Thus, the monomer **CFC‐F1** bearing pendant −(CH_2_)_2_C_8_F_17_ chains produced homogeneous and very stable film while an analogous monomer **CFC‐H** bearing pendant −(CH_2_)_9_CH_3_ chains did not. In the present case, all **CFC‐Fn** films show similar voltammogram shape and current wave potential position (see **CFC‐Fn** || in Figure 1), which indicates that nature of the fluorinated side chains has only minor effects on the redox properties.

### Soft‐Template Assisted Electropolymerization of CFC‐Fn Monomers

The influence of the **CFC‐Fn** molecular structure on the surface morphology of the electrodeposited film on ITO electrode was investigated, in connection with the use of water as soft‐template precursor, in the absence of surfactants or other gas bubbles stabilizers.

It is known that the mere presence of traces of water in the organic electrolyte solutions of apt monomers can induce the formation of nanotubular structures in the electrodeposited polymer film polymer.[^22^, ^23^] This can be attributed to the formation of templating microbubbles of O_2_ or H_2_ that are generated by the electrochemical discharge of water on the electrode surface through application of a potential where water is oxidized or reduced, respectively. However, the use of big area ITO electrodes in an organic electrolyte with water traces makes it difficult to detect the onset of those processes. Therefore, a blank experiment with the electrolyte containing 1000 ppm of water using ITO electrode was performed (see Figure S36, Supporting Information). H_2_ and O_2_ evolution onset potentials of −0.5 V and 0.86 V were determined, respectively, showing that water oxidation producing O_2_ microbubbles overlaps with the oxidation of the **CFC‐Fn** monomers (see Table 1). The templating effect of H_2_ microbubbles generated at a lower potential than **CFC‐Fn** electropolymerization onset would be in principle more conveniently exploited. Indeed, their size and density on the surface electrode could be modulated by controlling electrochemical parameters (applied potential, concentration, time, etc.) in a clear voltammogram zone.[^20d^, ^22^] Unfortunately, ITO electrodes suffer from degradation when exposed to hydrogen evolution from water discharge, which affects their chemical composition and conductivity.^[30]^ For this reason the electropolymerization of **CFC‐Fn** monomers was potentiodynamically tested by fixing their concentration (5 mM), applying one cycle potential sweep at 20 mV/s starting at −0.3 V (where the H_2_ evolution does not occur) to 1.1 V, and varying the water concentration in the range of 0 to 5000 ppm.

**Figure 2:**
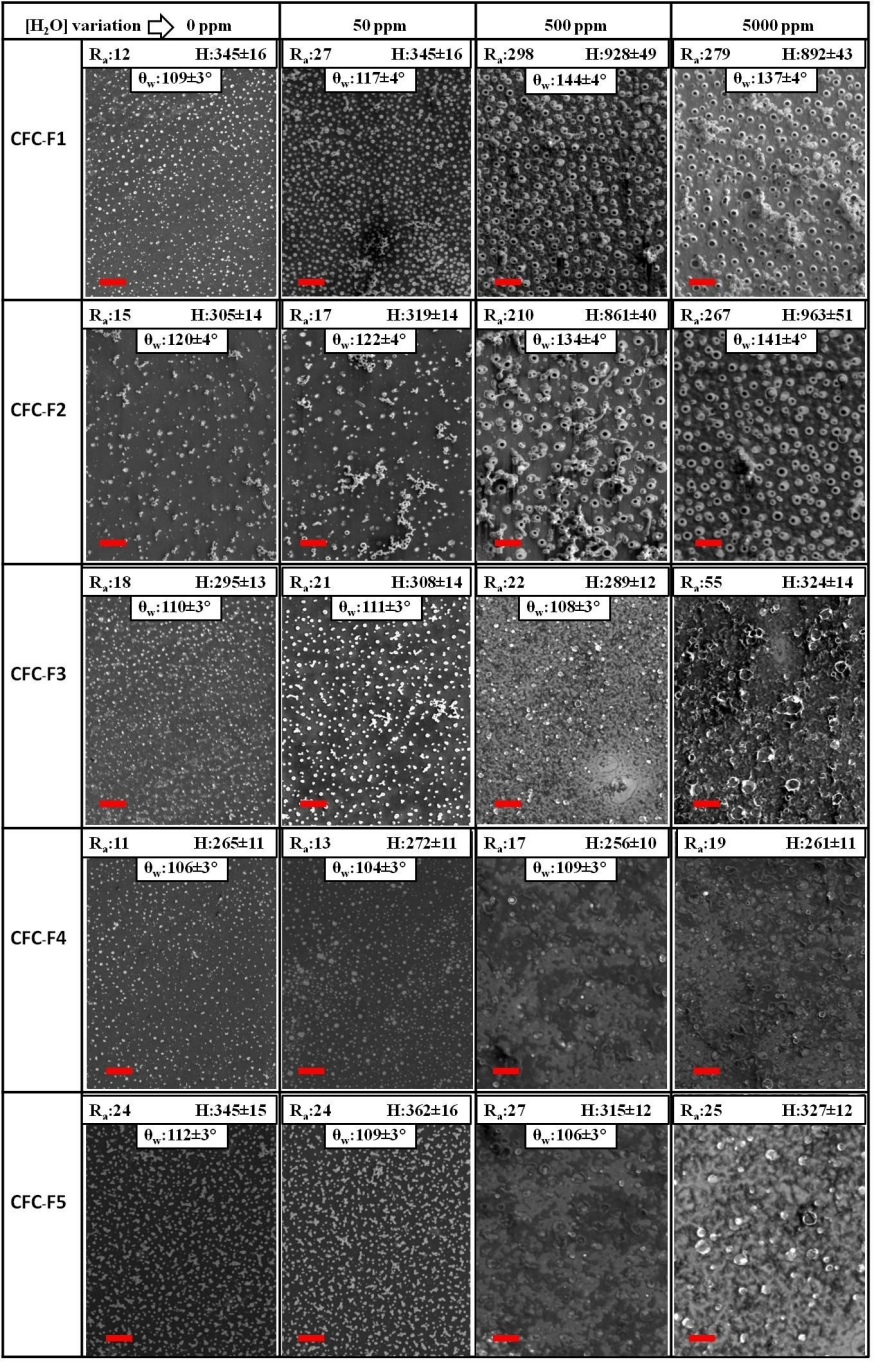
SEM. images of **CFC‐Fn** films electrodeposited by cyclic voltammetry on the surface on ITO electrodes. The monomer concentration was fixed at 5 mM in a DCE solution containing 0.1 M TBAPF_6_ and different H_2_O concentrations (rows: 0, 50, 500 and 5000 ppm) as the electrolyte. Scan rate: 20 mV/s. Insert: R_a_ (arithmetic mean roughness) and H (height) in nm, θ_w_ (apparent contact angle). Scale bar: 4 μm.

SEM images of the obtained **CFC‐Fn** films on ITO electrode are shown in Figure 2. Electrodeposition under anhydrous electrolyte conditions led in all cases to films covering the electrode surface with typical globular‐shaped surface morphology. These cauliflower like structures are formed and then expand to touch each other from nucleation sites on the electrode surface.[^20d^, ^22^] **CFC‐Fn** films grown from anhydrous electrolyte solution look similar, with a surface arithmetic mean roughness (R_a_) and film height (H) that oscillates in the range 11–24 nm and 265–345 nm, respectively (See inset in Figure 2). Just **CFC‐F4** seems to form microstructured surfaces with bigger chunk structures. On the other hand, the addition of water to the electrolyte resulted in diverse surface morphologies of the electrodeposited films, which were dependent on the nature of the side chains present in the monomer.

In particular, for both **CFC‐F1** and **CFC‐F2** monomers, which hold linear polyfluoroalkyl chains (see Scheme 1), water (500 or 5000 ppm) induced the formation of hollow or tubular structures protruding from the electrodeposited films, with an average pore external diameter of approximately 600 nm. R_a_ of **CFC‐F1** and **CFC‐F2** films surfaces varied with water concentration from 0 to 5000 ppm in the following ranges: 12–298 nm and 15–267 nm, respectively. The height of **CFC‐F1** and **CFC‐F2** films also showed important variations with water concentration, reaching a plateau at 500 ppm of water of 968 nm and 868 nm, respectively. On the contrary, the electro‐formation of hollow structures was not observed under any of the explored conditions (changes in monomers and water concentration, and scan rate) for the **CFC‐F3**, **‐F4**, and **‐F5** monomers that have branched perfluoro‐*tert*‐butoxy terminal groups in their pendant chains. In those cases, both film roughness and height were similar to those of films growth in the absence of water (See inset in Figure 2).

To date, the operative mechanism through which surface nanostructures are formed when electrodeposited polymer film are generated in organic solvents in the presence of gas microbubbles due to the oxidation or reduction of water traces remains unclear.^[25d]^ On one hand, the different **CFC‐Fn** outcomes of the water soft‐templated electropolymerization can be understood considering that, in the absence of surfactants, the molecular structure of the monomer must warranty not only the correct orientation of the electropolymerizable moieties at gas/liquid interphase, but also the stabilization of the gas microbubbles in order to allow the solid to grow around them.^[25c]^ This has been proved in the case of thiophene‐based electropolymerizable monomers, for which the introduction of rigid aromatic side substituents that are fit to this double role favors the formation of nanotubular structures, contrary to flexible alkyl side chains C_n_H_2n+1_.[^25c^, ^31^] In the same way, the presence of conformationally restricted linear *n*‐C_8_F_17_ segments in **CFC‐F1** and **CFC‐F2** might facilitate the tight molecular packing of these monomers at the gas/electrolyte interface by minimizing the associated entropic loss,^[32]^ and might promote the stabilization of the oxygen microbubbles,^[33]^ thus explaining the formation of hollow structures. On the other hand, it has been recently proposed that the formation of porous nanostructures by electropolymerization in organic solvents/electrolyte with traces of water requires the preliminary formation of reverse micelles in solution bulk before the bubble formation and the onset of polymerization.^[25d]^ The dynamic light scattering (DLS) behavior of **CFC‐Fn** DCE solutions containing TBAPF_6_ was thus evaluated to verify the existence of a relationship between reverse micelle formation and the observed films nanostructuration. To this end the monomer **CFC‐Fn** (5 mM) and electrolyte concentration (0.1 M) were fixed, while the water content was variated from 0 to 5000 ppm. Prior to the measurements the monomers solutions were three times filtered using an 0.2 μm PTFE membrane, a standard procedure to avoid artifacts from dust or particles present in the original solution. These DLS experiments failed to reveal the presence of aggregates of any kind, suggesting that, independently on the nature of the side chains, **CFC‐Fn** monomers are not able to form inverse micelles under the conditions used for the soft‐template electropolymerization experiments that lead to the surfaces depicted in Figure 2. It should be noted that, upon filtration on 0.2 μm PTFE membrane, the electropolymerization behavior of solutions containing **CFC‐Fn**/TBAPF_6_/H_2_O was in all respects identical to that of the corresponding unfiltered solutions (see Figure S42 for examples of morphology comparison). On account of this, a possible templating role for oversize reverse micelles that would have been eliminated by filtration can be safely dismissed. Taken together, these evidences stand against the hypothesis that reverse micelle formation is a general pre‐requisite for the formation of porous nanostructures by electropolymerization in organic electrolytes containing traces of water. Instead, it appears that the molecular structural characteristics of the monomers intrinsically play a major role in the film morphological tuning.

In agreement with this, the observed minor differences in pore size, height, and mostly pore density (e. g. 28,7±0,4×10^4^ tube per mm^2^ and 11,6±0,2×10^4^ tube per mm^2^ at 500 ppm of water for **CFC‐F1** and **CFC‐F2**, respectively) reflect the presence of either methylene or ethylene spacers between the rigid fluorinated segments and **CFC** core.^[34]^ Finally, the poor response of **CFC‐F3**, **‐F4**, and **‐F5** to the templating action of gas microbubbles can be justified by frustrated molecular packing following the insertion of short alkyl chains terminated by −OC(CF_3_)_3_ groups.^[35]^

Further attempts to tune the morphology of electrodeposited films of **CFC‐F1** and **CFC‐F2** using water as soft‐template precursor were next performed. In particular, we found that minimization of O_2_ evolution during the electropolymerization process by matching the upper limit of voltage sweeping with the O_2_ evolution onset potential (0.85 V) gives rise to more disperse and smaller nanotubes with respect to those observed in **CFC‐F1** and **CFC‐F2** films grown by cycling voltage between −0.3 V and 1.1. V. This is exemplified by comparison of the SEM images of **CFC‐F1** and **CFC‐F2** films grown cycling the potential between −0.3 and +0.85 V in presence of 500 ppm of water (Figure S40, Supporting Information) with the images of the corresponding films obtained at a 1.1 V upper voltage limit (Figure 2). The initial concentration of monomer is another important factor that influences the micro‐nanostructure of the soft‐templated **CFC‐Fn** electrodeposited films. Experiments run at different **CFC‐F1** and **CFC‐F2** concentrations, while keeping fixed both the water amount at 500 ppm and the scan rate at 20 mV/s, revealed that tubular nanostructures were only formed when the initial monomer concentrations were higher than 3 mM (see Figure S41, Supporting Information). Below this threshold, the electrode surface is completely covered by polymer film, with the apparition of bigger chunk structures in the case of **CFC‐F2**.

### Tuning of Film Surface Hydrophobicity

**Figure 3:**
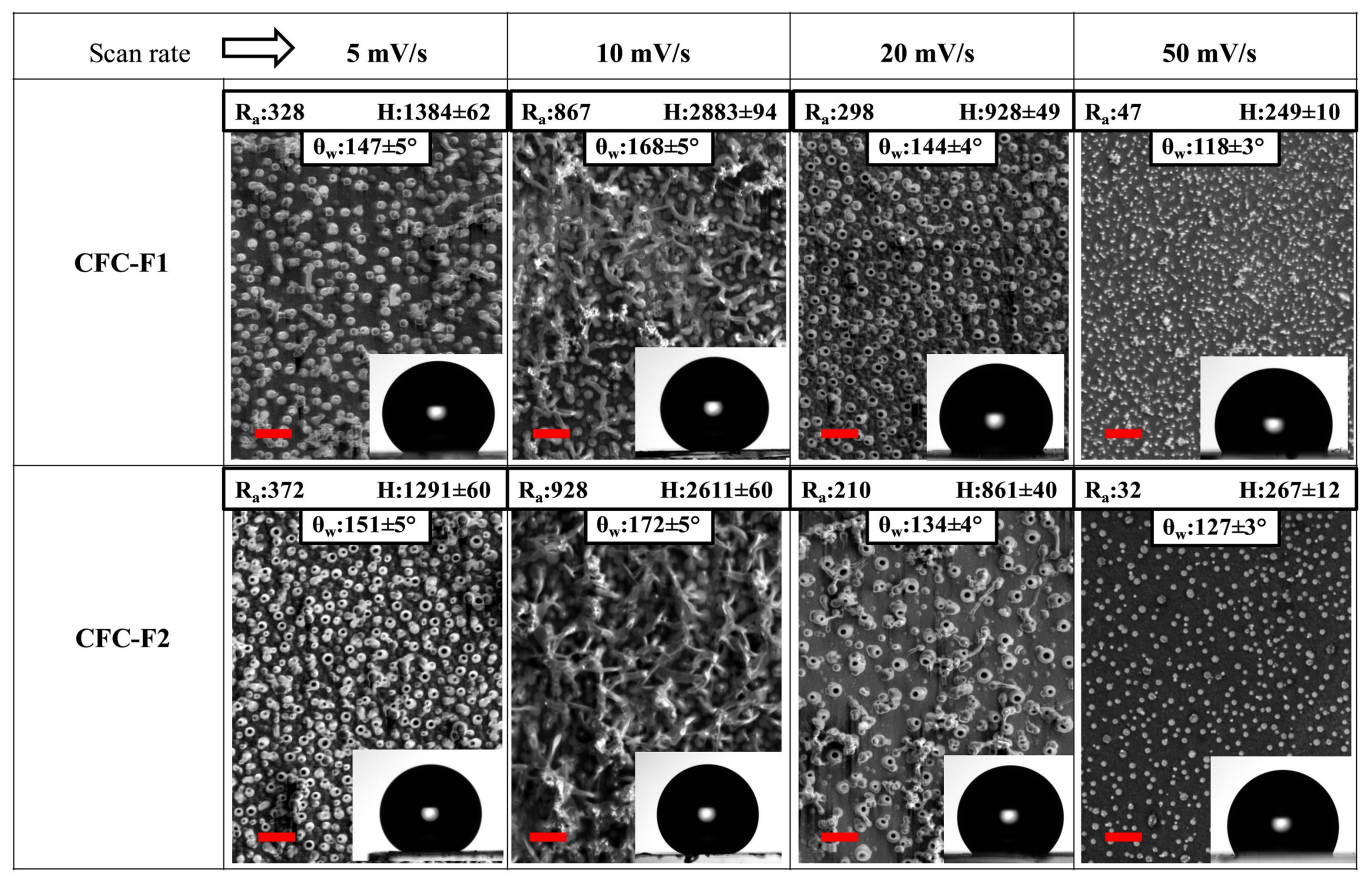
SEM images of **CFC‐F1** and **CFC‐F2** films, electrodeposited maintaining fixed monomer and water concentrations (5 mM and 500 ppm, respectively) and varying the cyclic voltammetry scan rate between 5mV/s and 50mV/s. insert: R_a_ (arithmetic mean roughness) and H (height) in nm, θ_w_ (apparent contact angle). Scale bar: 4 μm.

The surface wettability of the electrodeposited **CFC‐Fn** films was assessed by water apparent contact angle (θ_w_) measurements.^[36]^ For films grown under anhydrous conditions, the presence of polyfluoroalkyl side chains led to hydrophobic surfaces with θ_w_ values ranging between 106° (**CFC‐F4**) and 120° (**CFC‐F2**, see insets of Figure 2), with just small differences between films produced from monomers featuring −OC(CF_3_)_3_ (**CFC‐F3**, **4**, **5**) or *n*‐C_8_F_17_ (**CFC‐F1**, **2**) terminal groups in their pendant chains. On the other hand, water addition causes a gradual increase in θ_w_ value from 109° to 137°and 120° to 141° in case of the **CFC‐F1** and **CFC‐F2** films, respectively. Such a variation is not observed for monomers with terminally branched pendant chains. As discussed above, the corresponding films generated under bubble soft template conditions do not show morphological changes and this is reflected in wettability that are similar to those of films grown in the absence of water (See inset in Figure 2). We concluded that for **CFC‐F1** and **CFC‐2** the application of the previously described soft‐template method offered further possibilities for tuning the film wettability. Specifically, we focused on the surface morphology and θ_w_ values changes of the electrodeposited films induced by the stepwise increase of cyclic voltammetry scan rate from 5 mV/s to 50 mV/s while maintaining the initial concentration of monomer (5 mM) and amount of added water (500 ppm) fixed. SEM images of the **CFC‐F1** and **CFC‐F2** films thus obtained (Figure 3) show the deep effect of this single electrochemical parameter on the adjustment of the surface morphology.

Soft‐templated **CFC‐F1** and **CFC‐F2** films electrodeposited at a scan rate of 5 mV/s present uniformly dispersed nanopores with diameter of 197±20 nm; while at 10 mV/s their surface is densely packed and tangled with nanorods. At 20 mV/s, the pores are bigger (418±45 nm) and more dispersed, with polymer material filling the spaces between the pores. Finally, at 50 mV/s the electrode is completely covered by globular shape polymer films, with R_a_ values (insets in Figure 3) similar to those measured for films grown under anhydrous conditions at a scan rate of 20 mV/s (see Figure 2). R_a_ values are greatly increased for soft‐templated **CFC‐F1** and **CFC‐F2** films generated at lower scan rate. Maxima are reached at 10 mV/s for both monomers, peaking at 928 nm in the case of **CFC‐F2**. This R_a_ behavior can be associated to the three‐dimensional polymer structure growth.^[37]^ The scan rate controls the used charge in electrodeposition processes. Consequently, the height of the soft‐templated films was also tuned under electrochemical control, from 249 nm to 2883 nm, but this time at a fixed water concentration.

In the absence of templates, the electrodeposition of conducting polymers results from complex interfacial mechanisms based on the kinetics of the nucleation and growth processes, where many physical and chemical phenomena are happening at the same time. They include heterogeneous and homogenous electron transfer reactions, stability charged species, polymerization reaction and later precipitation on the electrode surface, species solubility, mass transport, charge balances, etc).^[38]^ Thus, the manipulation of the experimental parameters that affect one or more of these mechanistic steps will produce changes in the thickness, morphology, crystallinity, and other properties of the electrodeposited films. It is well known that the polymer film growth rate increases proportionally with deposition time and varies depending on the used electrochemical method. Cyclic voltammetry is a potentiodynamic technique where the variation of the potential scan rate inversely affects the growth of the polymer film. Thus, at a lower potential scan rate, the electropolymerization time and the amount of material deposited increase. In addition, as the electropolymerization time increases, the deposited polymer starts to agglomerate by growth on the previously formed material, augmenting the thickness and affecting the morphology of the film. Things get even more entangled in the presence of template gas bubbles electrochemically generated *in situ* at the same potential that induces the electropolymerization.^[20d]^ Now, all the electrochemical parameters that affect the electropolymerization process also influence bubbles formation. The number of formed bubbles and their growth are also dependent on the potential scan rate. Therefore, it is expected that a fixed scan rate will produce a unique polymer film pattern that arises from the complex relationship between the rate of polymer electrodeposition and the bubbles size, and bubbles density and growth rate on the electrode surface in this condition. Disentangling the relative contributions of the many interconnected physicochemical processes that can be affected by the cyclic voltammetry scan rate parameter and that are merged in the bubble formation and electropolymerization is beyond the scope of the present work.

On the other hand, the obtained changes in surface morphology had clear effects on the surface wettability of the soft‐templated **CFC‐F1** and **CFC‐F2** films. As shown in the insets of Figure 3, the lowest θ_w_ values, close to those obtained for the corresponding non templated films in Figure 2, were observed for films grown at a scan rate of 50 mV/s. At the opposite extreme, films with superhydrophobic properties, with θ_w_ of 168° and 172° for **CFC‐F1** and **CFC‐F2**, respectively, were obtained using a scan rate of 10 mV/s. In addition, the sliding angle (α) is another important criterion for superhydrophobic surfaces. For a 10 μL water droplet on the polymer surface resultant from scan the potential rate between 5 and 20 mV/s, sliding angles were found that oscillated 2° and 7°, for **CFC‐F1** and **CFC‐F2**, respectively. These low α values show that water droplets can roll off the surface easily, a characteristic of a low adhesion superhydrophobic polymeric surface.

The observed θ_w_ values trend follows the **CFC‐Fn** film surface roughness, in agreement with the Cassie‐Baxter model, which describes superhydrophobic phenomenon having in account the presence of air trapped between the water droplet and porous substrate.^[39]^ The electrochemical manipulation of the polymer film surface at the micro‐nano scale thus allows increasing the water contact angle by approximately 20° regarding the smooth films obtained without a soft template.

## Conclusions

We have developed a family of easy‐made fluorene‐bridged dicarbazole derivatives **CFC‐Fn** that are useful precursors of electrodeposited thin films with tunable micro‐nanostructure. For the compounds here investigated the outcome of the electropolymerization process strongly depended on the structural characteristics (size, shape, presence of an additional oxygen atom) of the polyfluorinated side chains connected to the fluorene subunit. Further control could be achieved by setting operational parameters. In particular, the templating action of gas microbubbles generated by regulated electrochemical oxidation of water was demonstrated, which does not necessarily require the prior formation of reverse aqueous micelles as recently proposed. Under these soft‐template conditions, only the **CFC‐Fn** monomers bearing linear polyfluoroalkyl pendant chains were able to form porous structures at the micro‐nanoscale. The films′ morphologies were characterized by SEM and 3D laser techniques and the obtained results clearly show the contrasting behavior of such **CFC‐Fn** monomers and those ones featuring short alkyl chains terminated by −OC(CF_3_)_3_ groups. Thus, through molecular design and fine electrochemical control, it was possible to tune the wettability of the soft‐templated film leading to superhydrophobic surfaces with a water contact angle higher than 170°. Research efforts are now directed to engineer other monomers that ease the manipulation of the film morphology by specific interactions with the oxygen or hydrogen microbubbles.

## Experimental section

### Electrochemical Polymer Films Formation and Surface Characterization

All experiments were performed at room temperature using DCE (Sintorgan), which was purified by distillation and stored over molecular sieves (Biopack, 3 Å) and CaCO_3_ (Riedel‐de Haen, 95 %), as the solvent, and 0.1 M tetra‐*n*‐butylammonium hexafluorophosphate (TBAPF_6_) (Merck) as the supporting electrolyte. The N‐substituted carbazoles electropolymerization was induced by voltamperometric technique in DCE solution with 0.1 M TBAPF_6_ as supporting electrolyte in a three‐electrode cell using a CH Instrument 700 E potentiostat. The used working electrode was indium‐tin oxide (ITO) electrodes (Biotain Crystal Co., Ltd.). The ITO electrodes were cleaned following the next protocol: first, they were washed with water and neutral detergent using a brush and then ultrasonicated in distilled water, ethanol and isopropyl alcohol. Silver wire and a loop of Pt wire were used as the quasi‐reference and the counter electrode, respectively. The silver quasi‐reference electrode was calibrated using ferrocenium/ferrocene couple (Fc^+^/Fc=0.4 V vs. saturated calomel electrode).^[29]^

The film morphology was observed with a Scanning Electron Microscope (SEM), Carl Zeiss EVO MA 10, operating at 3 KV and the electrode was examined bare, without prior metal covering.

Contact angle measurements were performed using an optical contact angle meter (Attension Theta optical tensiometer) at room temperature. A Milli‐Q water droplet (droplet size: 5 μL) was placed with a Hamilton syringe (25 μL) on the surface of the electrodeposited films. The films surfaces characterization and contact angles reported values are representative of at least three different electrodes and three consecutive measurements of water droplets. The film roughness, height and porous diameter measurements were made with 3D laser measuring microscope, LEXT OLS4000 by Olympus. The operation of LEXT OLS4000 in the confocal mode allows to obtain a spatial mapping of the examined object's surface uses a 408 nm laser diode and has a resolution on the z‐axis of 10 nm with finely controlled movement of the nosepiece. Image processing and analysis (measuring of geometrical parameters, detection of edges and surface roughness measurements) was carried out using the dedicated OLS4000 2.1 software, provided by the microscope producer.

The reverse micelle formation was explored by dynamic light scattering (DLS) measurements using a Malvern 4700 with a goniometer and an argon‐ion laser operating at 488 nm. Prior to the DSL measurements, the DSL cuvette was washed with ethanol and water, and then it was rinsed out with acetone and dried with a nitrogen stream. Also, the monomers solutions were three times filtered using an Acro disc with 0.2 μm PTFE membrane (Sigma) to avoid dust or particles present in the original solution. Before data acquisition, samples were equilibrated in the DLS instrument for 10 min at 25 °C.

## Conflict of interest

The authors declare no conflict of interest.

1

## Supporting information

As a service to our authors and readers, this journal provides supporting information supplied by the authors. Such materials are peer reviewed and may be re‐organized for online delivery, but are not copy‐edited or typeset. Technical support issues arising from supporting information (other than missing files) should be addressed to the authors.

Supporting Information

## Data Availability

Data available from the authors upon reasonable request
