# Supplementary material for: Influence of Polyfluorinated Side Chains and Soft‐Template Method on the Surface Morphologies and Hydrophobic Properties of Electrodeposited Films from Fluorene Bridged Dicarbazole Monomers
Source: Chemphyschem. 2022 Oct 18;24(2):e202200371. doi: 10.1002/cphc.202200371 (PMC10091753; doi:10.1002/cphc.202200371)
Supplement: Supplementary file 1 — Supporting Information [file CPHC-24-0-s001.pdf]

ChemPhysChem

Supporting Information

Influence of Polyfluorinated Side Chains and Soft-Template Method on the Surface Morphologies and Hydrophobic Properties of Electrodeposited Films from Fluorene Bridged Dicarbazole Monomers

David Possetto, Ilir Pecnikaj, Gabriela Marzari, Simonetta Orlandi, Silvia Sereno,
Marco Cavazzini, Gianluca Pozzi,* and Fernando Fungo*

Synthesis procedures

General remarks. All available reagents were purchased from commercial sources and were used without any further purification. Solvents were purified by standard methods and dried if necessary. Reactions were monitored by thin layer chromatography (TLC) that was conducted on plates precoated with silica gel Si 60-F254 (Merck, Germany). Column chromatography was carried out on silica gel SI 60 (Merck, Germany), mesh size 0.063 – 0.200 mm (gravimetric) or 0.040 – 0.063 mm (flash). ^{1}H NMR, ^{13}C NMR, and ^{19}F NMR spectra were recorded on Bruker AC 300 and Bruker Avance 400 spectrometers. Elemental analyses were carried out by the Departmental Service of Microanalysis (University of Milano).

*9,9-Bis(3,3,4,4,5,5,6,6,7,7,8,8,9,9,10,10,11,1,11-heptadecafluorundecyl)-2,7-diiodo-9*H*-fluorene* (**2**). In a flame-dried round-bottom flask 2,7-diiodo-9*H*-fluorene (0.63 g, 1.50 mmol) was dissolved in dry THF (3 mL) under nitrogen atmosphere. The solution was cooled to 0 °C and a solution of *t*-BuOK (0.18 g, 1.60 mmol) in dry THF (3 mL) was added dropwise under stirring. The mixture was allowed to warm up to RT, stirred for further 10 minutes and cooled to 0 °C. A solution of 1,1,1,2,2,3,3,4,4,5,5,6,6,7,7,8,8-heptadecafluoro-10-iodoundecane (0.92 g, 1.55 mmol) in dry THF (3 mL) was added dropwise under stirring. The mixture was allowed to warm up to RT, stirred for further 60 minutes and cooled to 0 °C. A second portion of *t*-BuOK (0.18 g, 1.60 mmol) dissolved in dry THF (3 mL) was added dropwise. After warming up to RT and cooling again to 0 °C as described above, a second portion of 1,1,1,2,2,3,3,4,4,5,5,6,6,7,7,8,8-heptadecafluoro-10-iodoundecane (0.92 g, 1.55 mmol) in dry THF (3 mL) was added dropwise under stirring. The mixture was allowed to warm up to RT and stirred overnight. Water (20 mL) was slowly added and the mixture was extracted with hexane (3 x 15 mL). The combined organic layers were washed with brine, dried over $MgSO_4$ and evaporated to dryness. The yellowish residue was purified by flash column chromatography (silica gel, hexane). The title compound was obtained as a white solid (1.45 g, yield = 72%). ^{1}H NMR (300 MHz, $CDCl_3$) δ 7.72 (d, *J* = 8.0 Hz, 2H), 7.65 (d, *J* = 1.4 Hz, 2H), 7.45 (d, *J* = 8.0 Hz, 2H), 2.04 (t, J = 8.2 Hz, 4H), 1.91–1.76 (m, 4H), 0.97–0.86 (m, 4H). ^{13}C NMR (75 MHz, $CDCl_3$) δ 150.32, 139.92, 137.98, 131.17, 122.14, 118.70–108.33 (m, C_8F_{17}), 93.67, 55.07, 39.49, 30.75 (t, J_{CF} = 22 Hz), 14.92. ^{19}F NMR (282 MHz, $CDCl_3$) δ -81.5 (t, *J* = 9.6 Hz, 6F), -115.1 (br s, 4F), -122.4–-122.6 (m, 12F), -123.4 (br s, 4F), -124.3 (br s, 4F), -126.8 (br s, 4F). Anal. calcd for $C_{35}H_{18}F_{34}I_2$: C, 31.41; H, 1.36; found: C, 31.43; H, 1.40.

9,9-Bis(((1,1,1,3,3,3-hexafluoro-2-(trifluoromethyl)propan-2-yl)oxy)methyl)-9H-fluorene (**6**). In a flame dried Schlenk tube 9,9-bis(hydroxymethyl)fluorene (1.13 g, 5.00 mmol), perfluoro-*tert*-butanol (1.95 mL, 14.00

mmol) and PPh_3 (3.67 g, 14.00 mmol) were dissolved under nitrogen in dry THF (30 mL). To the stirred solution cooled at 0 °C, diisopropyl azodicarboxylate (2.76 mL, 14.00 mmol) was added dropwise by a syringe in 10 minutes. The stirred reaction mixture was allowed to warm up to RT. After 2h it was brought to 45 °C for 24 h. The volatiles were then removed under reduced pressure and the residue was purified by column chromatography (silica gel, hexane). The title compound was obtained as a white solid (2.81 g, yield = 85%). ^{1}H NMR (300 MHz, $CDCl_3$) δ 7.79 (d, *J* = 7.6 Hz, 2H), 7.57 (d, *J* = 7.6 Hz, 2H), 7.48 (td, *J* = 7.5, 1,1 Hz, 2H), 7.33 (td, *J* = 7.5, 1.1 Hz, 2H), 4.25 (s, 4H). ^{13}C NMR (101 MHz, $CDCl_3$) δ 142.83, 140.79, 129.35, 127.74, 125.70, 120.49, 120.43 (q, J_{CF} = 290 Hz), 79.76 (m), 69.67, 53.98. ^{19}F NMR (282 MHz, $CDCl_3$): δ –70.5 (s). Anal. calcd for $C_{23}H_{12}F_{18}O_2$: C, 41.71; H, 1.83; found: C, 41.74; H, 1.85.

*9,9-Bis(((1,1,1,3,3,3-hexafluoro-2-(trifluoromethyl)propan-2-yl)oxy)methyl)-2,7-diiodo-9H-fluoren*e (**3**). Iodine (0.96 g, 3.78 mmol) and iodic acid (0.41 g, 2.36 mmol) were added to a mixture of fluorene **6** (3.31 g, 5.00 mmol) in 96% sulfuric acid (0.3 mL), carbon tetrachloride (0.4 mL) and acetic acid (35.0 mL). The reaction mixture was refluxed for 4 h then allowed to cool to room temperature. Upon cooling a pale pink solid precipitated out. The precipitate was recovered on a Büchner funnel, thoroughly washed with water, dried in vacuo and purified by column chromatography (silica gel, petroleum ether). The title compound was recovered as a white solid (3.25 g, yield = 71%). ^{1}H NMR (400 MHz, $CDCl_3$) δ 7.89 (d, *J* = 1.4 Hz, 2H), 7.82 (dd, *J* = 8.0, 1.5 Hz, 2H), 7.50 (d, *J* = 8.0 Hz, 2H), 4.18 (s, 4H). ^{13}C NMR (101 MHz, $CDCl_3$) δ 144.16, 139.40, 138.63, 135.13, 122.20, 120.33 (q, J_{CF} = 292 Hz), 93.27, 79.98 (m,), 68.95, 53.92. ^{19}F NMR (282 MHz, $CDCl_3$): δ –70.5 (s). Anal. calcd for $C_{23}H_{10}F_{18}I_2O_2$: C, 30.22; H, 1.10; found: C, 30.23; H, 1.12.

2,2'-(((2,7-diiodo-9H-fluorene-9,9-diyl)bis(ethane-2,1-diyl))bis(oxy))bis(tetrahydro-2H-pyran) (**7**). To a solution of 2,7-diiodofluorene (2.51 g, 6.00 mmol), 2-(2-bromoethoxy)tetrahydro-2*H*-pyran (3.26 g, 15.60 mmol) and tetrabutylammonium bromide (0.19 g, 0.60 mmol) in toluene (10 mL), a 50% wt NaOH aqueous solution (5 mL) was added. The reaction mixture was then stirred at 100 °C for 10 h. After cooling to RT the mixture was extracted with EtOAc (3 x 10 mL). The combined organic layers were washed with water, brine, and dried over $MgSO_4$. The solution was concentrated under vacuum. The crude oily product was purified by column chromatography (silica gel, hexane/ EtOAc from 10/1 to 8/1) to give the title compound as a thick brownish oil (3.12 g, yield = 77%). ^{1}H NMR (300 MHz, $CDCl_3$) δ 7.74 (s, 2H), 7.64 (d, *J* = 8.0 Hz, 2H), 7.38 (d, *J* = 8.0 Hz, 2H), 4.16–4.08 (m, 2H), 3.55–3.40 (m, 2H), 3.32–3.20 (m, 2H), 3.14 (dt, *J* = 9.8, 7.0 Hz, 2H), 2.71 (dt, *J* = 9.8, 7.0 Hz, 2H), 2.34 (t, *J* = 6.9 Hz, 4H), 1.66–1.20 (m, 12H). ^{13}C NMR (75 MHz, $CDCl_3$) δ 151.15,

139.45, 136.47, 132.83, 121.68, 98.87, 93.29, 63.46, 62.05, 52.33, 39.99, 30.57, 25.44, 19.44. Anal. calcd for $C_{27}H_{32}F_{34}I_2O_4$: C, 48.09; H, 4.78; found: C, 48.05; H, 4.81.

2,2'-(2,7-Diiodo-9H-fluorene-9,9-diyl)diethanol (**8**). To a solution of protected fluorene **7** (3.10 g, 4.60 mmol) in EtOH (15 mL) 10% aqueous HCl (1 mL) was added. and then the reaction mixture was refluxed for 30 min. After cooling, the solvent was removed under reduced pressure. The residue was dissolved in DCM (20 mL) and the solution was washed with water, saturated aqueous $NaHCO_3$ solution, water, brine, then dried over $MgSO_4$. The solvent was removed under reduced pressure. The residue was dissolved in DCM (5 mL) and the solution was treated with petroleum ether (10 mL). The title compound precipitated out and could be recovered by filtration as a white solid (1.55 g, yield = 67%). ^{1}H NMR (300 MHz, $CDCl_3$) δ 7.76 (d, *J* = 1.5 Hz, 2H), 7.69 (dd, *J* = 8.0, 1.5 Hz, 2H), 7.43 (d, *J* = 8.0 Hz, 2H), 3.02 (t, *J* = 7.4 Hz, 4H), 2.32 (t, *J* = 7.3 Hz, 4H). ^{13}C NMR (75 MHz, $CDCl_3$) δ 150.77, 139.20, 137.03, 132.49, 122.05, 93.64, 58.82, 52.09, 42.69. Anal. calcd for $C_{27}H_{32}F_{34}I_2O_4$: C, 40.34; H, 3.19; found: C, 40.35; H, 3.21.

9,9-Bis(2-((1,1,1,3,3,3-hexafluoro-2-(trifluoromethyl)propan-2-yl)oxy)ethyl)-2,7-diiodo-9H-fluorene (**4**). Fluorene diol **8** (1.52 g, 3.0 mmol) and PPh_3 (3.15 g, 12.0 mmol) were dissolved under nitrogen in dry THF (25 mL). To the stirred solution cooled at 0 °C, diisopropyl azodicarboxylate (2.36 mL, 12.00 mmol) was added dropwise by a syringe. After 10 minutes the reactor was brought to RT and perfluoro-*tert*-butanol (1.67 mL, 12.00 mmol) was added dropwise by a syringe. The reaction was continued until TLC showed the complete disappearance of the starting material (36 h). The solvent was the removed under reduced pressure and the residue was purified by column chromatography (silica gel, hexane to hexane/DCM 10/1) DCM 7/3). The title compound was obtained as a white solid (1.72 g, yield = 61%). ^{1}H NMR (300 MHz, $CDCl_3$) δ 7.72–7.69 (m, 4H), 7.43 (d, *J* = 7.9 Hz, 2H), 3.40 (t, *J* = 6.4 Hz, 4H), 2.41 (t, *J* = 6.4 Hz, 4H).^{13}C NMR (75 MHz, $CDCl_3$): δ 148.83, 139.36, 137.43, 132.37, 122.06, 120.11 (q, JCF = 292 Hz), 93.49, 79.50 (m), 65.87, 51.37, 39.66. ^{19}F NMR (282 MHz, CDCl3): δ [ppm] = –71.0 (s). Anal. calcd for $C_{25}H_{14}F_{18}I_2O_2$: C, 31.87; H, 1.50; found: C, 31.87; H, 1.53.

9,9'-(9,9-Bis(4,4,5,5,6,6,7,7,8,8,9,9,10,10,11,11,11-heptadecafluoroundecyl)-9H-fluorene-2,7-diyl)bis(9H-carbazole) (**CFC-F2**). A flame-dried Schlenk tube equipped with a magnetic stir bar was charged with fluorene **2** (1.34 g, 1.00 mmol), carbazole (0.50 g, 3.00 mmol), dry K_2CO_3 (1.07 g, 7.74 mmol), copper bronze (0.39 g, 6.00 mmol), 18-crown-6 (79 mg, 0.30 mmol) and dry 1,2-dichlorobenzene (10 mL) under nitrogen atmosphere. The reaction mixture was deaerated by freeze-pump-thaw cycles (3 times), purged with nitrogen and heated at 180 °C under stirring for 60h. The mixture was cooled to RT, diluted with

DCM (30 mL) and filtered through a short pad of Celite. The organic solvents were removed by evaporation under vacuum and the crude product was purified by column chromatography (silica gel, petroleum ether/DCM 6/1). The title compound was recovered as a white solid (1.38 g, yield = 97%). ^{1}H NMR (400 MHz, CD_2Cl_2) δ 8.19 (d, *J* = 7.8 Hz, 4H), 8.09 (d, *J* = 8.0 Hz, 2H), 7.69 (dd, *J* = 8.0, 1.4 Hz, 2H), 7.65 (d, *J* = 1.4 Hz, 2H), 7.49–7.41 (m, 8H), 7.36–7.29 (m, 4H), 2.27–2.15 (m, 4H), 2.06–1.80 (m, 4H), 1.32–1.17 (m, 4H). ^{13}C NMR (101 MHz, CD_2Cl_2) δ 151.25, 141.44, 140.14, 137.69, 127.37, 126.45, 123.81, 122.18, 122.13, 120.74, 120.50, 110.05, 118.98–109.03 (m, C_8F_{17}), 55.70, 39.88, 31.34 (t, J_{CF} = 22 Hz), 15.69. ^{19}F NMR (377 MHz, $CDCl_3$) δ -81.7 (t, *J* = 9.5 Hz, 6F), -115.4 (br s, 4F), -122.5–-123.0 (m, 12F), -123.5 (br s, 4F), -124.6 (br s, 4F), -126.9 (br s, 4F). Anal. calcd for $C_{59}H_{34}F_{34}N_2$: C, 50.01; H, 2.42, N, 1.98; found: C, 50.00; H, 2.45, N, 1.95.

9,9'-(9,9-Bis(((1,1,1,3,3,3-hexafluoro-2-(trifluoromethyl)propan-2-yl)oxy)methyl)-9H-fluorene-2,7-diyl)bis(9H-carbazole) (**CFC-F3**). The title compound was prepared from fluorene **3** following the procedure described for **CFC-F2**. Column chromatography (silica gel, petroleum ether/DCM 5/1). White solid (0.75 g, yield = 76%). ^{1}H NMR (400 MHz, $CDCl_3$) δ 8.20 (d, *J* = 7.7 Hz, 4H), 8.09 (d, *J* = 8.0 Hz, 2H), 7.80 (d, *J* = 1.6 Hz, 2H), 7.74 (dd, *J* = 8.0, 1.8 Hz, 2H), 7.46–7.40 (m, 4H), 7.38–7.30 (m, 8H), 4.45 (s, 4H). ^{13}C NMR (101 MHz, $CDCl_3$) δ 144.92, 141.21, 139.54, 137.74, 129.23, 126.17, 124.86, 123.63, 122.00, 120.54, 120.36, 120.26 (q, J_{CF} = 293 Hz), 109.71, 79.83 (m), 69.69, 54.95. ^{19}F NMR (282 MHz, $CDCl_3$) δ –71.3 (s). Anal. calcd for $C_{47}H_{26}F_{18}N_2O_2$: C, 56.87; H, 2.64; N, 2.82; found: C, 56.83; H, 2.67, N, 2.83.

9,9'-(9,9-Bis(3-((1,1,1,3,3,3-hexafluoro-2-(trifluoromethyl)propan-2-yl)oxy)ethyl)-9H-fluorene-2,7-diyl)bis(9H-carbazole) (**CFC-F4**). The title compound was prepared from fluorene **4** following the procedure described for **CFC-F2**. Column chromatography (silica gel, hexane/EtOAc 60/1). White solid (0.85 g, yield = 83%). ^{1}H NMR (400 MHz, CD_2Cl_2) δ 8.20 (d, *J* = 7.8 Hz, 4H), 8.07 (d, *J* = 8.5 Hz, 2H), 7.74–7.65 (m, 4H), 7.53–7.40 (m, 8H), 7.37–7.27 (m, 4H), 3.68 (t, *J* = 6.7 Hz, 1H), 2.60 (t, *J* = 6.8 Hz, 1H). ^{13}C NMR (101 MHz, CD_2Cl_2) δ 149.72, 141.42, 139.62, 137.81, 127.69, 126.46, 123.83, 122.41, 122.11, 120.74, 120.53, 120.54 (q, J_{CF} = 292 Hz), 109.97, 79.82 (m), 66.67, 51.68, 40.06. ^{19}F NMR (282 MHz, $CDCl_3$): δ –71.4 (s). Anal. calcd for $C_{49}H_{30}F_{18}N_2O_2$: C, 57.66; H, 2.96; N, 2.74; found: C, 57.67; H, 2.96, N, 2.71.

9,9'-(9,9-Bis(3-((1,1,1,3,3,3-hexafluoro-2-(trifluoromethyl)propan-2-yl)oxy)propyl)-9H-fluorene-2,7-diyl)bis(9H-carbazole) (**CFC-F5**). The title compound was prepared from 9,9-bis(((1,1,1,3,3,3-hexafluoro-2-(trifluoromethyl)propan-2-yl)oxy)propyl)-2,7-diiodo-9*H*-fluorene following the procedure described for **CFC-F2**. Flash column chromatography (silica gel, petroleum ether/DCM 5/1). White solid (0.84 g, yield = 80%). ^{1}H NMR (400 MHz, CD_2Cl_2) δ 8.20 (d, J = 7.8 Hz, 4H), 8.08 (d, J = 8.0 Hz, 2H), 7.69 (dd, J = 8.0, 1.8 Hz, 2H),

7.66 (d, J = 1.6 Hz, 2H), 7.51–7.43 (m, 8H), 7.38–7.29 (m, 4H), 3.86 (t, J = 6.2 Hz, 4H), 2.28 – 2.18 (m, 4H), 1.35–1.21 (m, 4H). ^{13}C NMR (101 MHz CD_2Cl_2) δ 151.41, 141.34, 140.06, 137.74, 127.18, 126.44, 123.84, 121.96, 121.93, 120.81 (q, J_{CF} = 290 Hz), 120.72, 120.51, 110.07, 80.00 (m), 70.37, 55.10, 36.35, 25.18. ^{19}F NMR (282 MHz, $CDCl_3$): δ –71.3 (s). Anal. calcd for $C_{51}H_{34}F_{18}N_2O_2$: C, 58.40; H, 3.27; N, 1.98; found: C, 58.37; H, 3.28, N, 2.63.

Figures

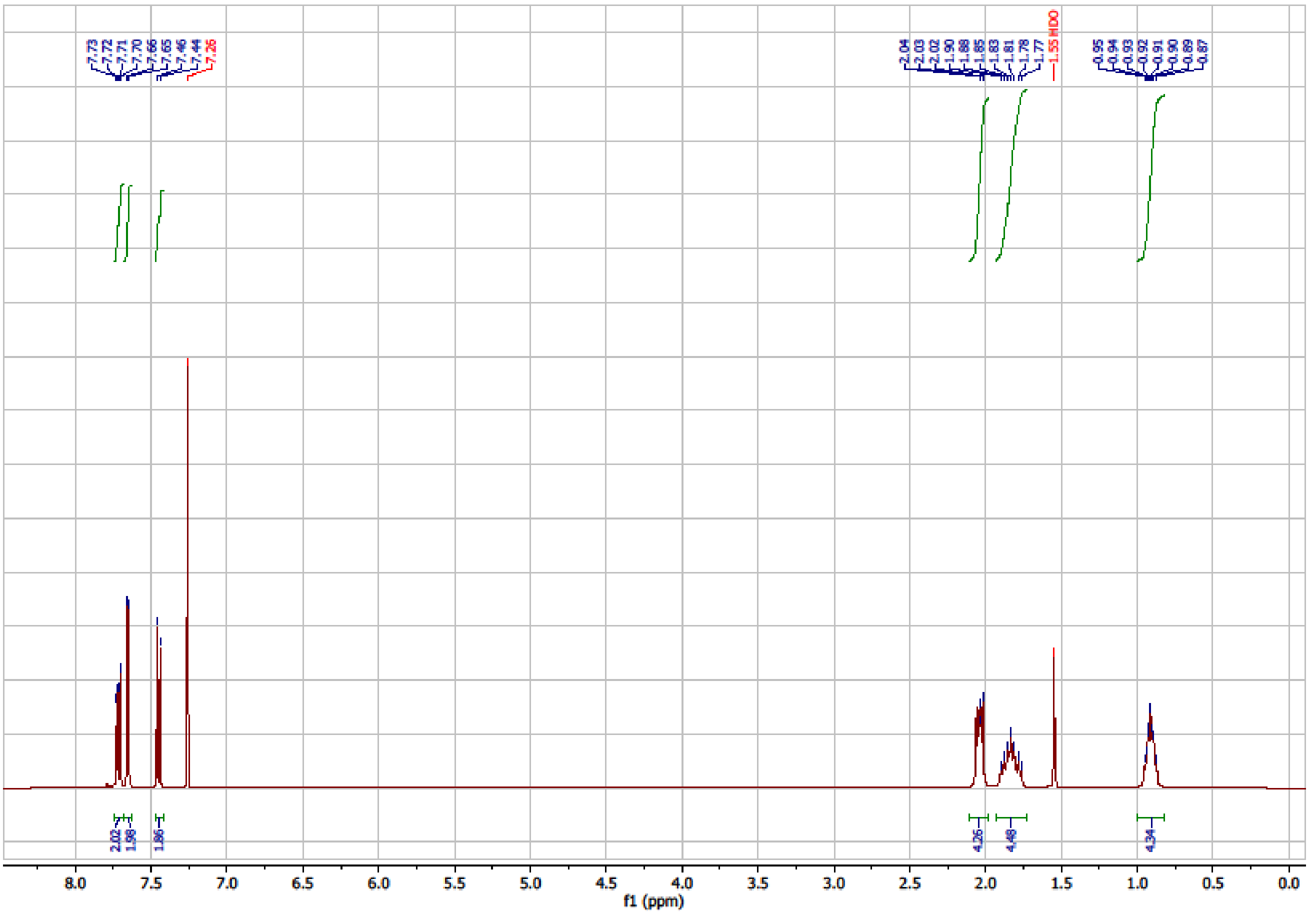

Figure S1.^{1}H NMR of intermediate **2**.

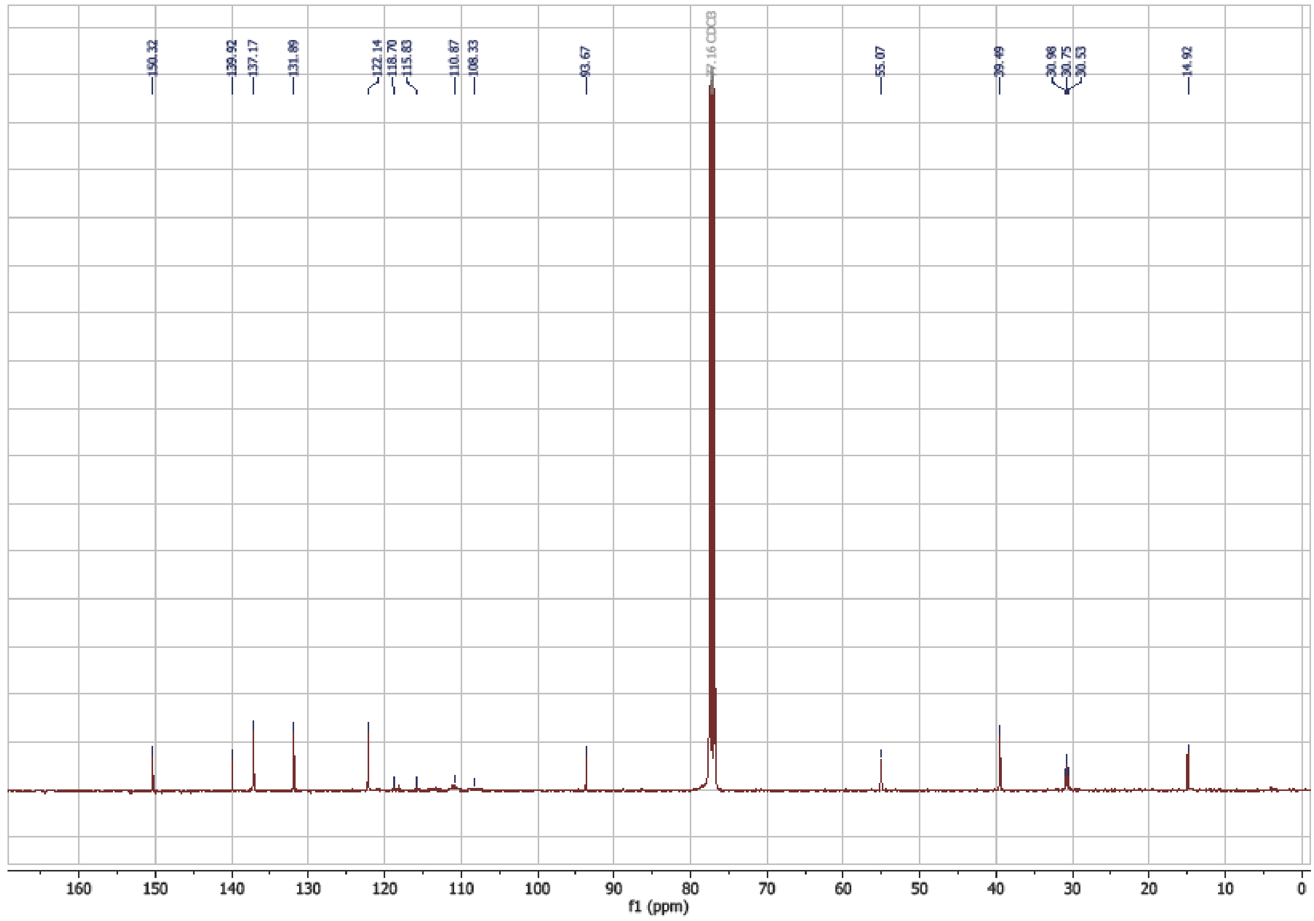

Figure S2. ^{13}C NMR of intermediate **2**.

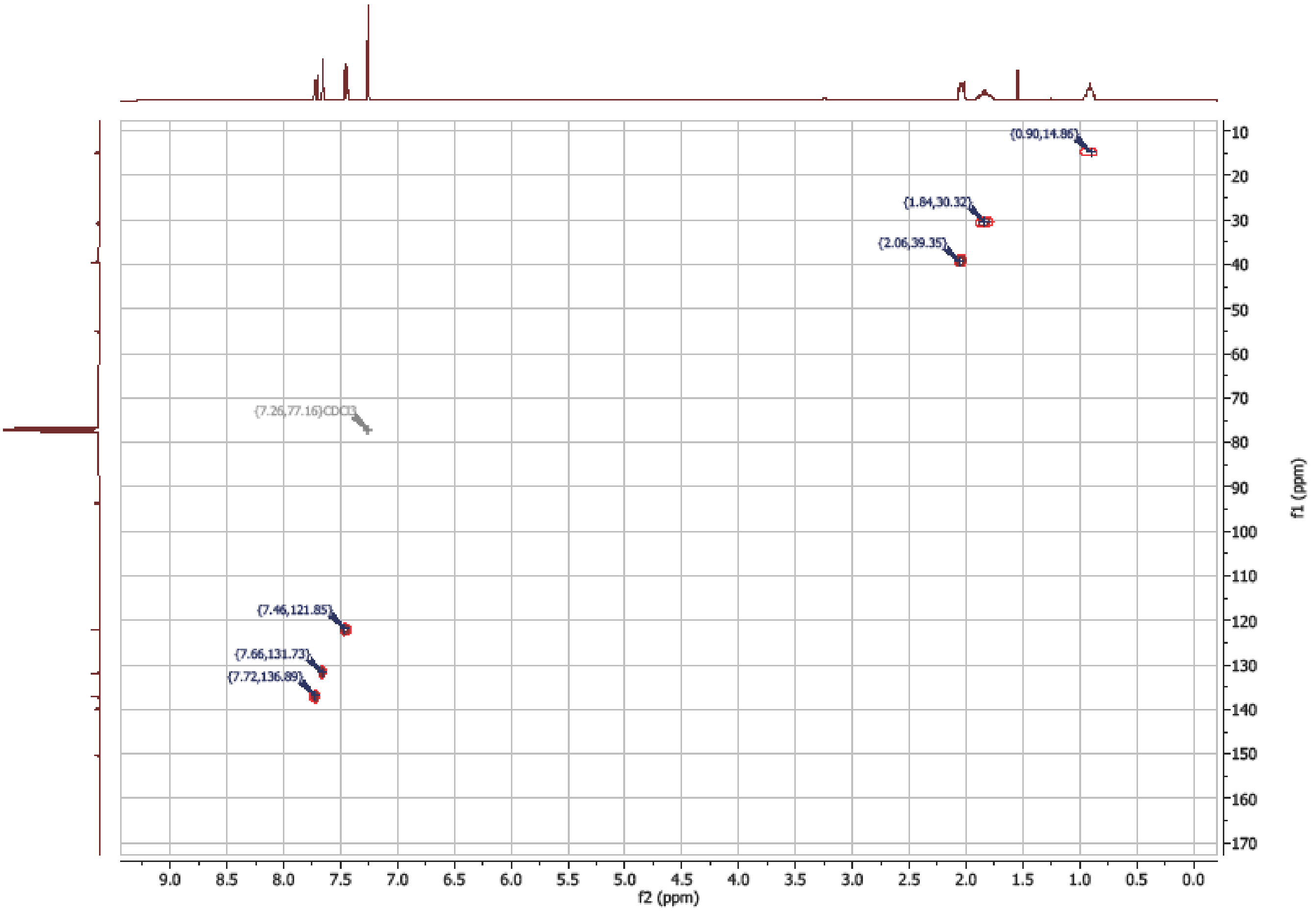

Figure S3. HSQC NMR of intermediate **2**.

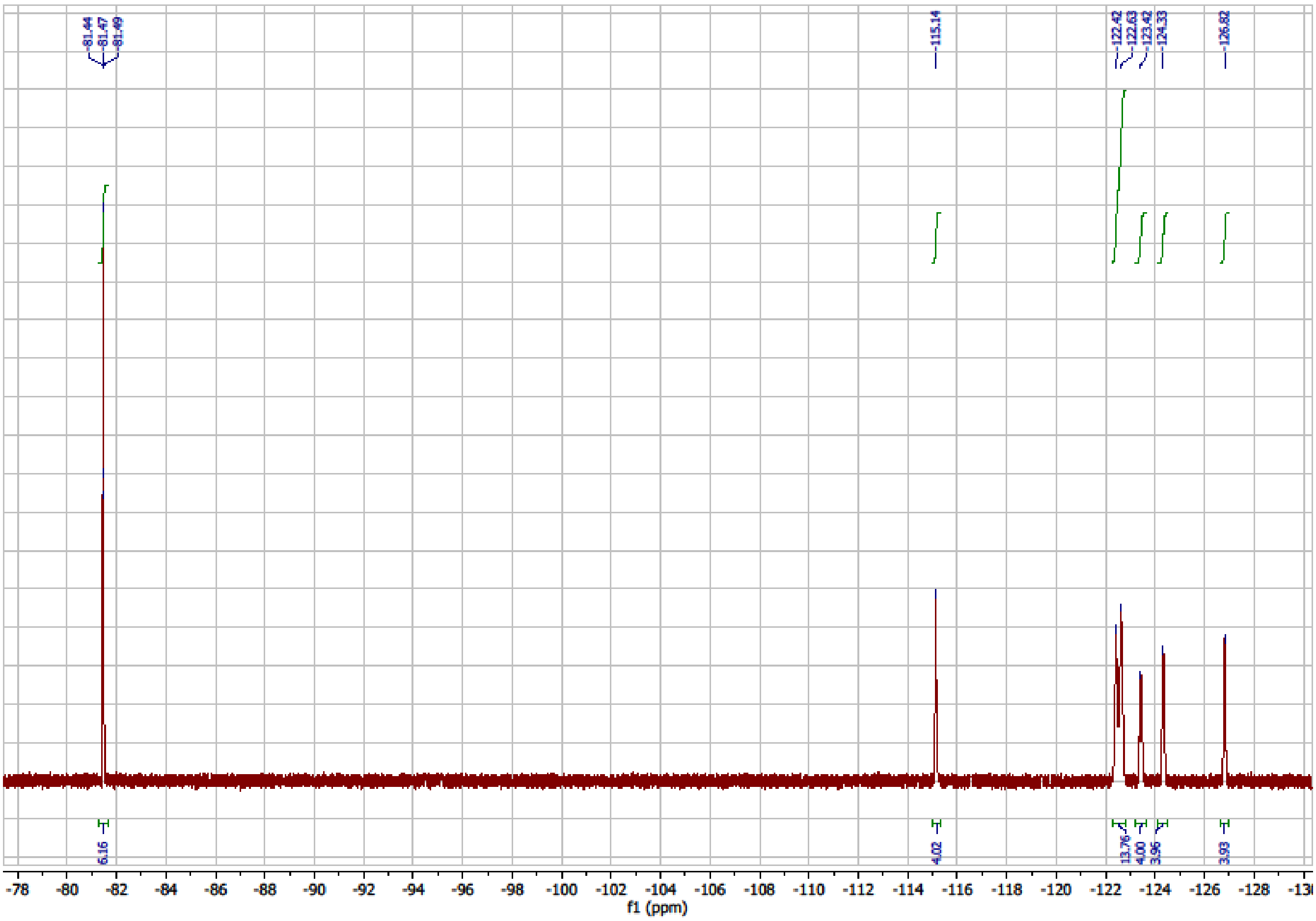

Figure S4. ^{19}F NMR of intermediate **2**

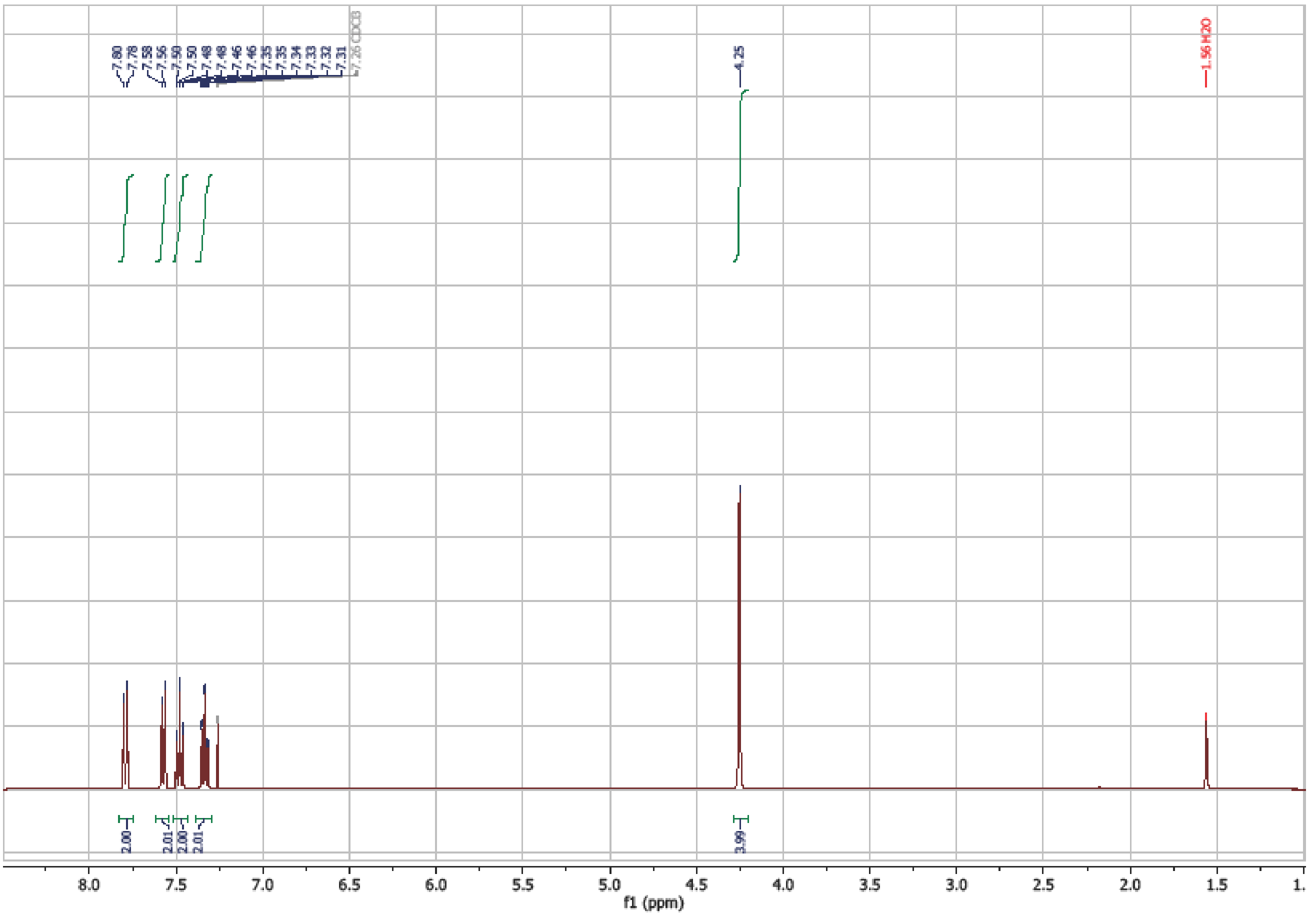

Figure S5. ${}^{1}H$ NMR of intermediate **6**.

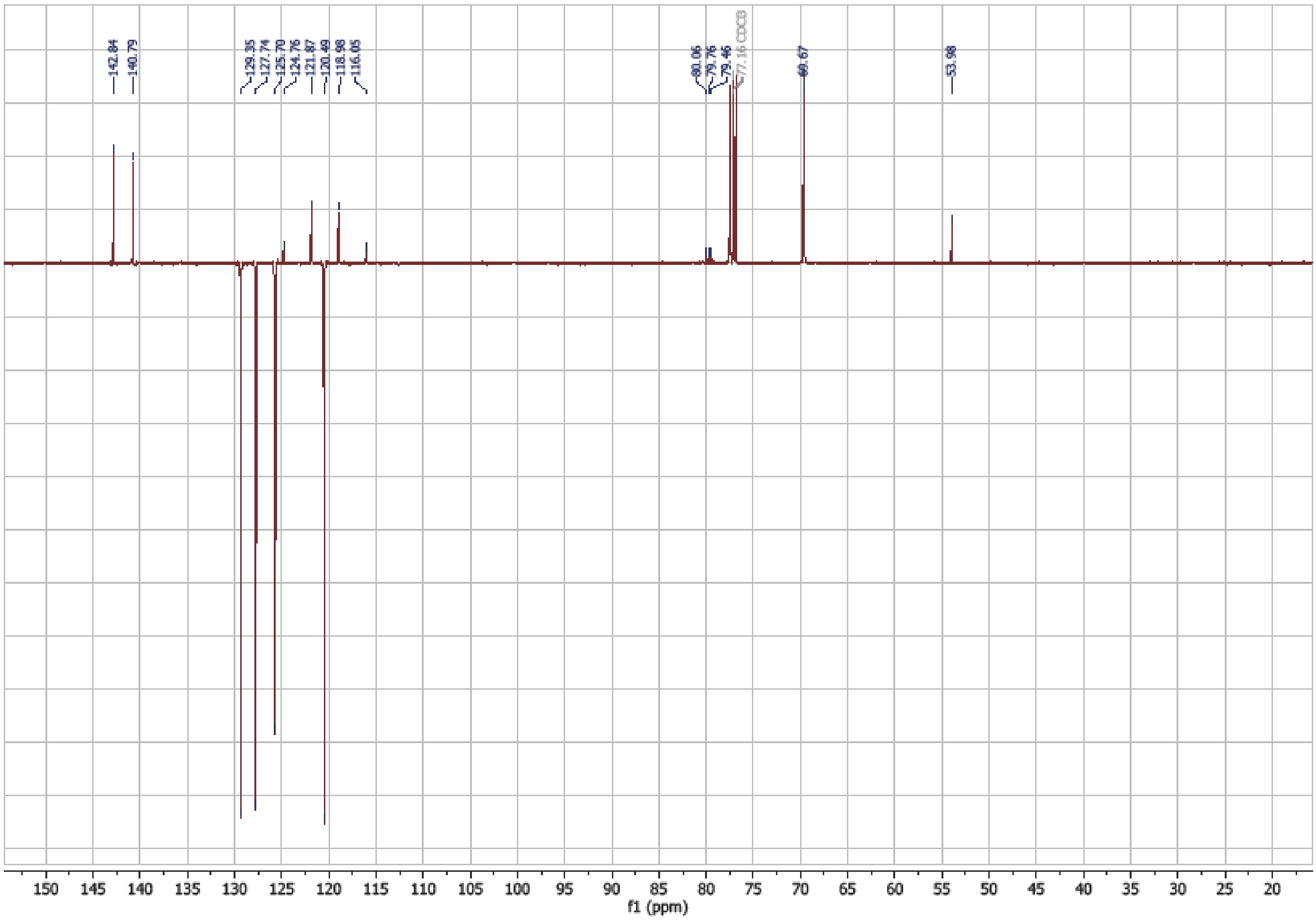

Figure S6. ^{13}C APT NMR of intermediate **6**

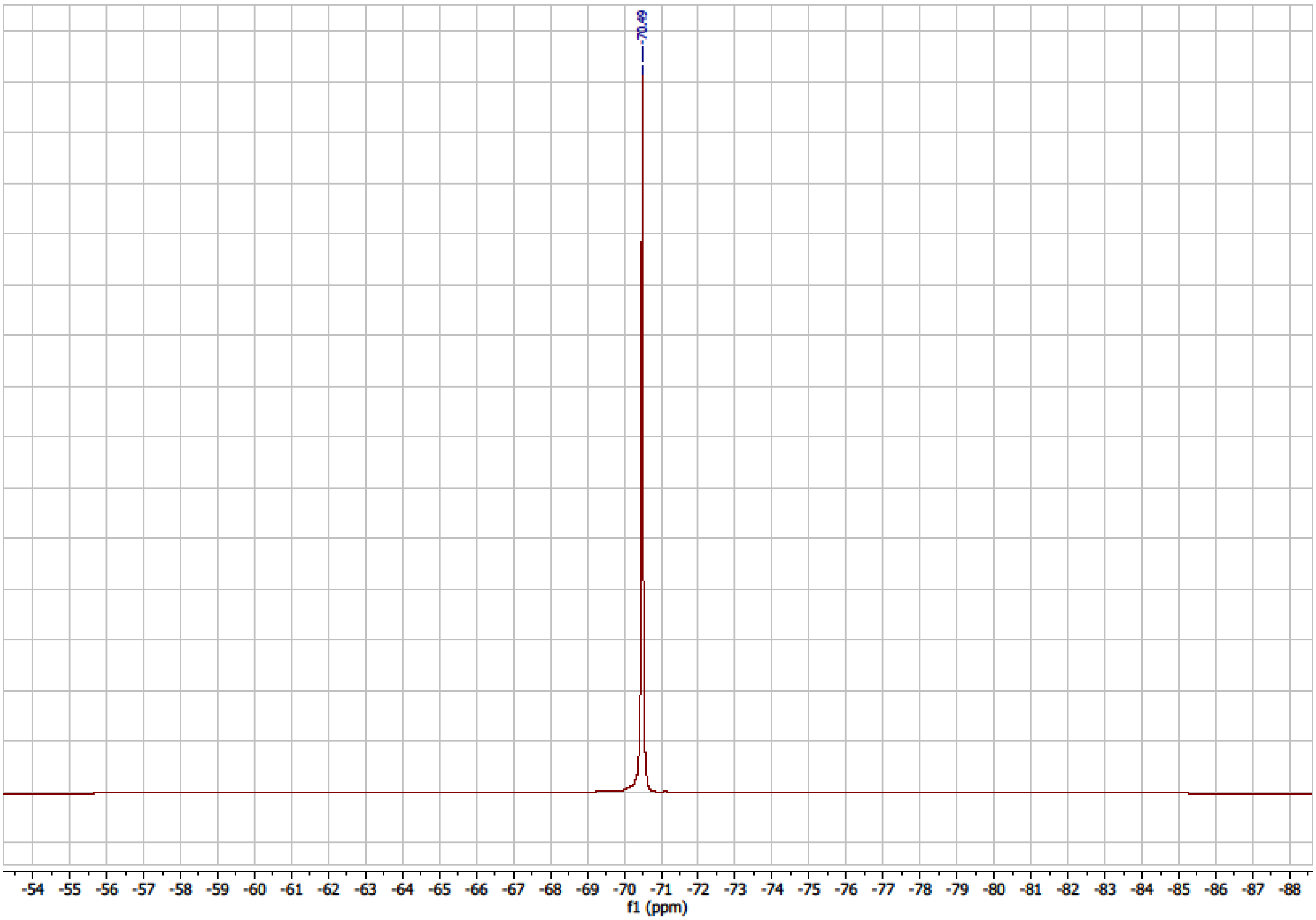

Figure S7. ^{19}F NMR of intermediate **6**

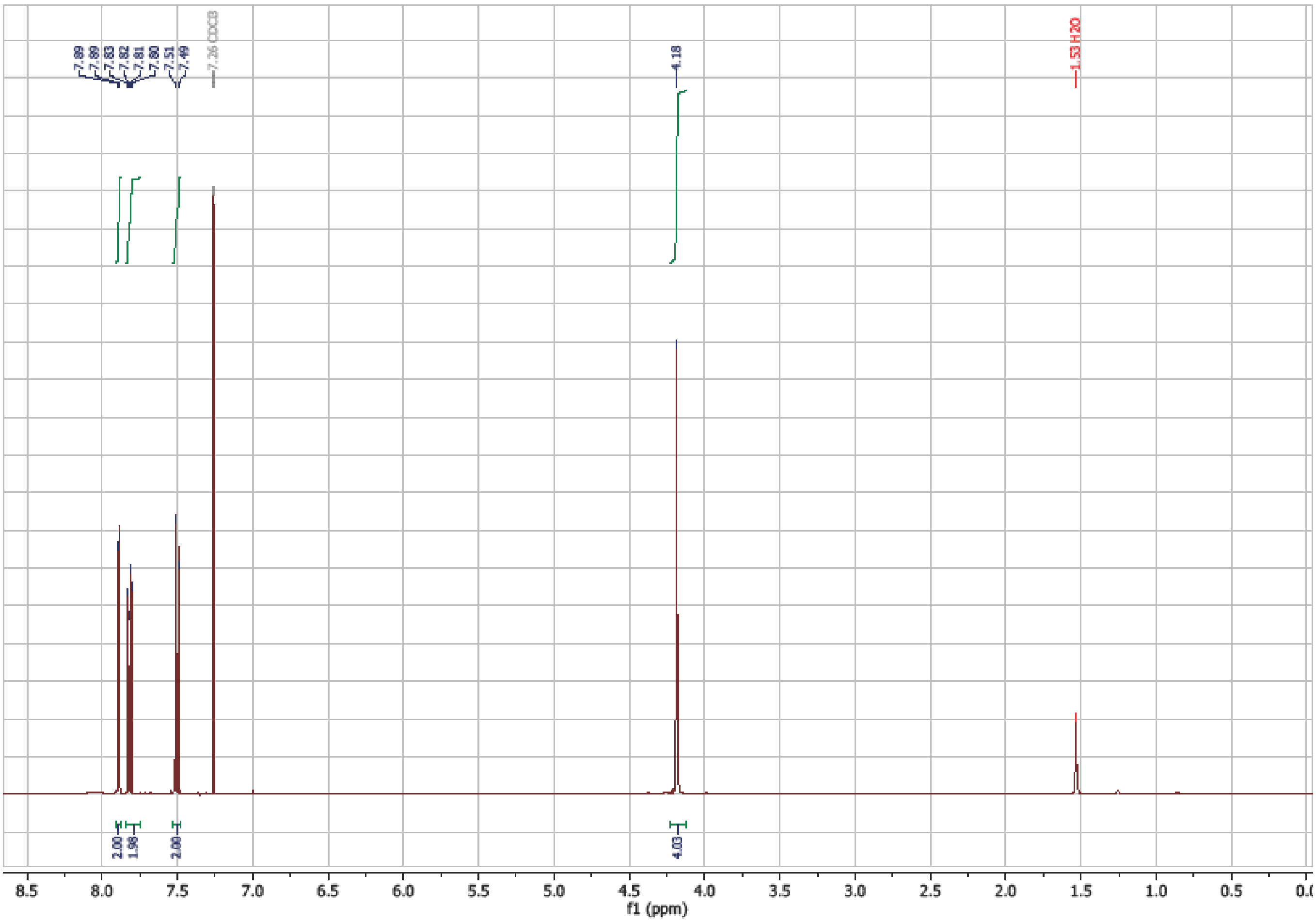

Figure S8.^{1}H NMR of intermediate **3**.

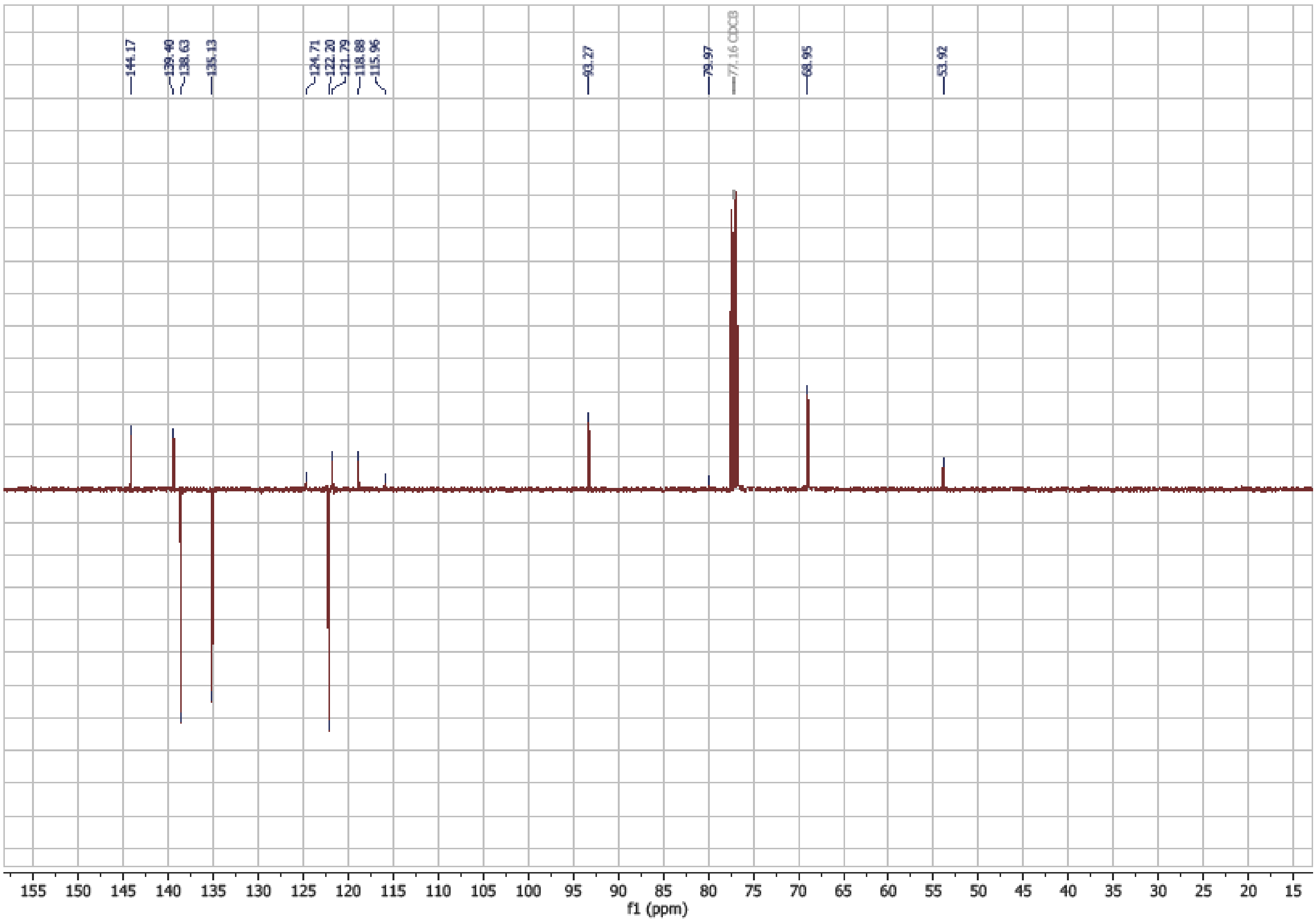

Figure S9. ^{13}C APT NMR of intermediate **3**.

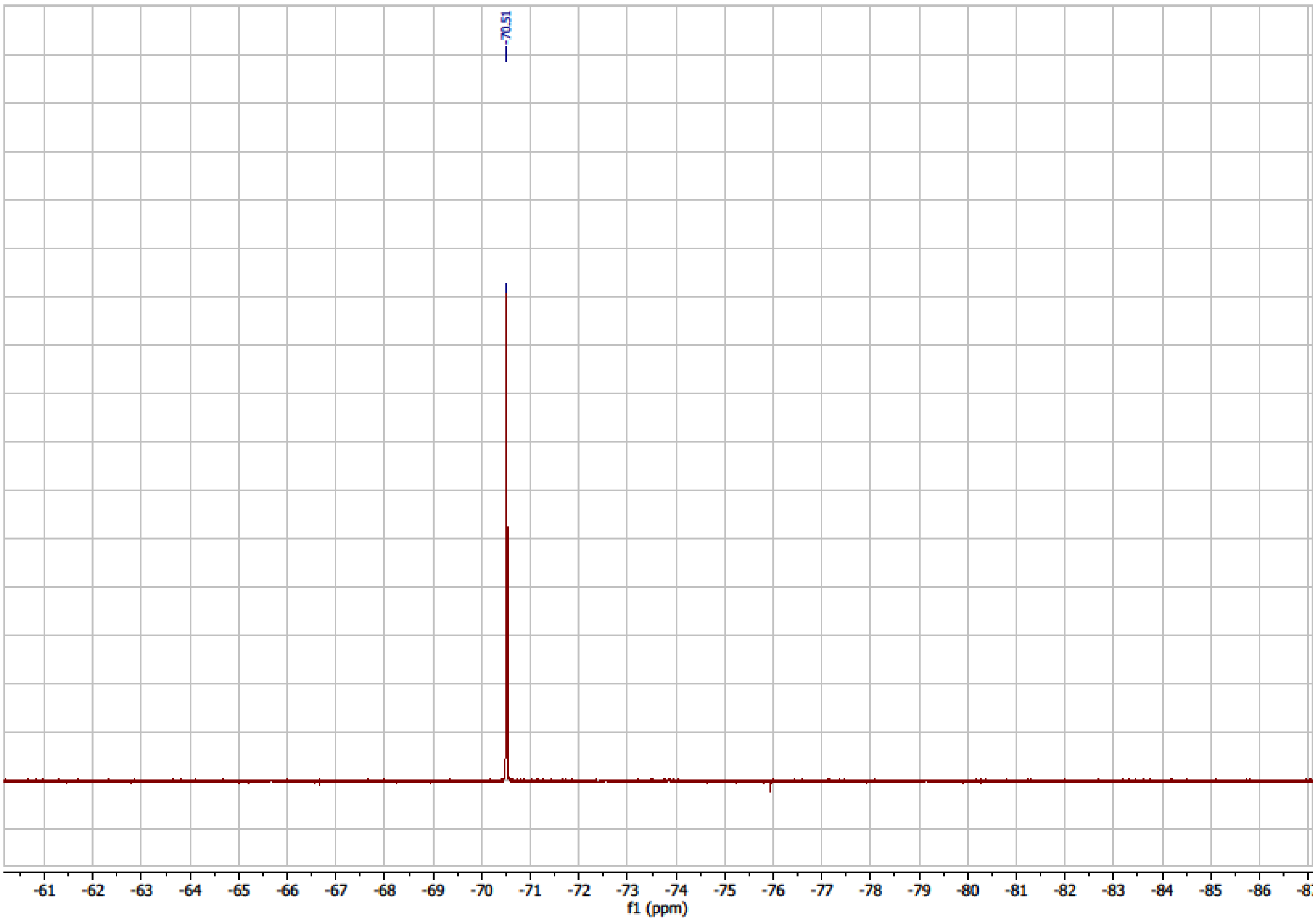

Figure S10. ^{19}F NMR of intermediate **3**.

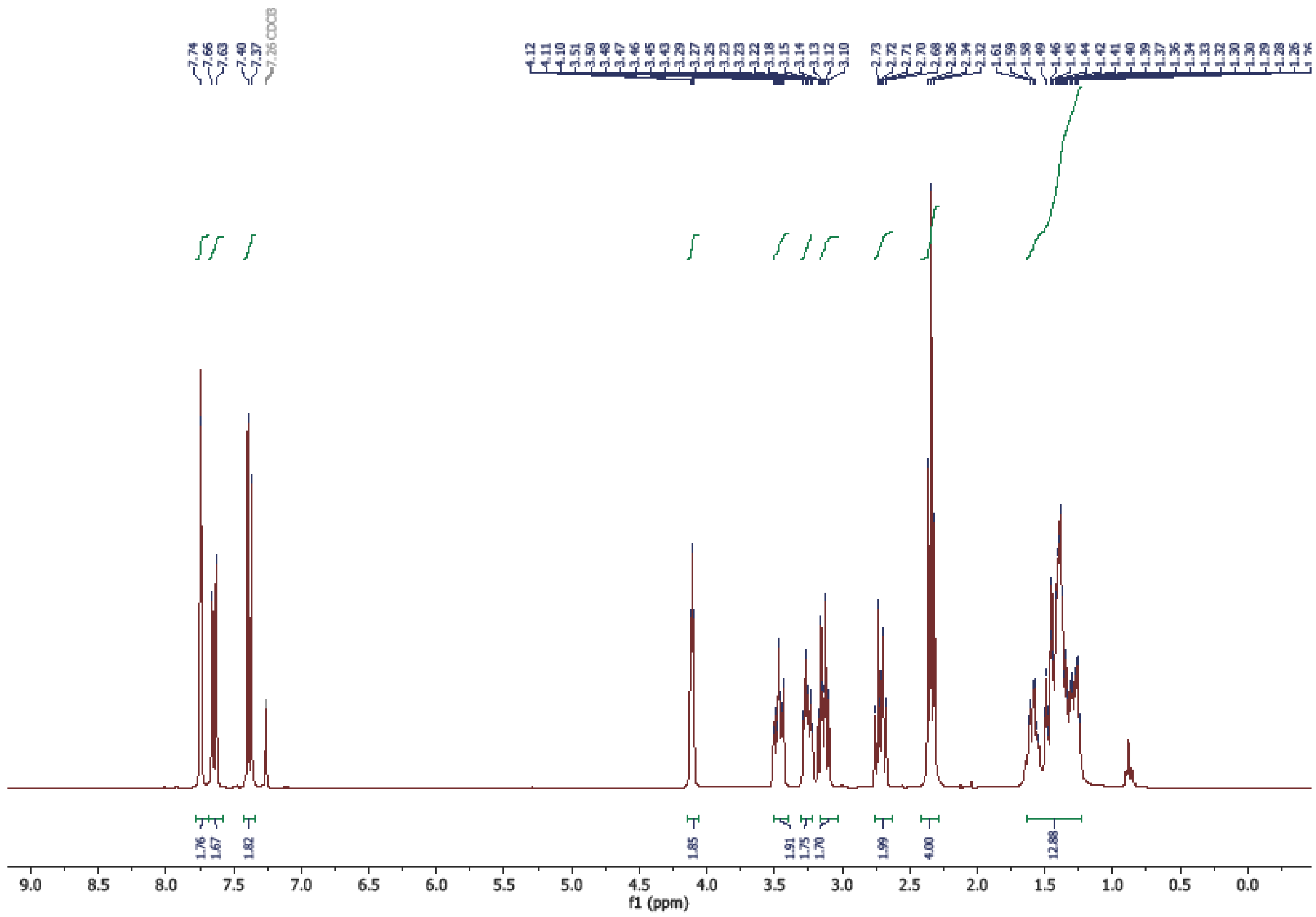

Figure S11.^{1}H NMR of intermediate **7**.

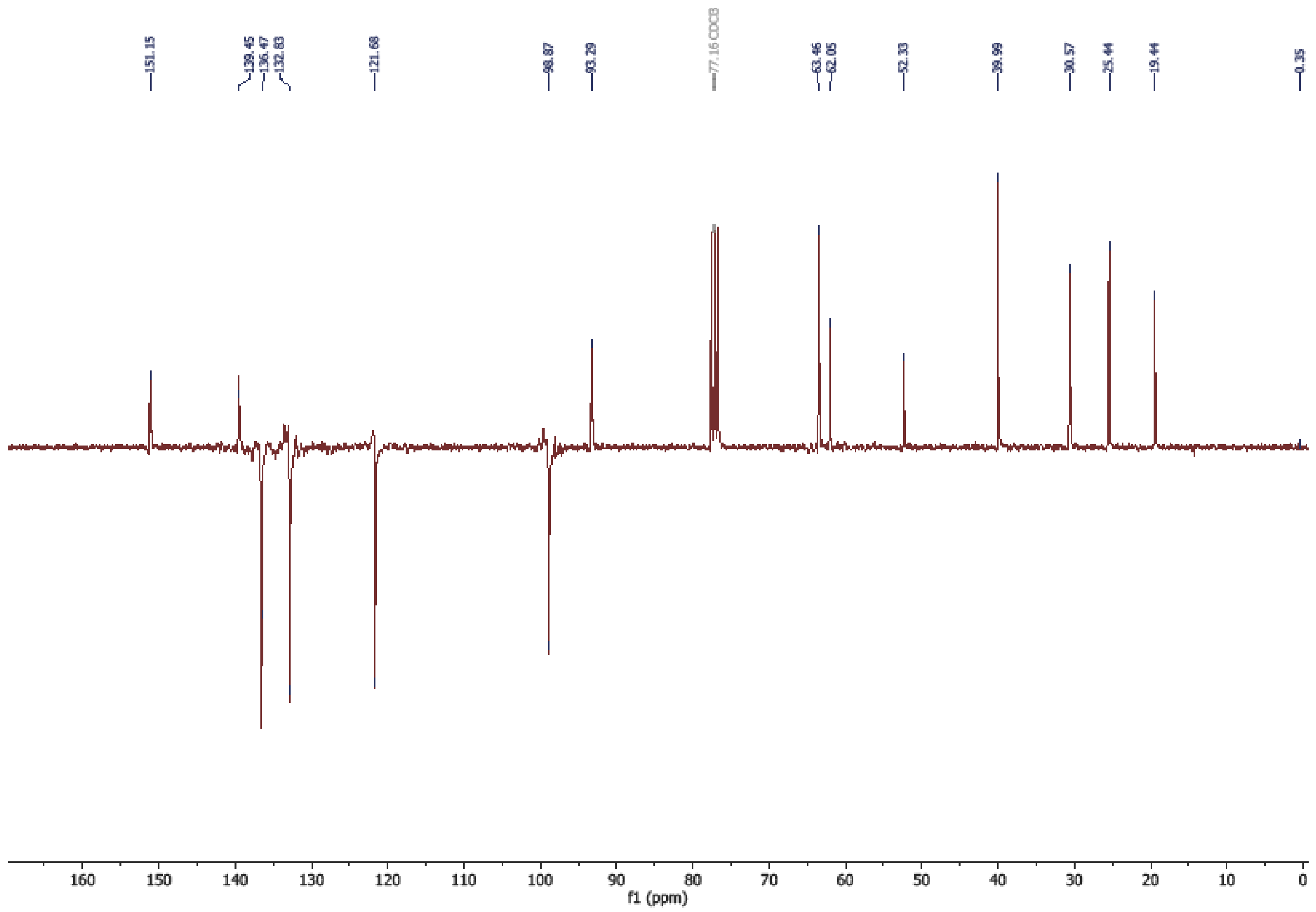

Figure S12.^{13}C APT NMR of intermediate **7**.

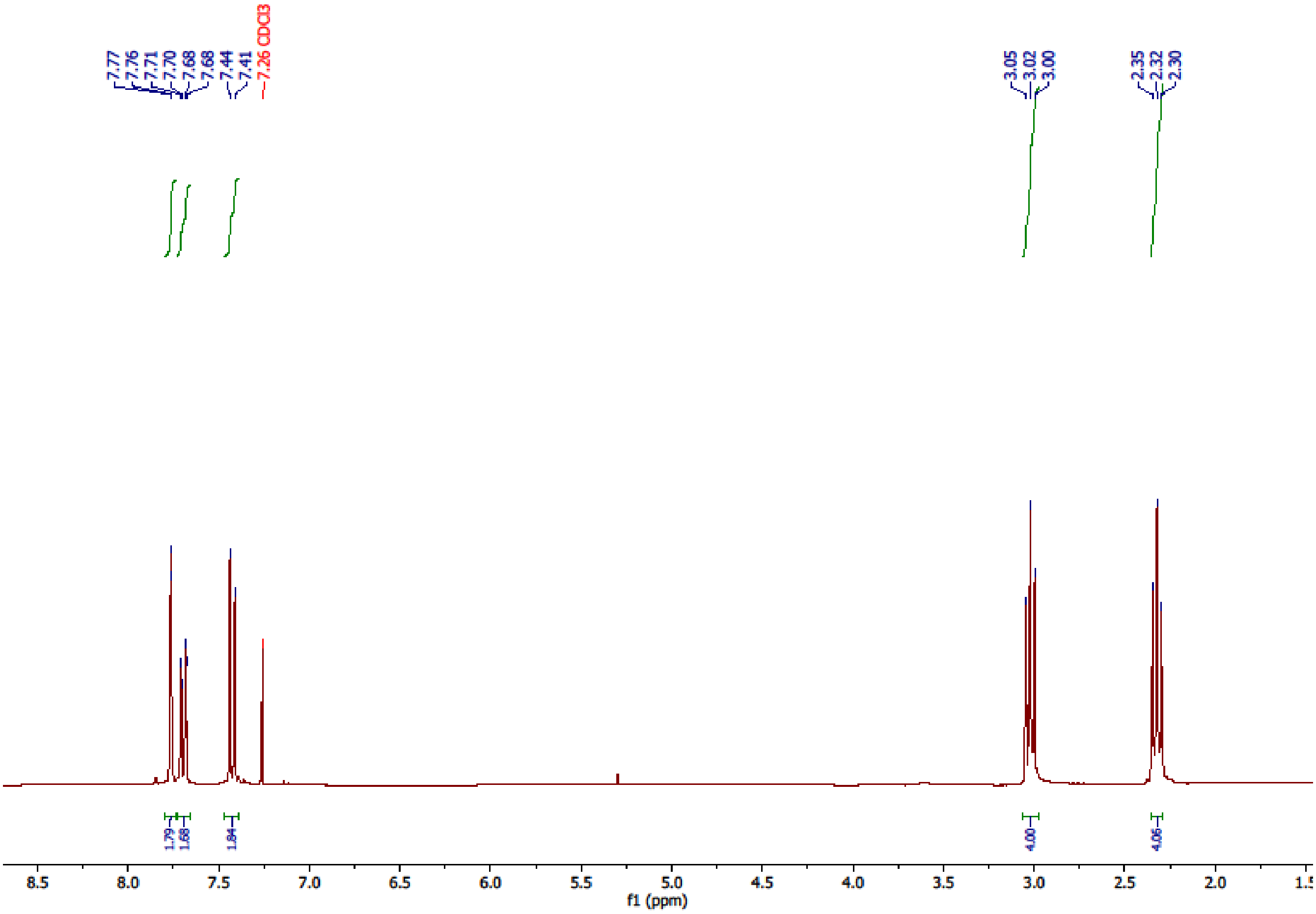

Figure S13.^{1}H NMR of intermediate **8**.

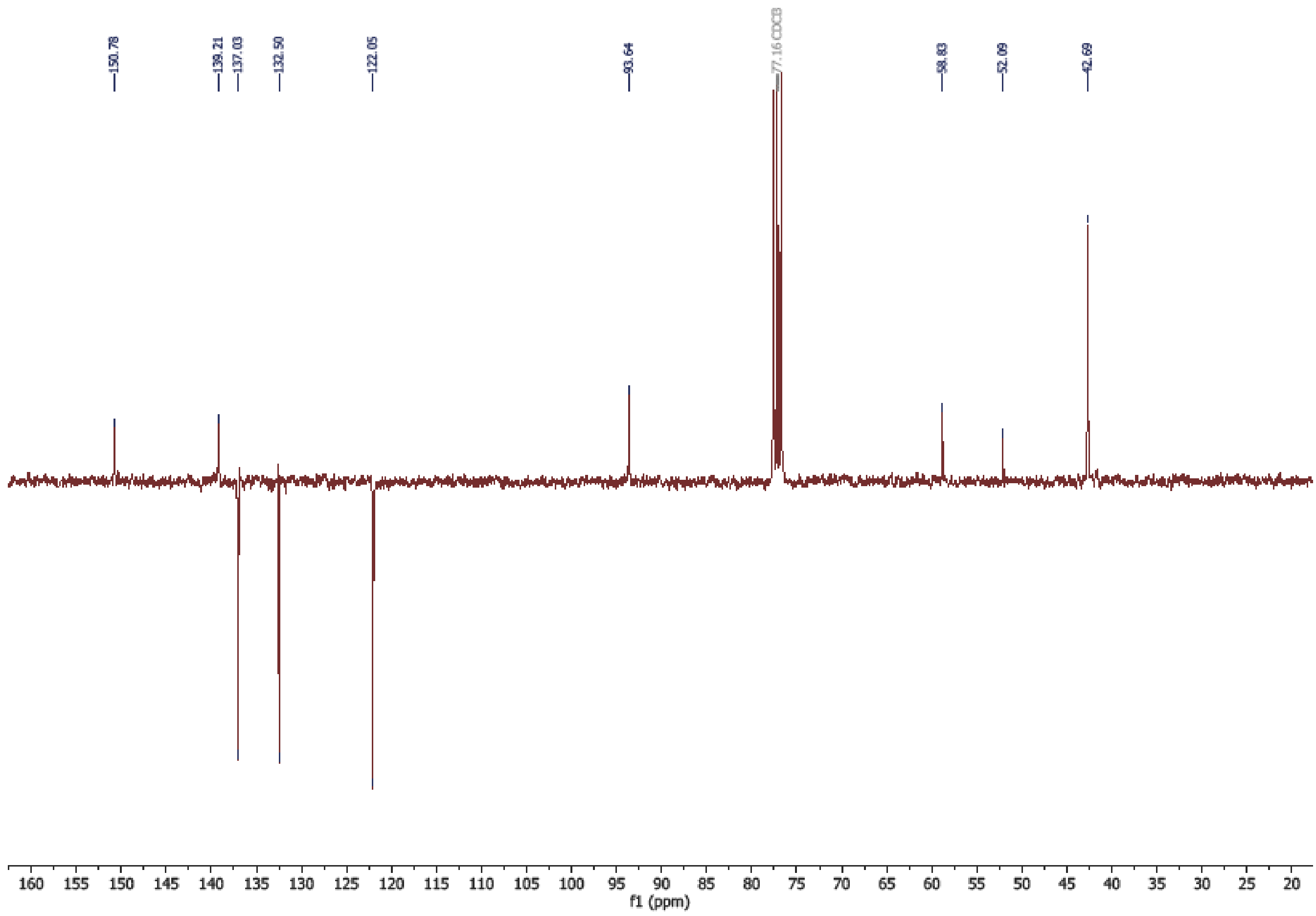

Figure S14.^{13}C APT NMR of intermediate **8**.

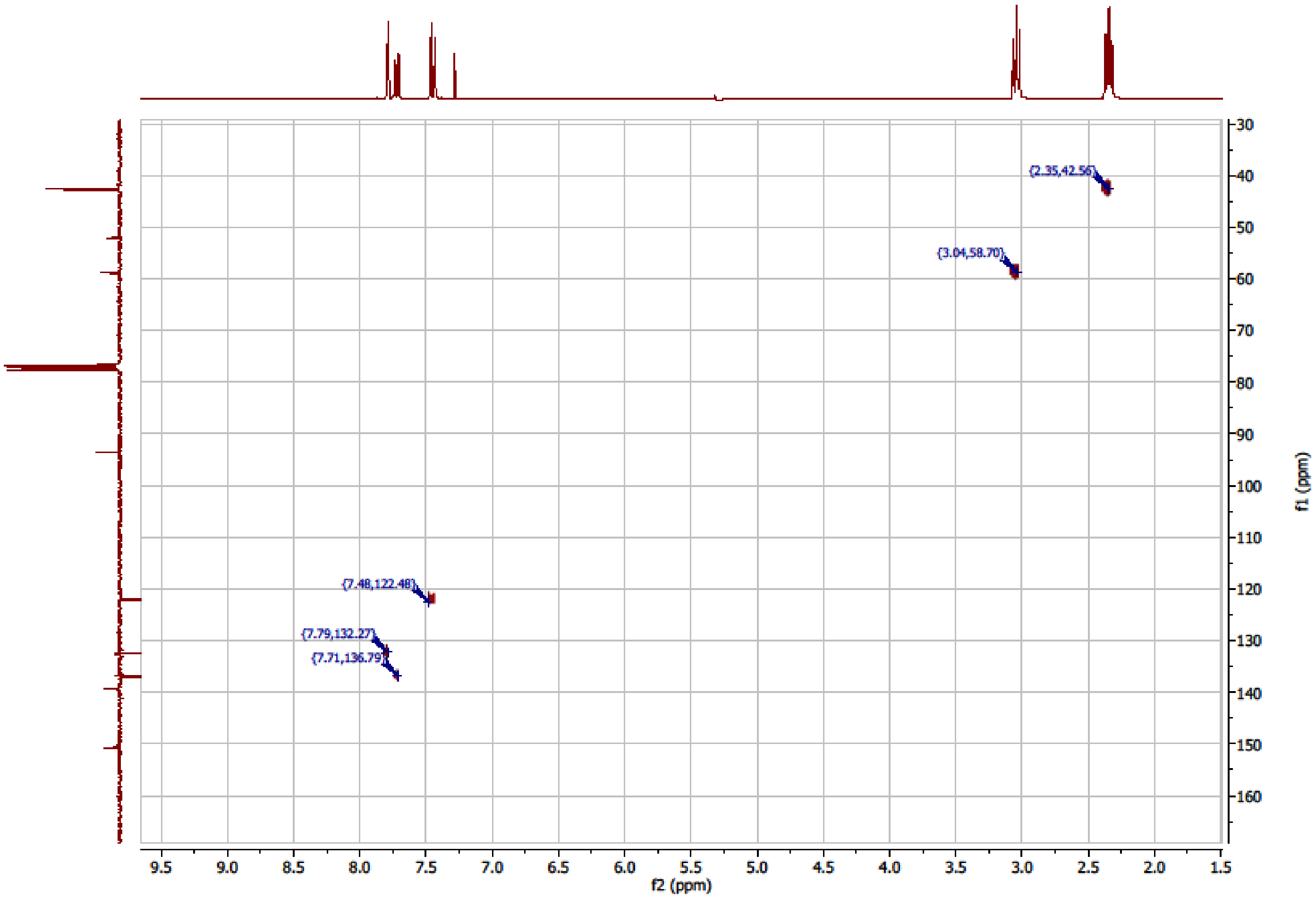

Figure S15. HSQC NMR of intermediate **8**.

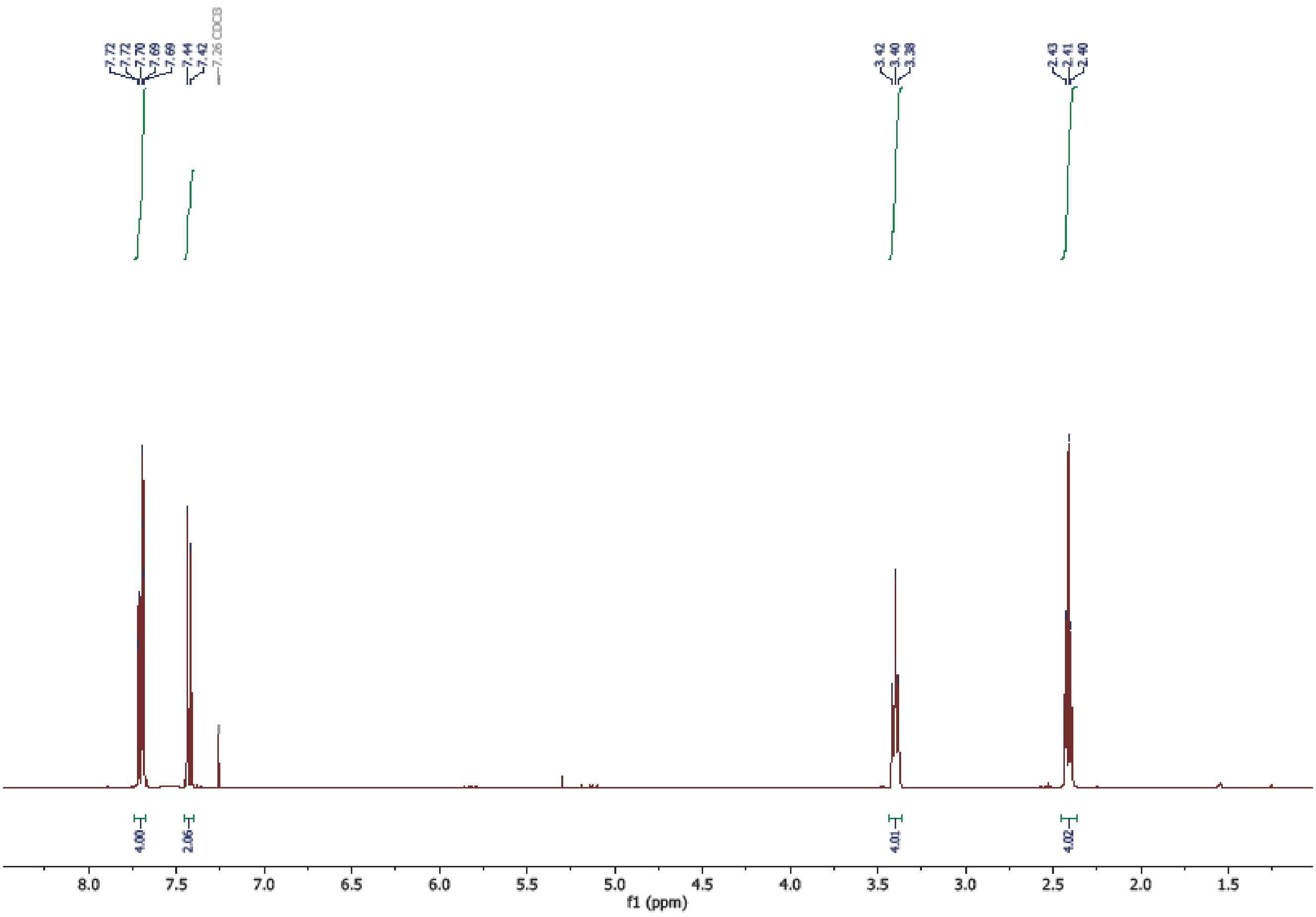

Figure S16. ^{1}H NMR of intermediate **4**.

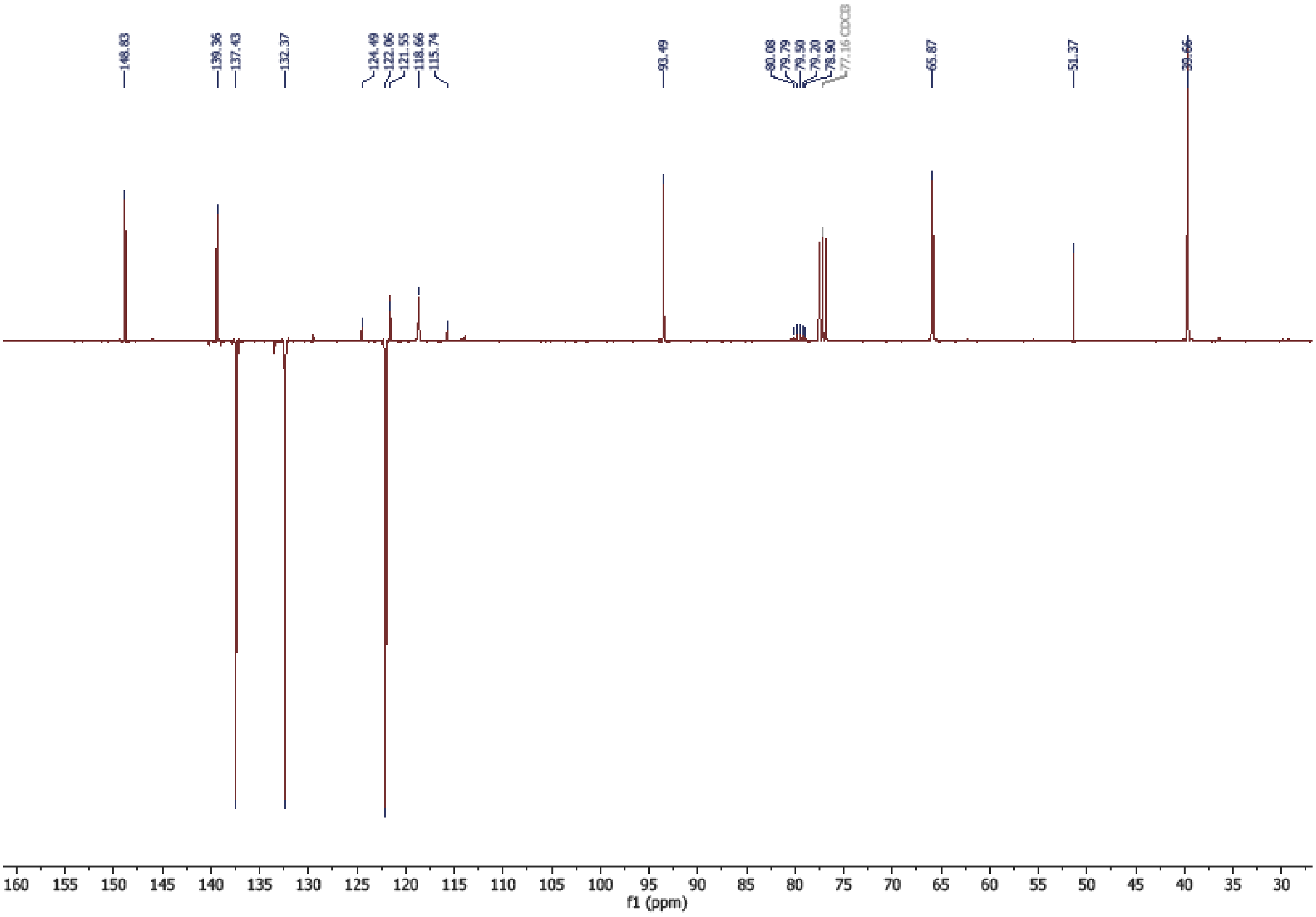

Figure S17. ^{13}C APT NMR of intermediate **4**.

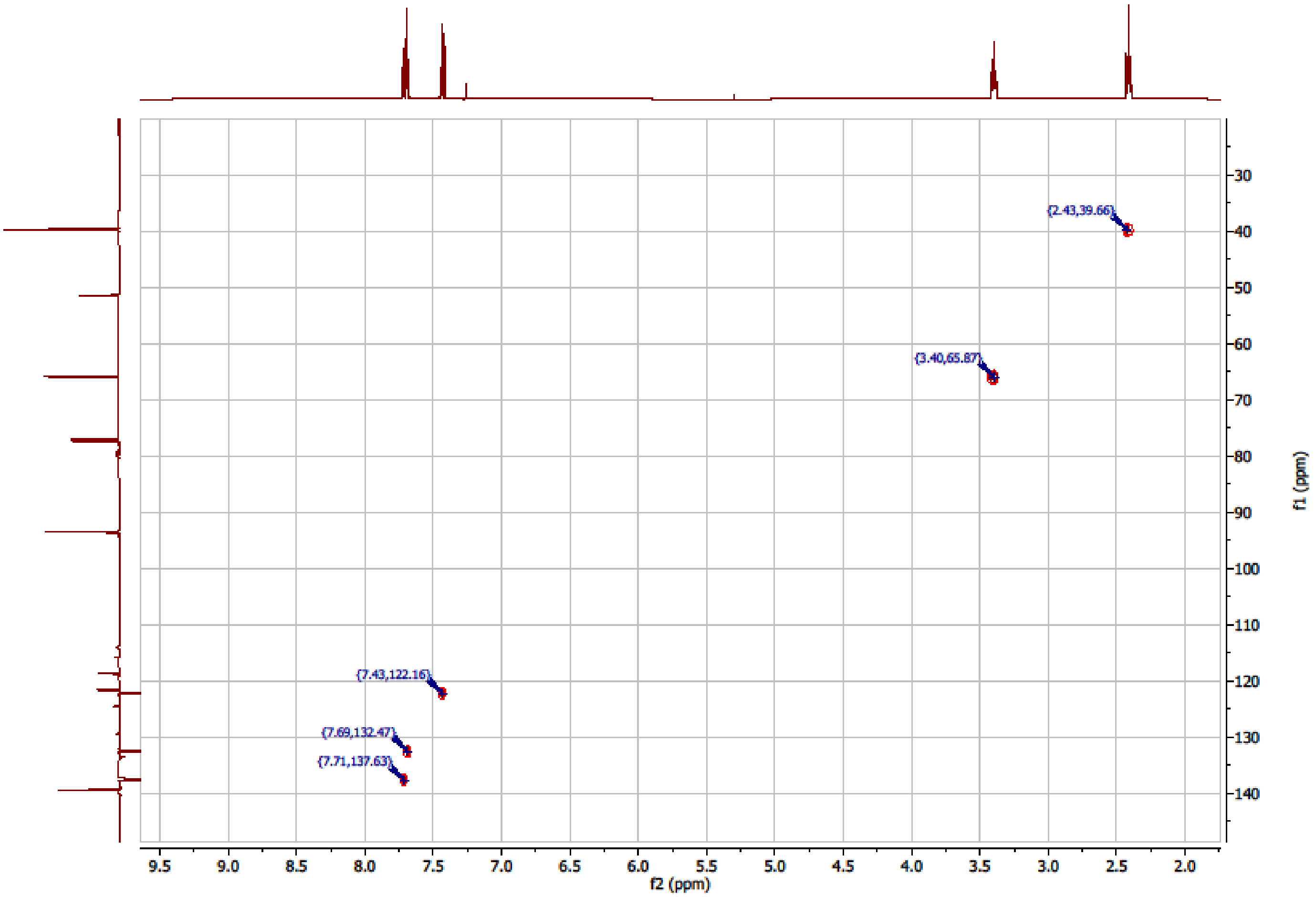

Figure S18. HSQC NMR of intermediate **4**.

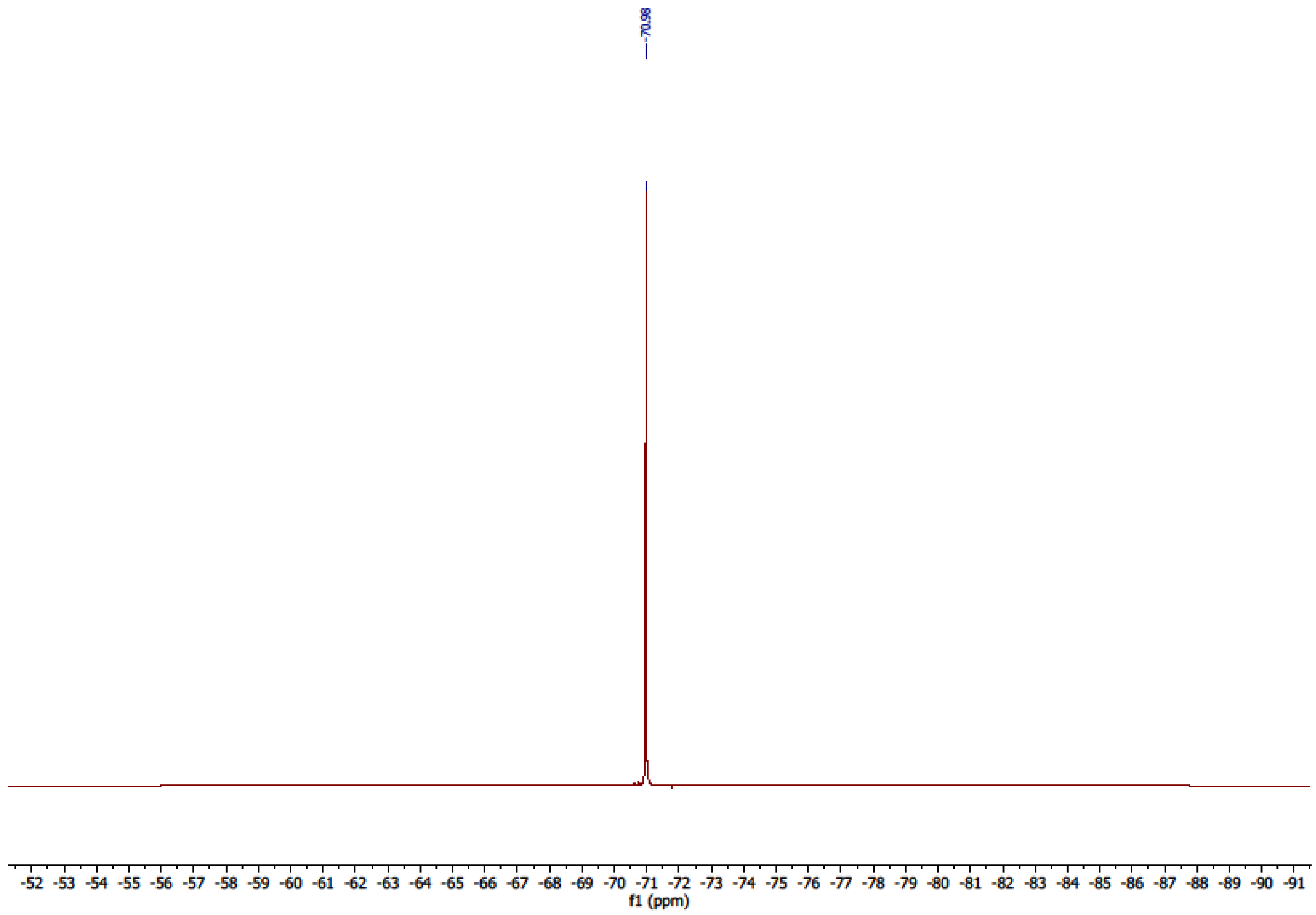

Figure S19. ^{19}F NMR of intermediate **4**.

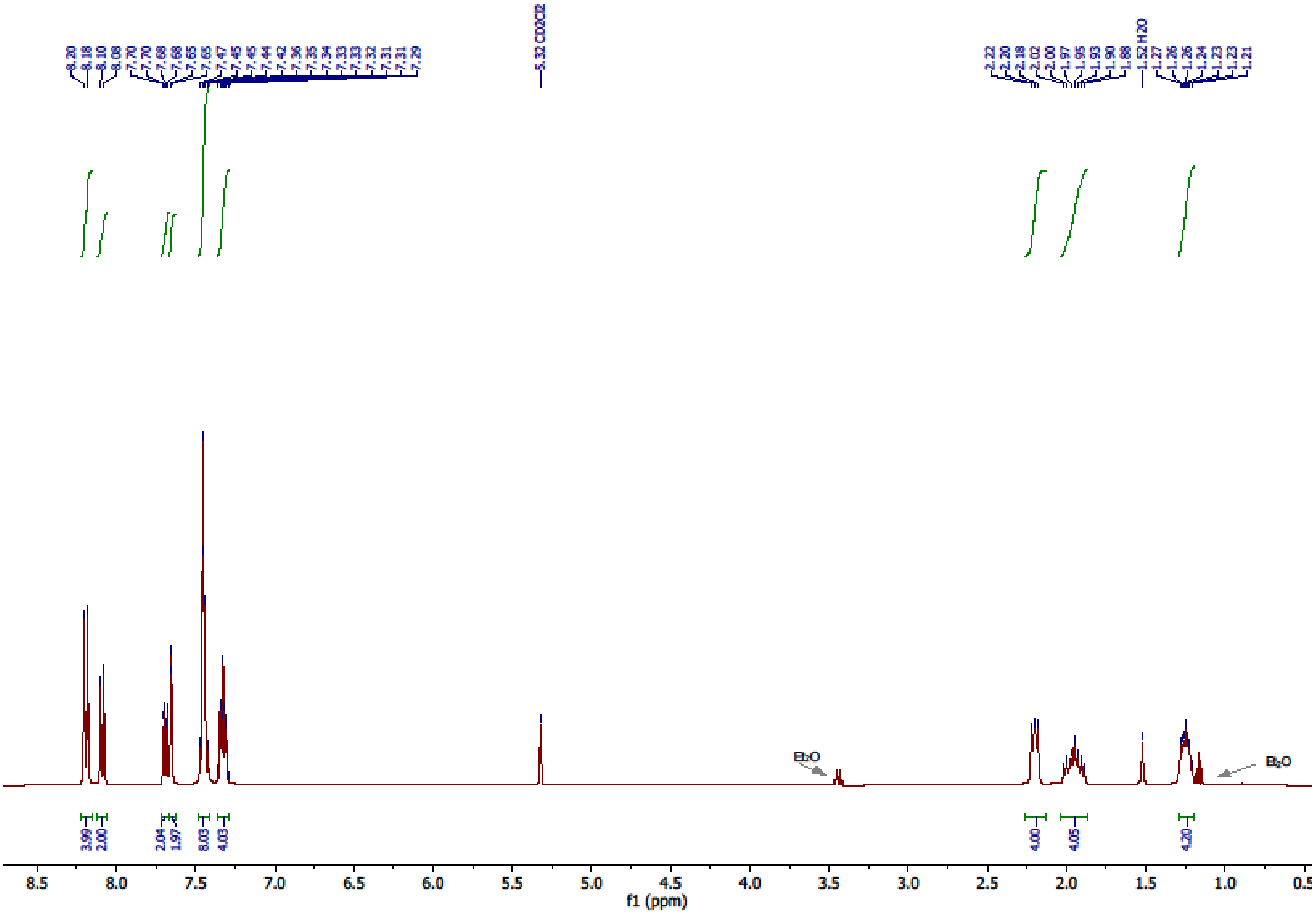

Figure S20. ^{1}H NMR of **CFC-F2**.

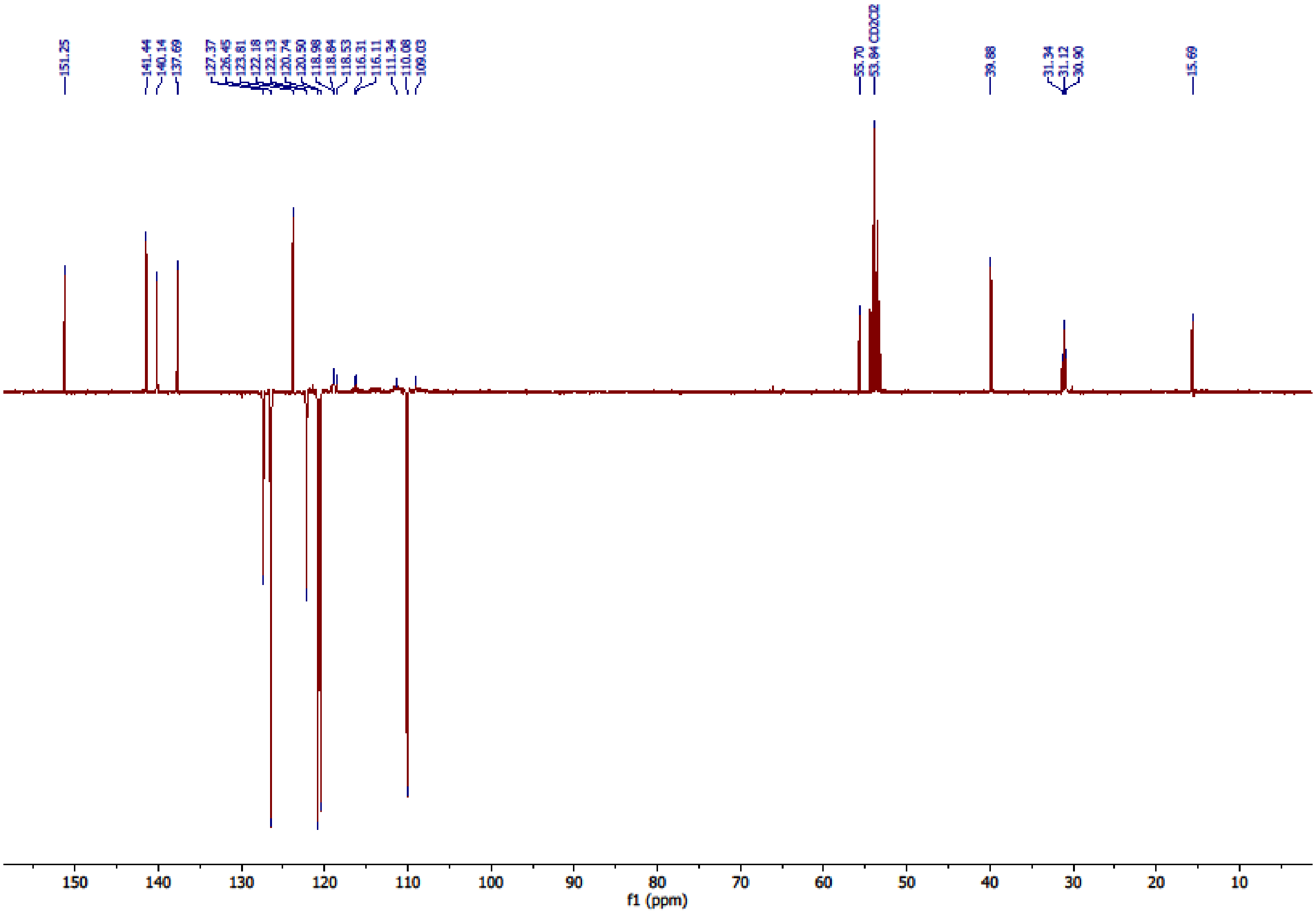

Figure S21. ^{13}C APT NMR of **CFC-F2**.

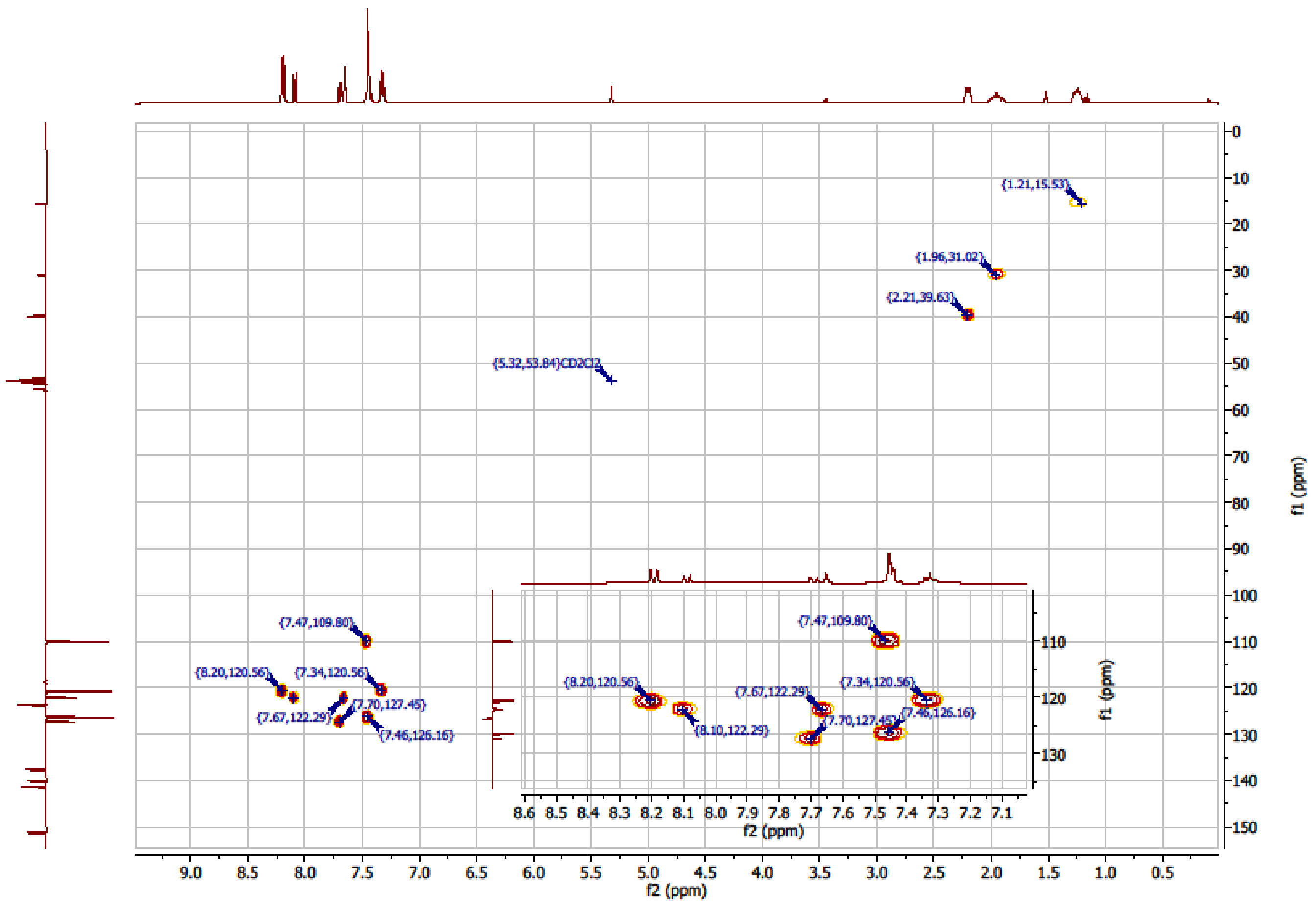

Figure S22. HSQC NMR of **CFC-F2**.

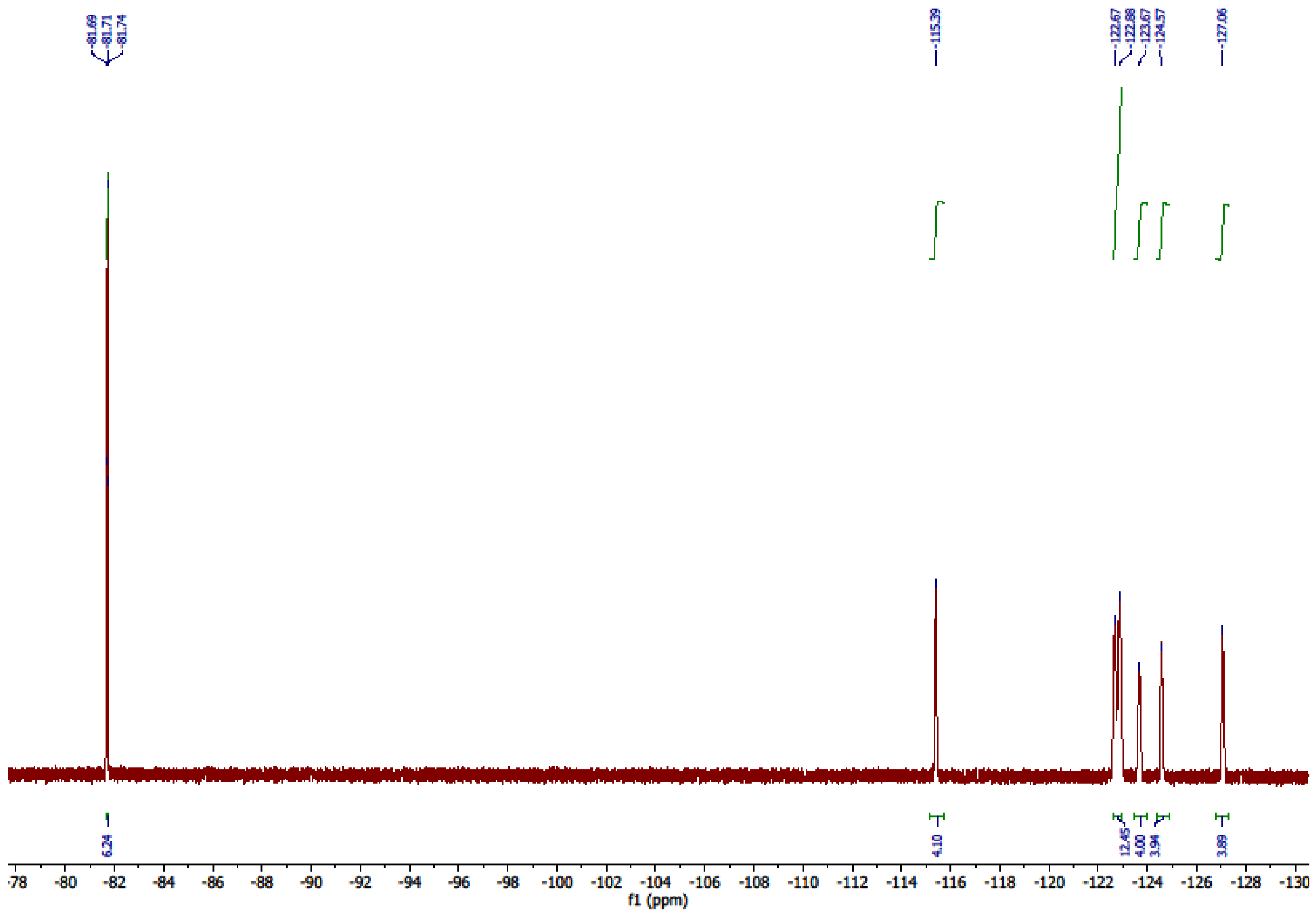

Figure S23. ^{19}F NMR of **CFC-F2**.

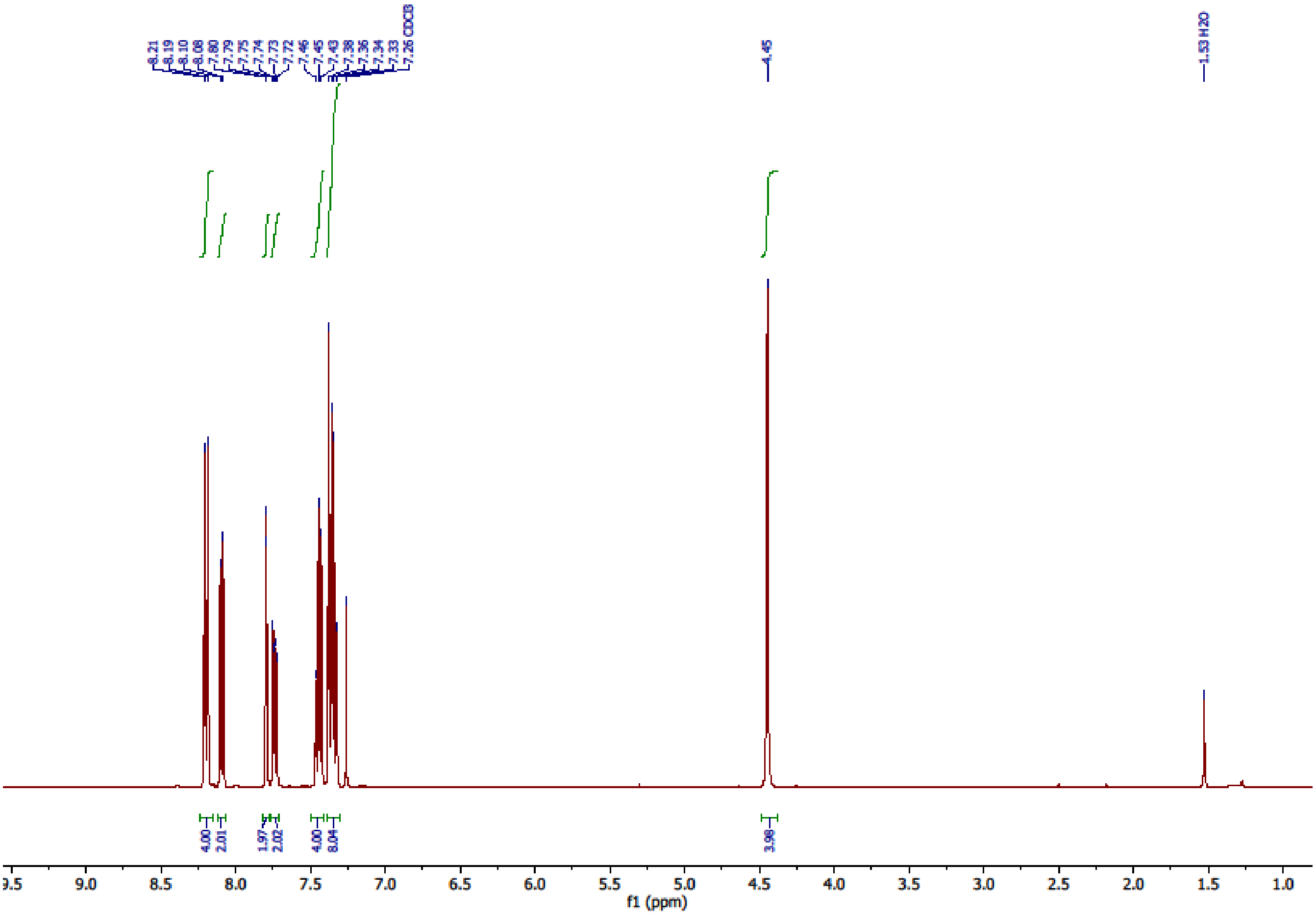

Figure S24. ^{1}H NMR of **CFC-F3**.

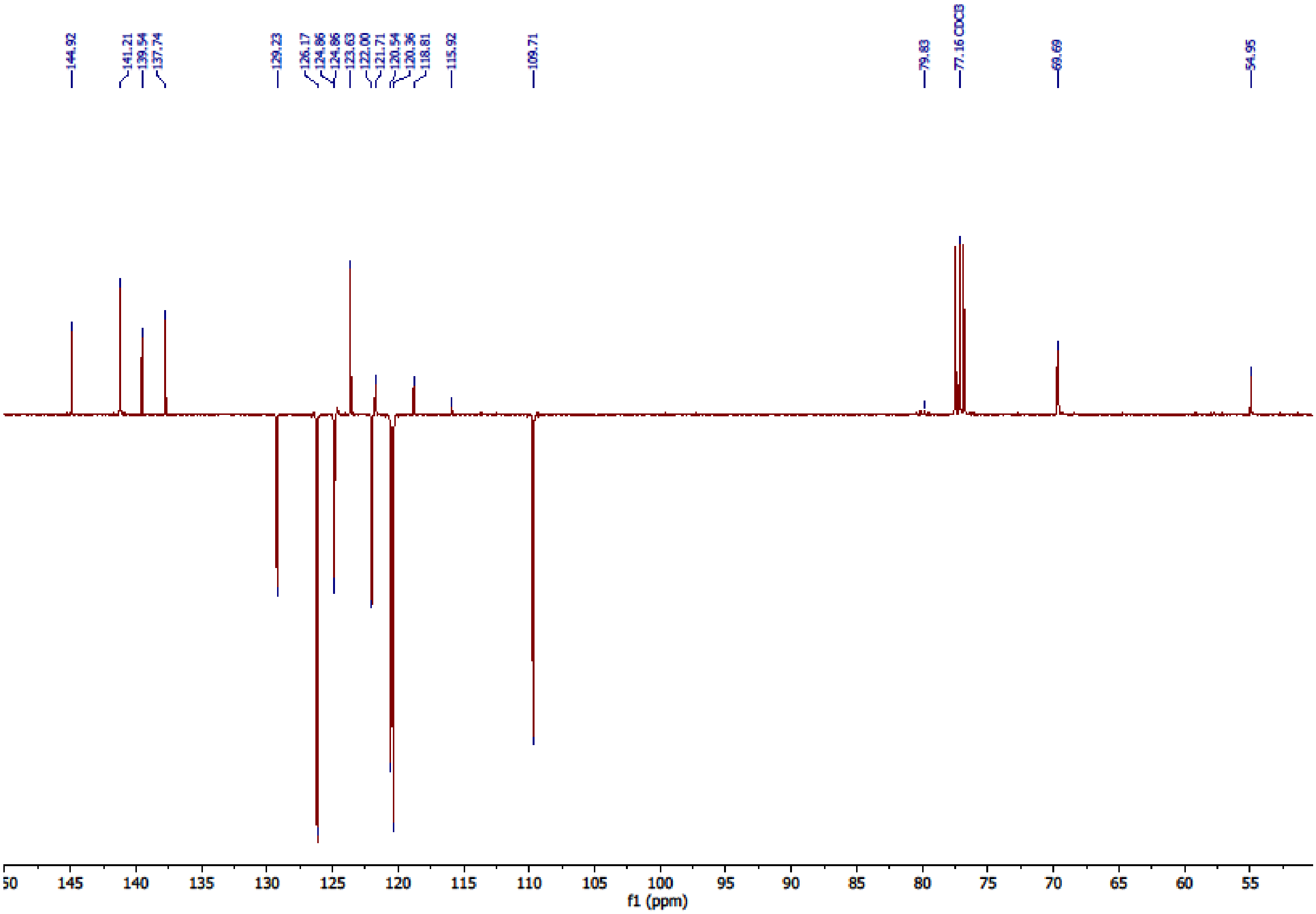

Figure S25. ^{13}C APT NMR of **CFC-F3**.

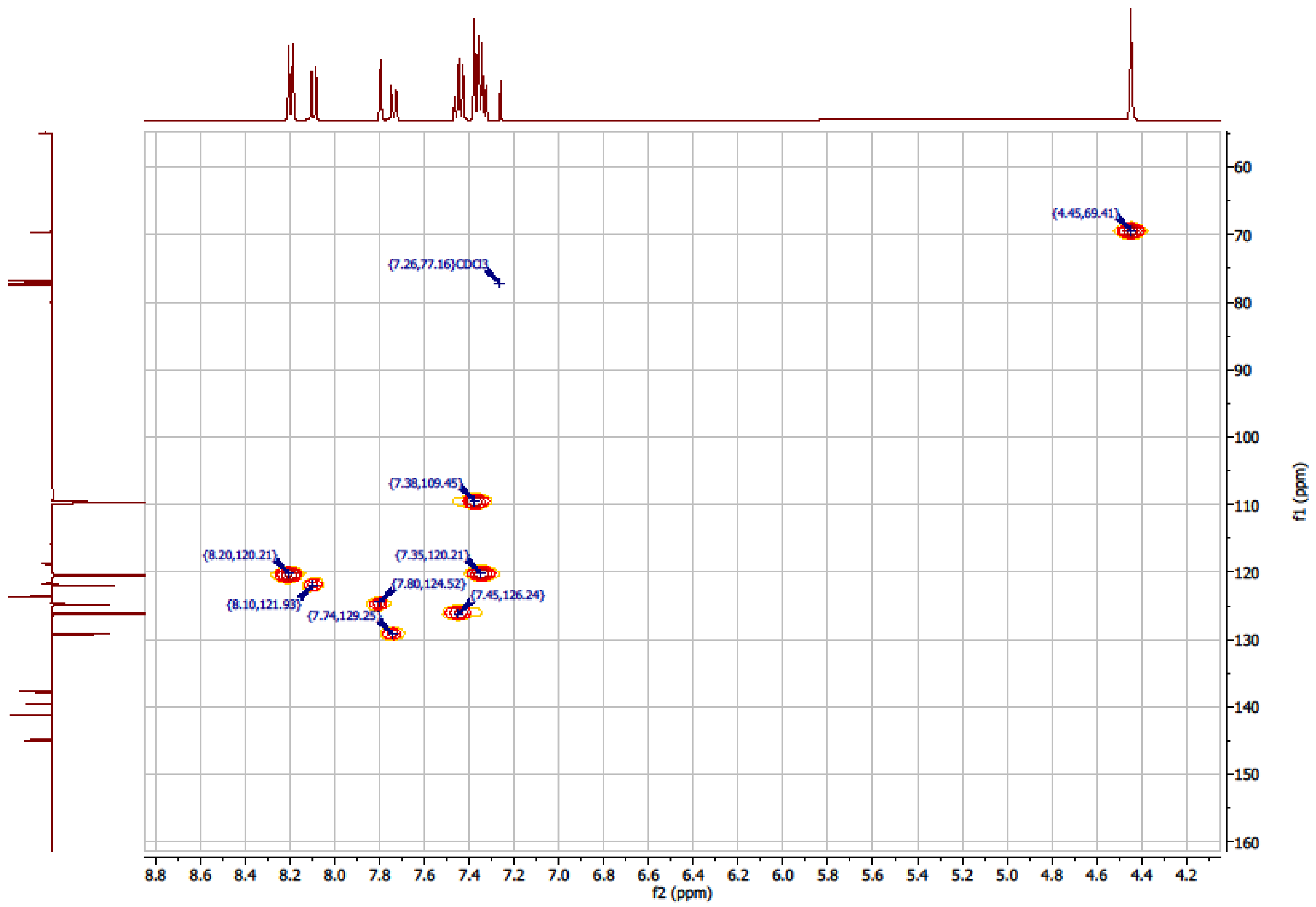

Figure S26. HSQC NMR of **CFC-F3**.

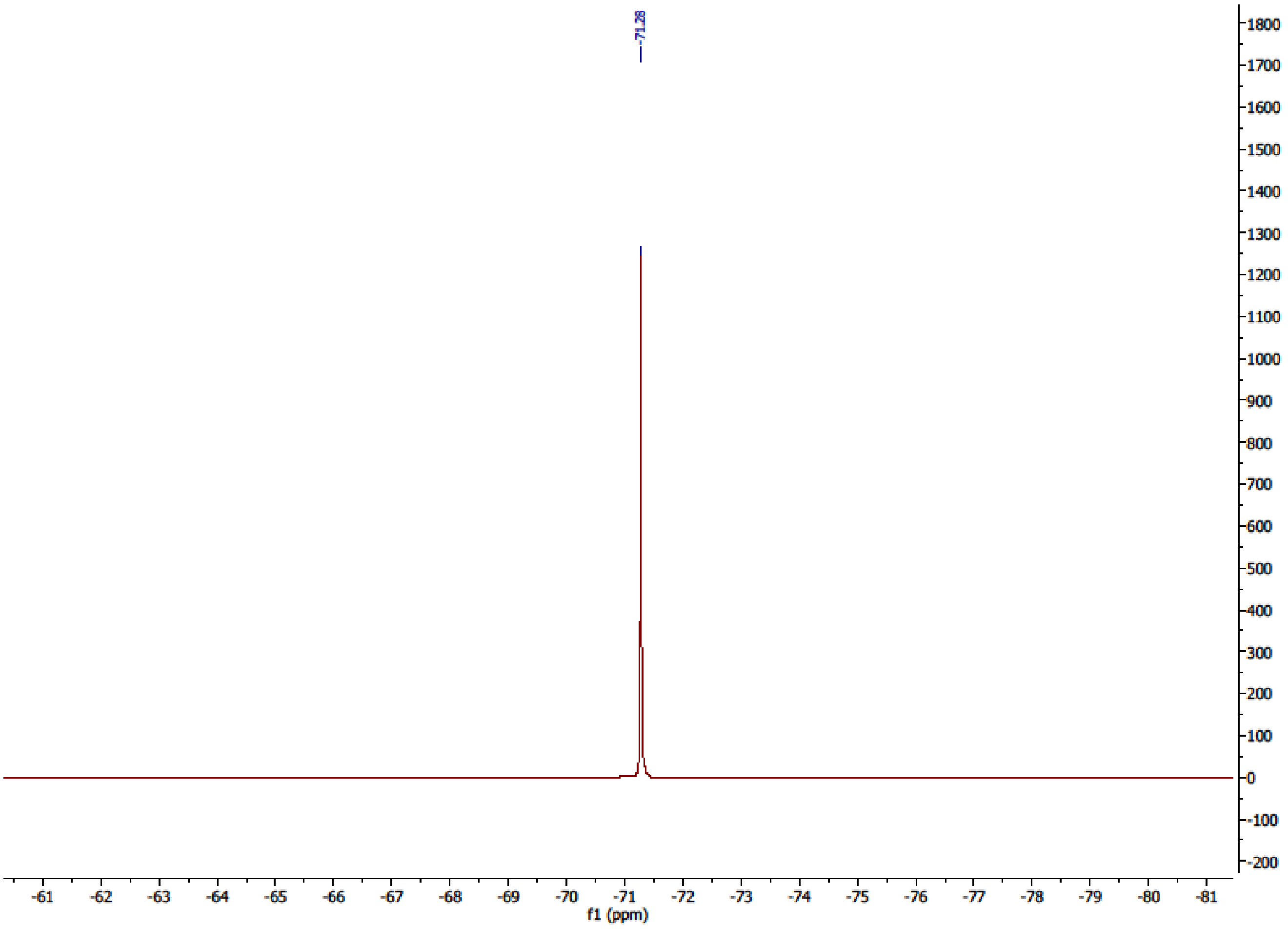

Figure S27. ^{19}F NMR of **CFC-F3**.

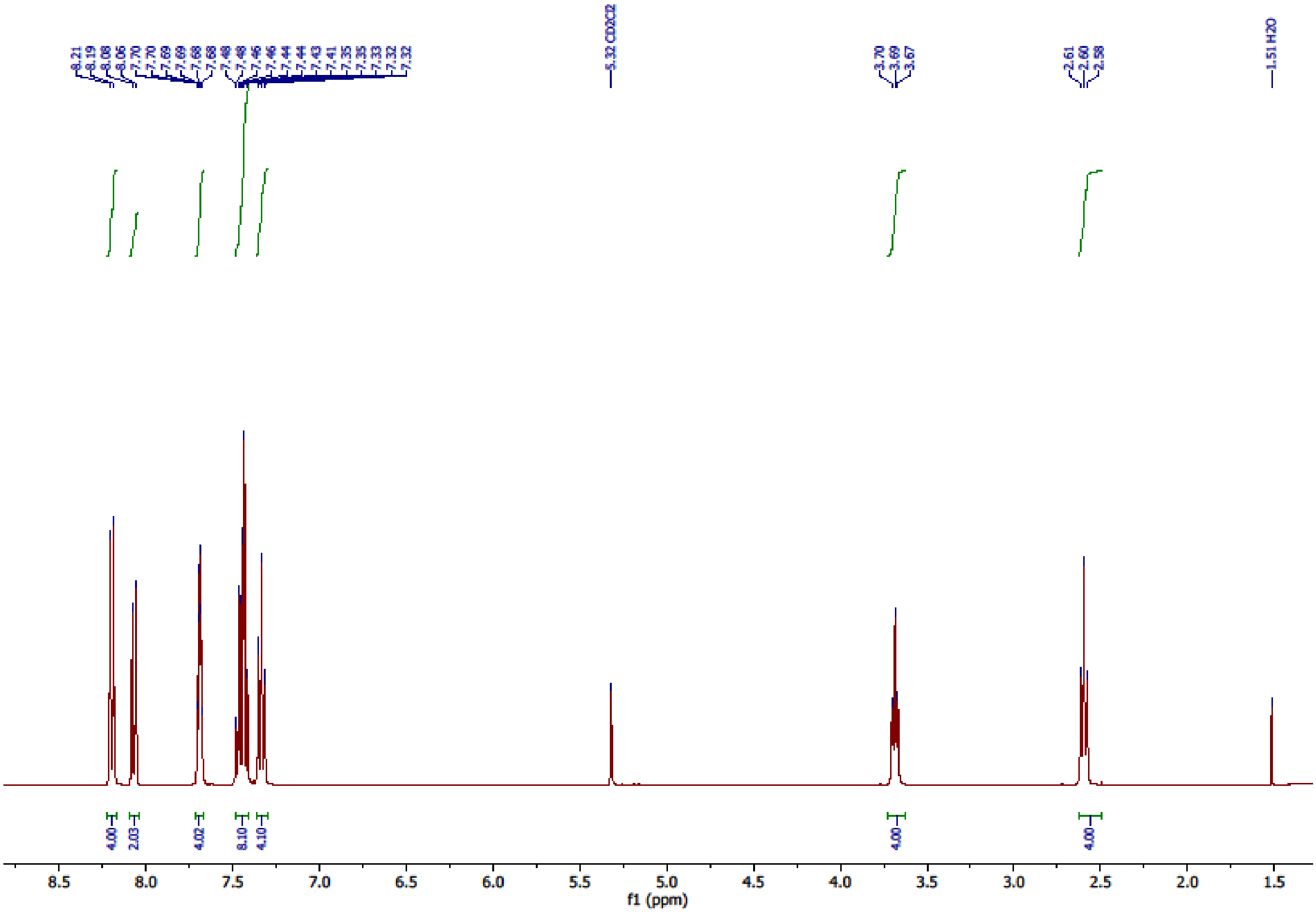

Figure S28. ^{1}H NMR of **CFC-F4**

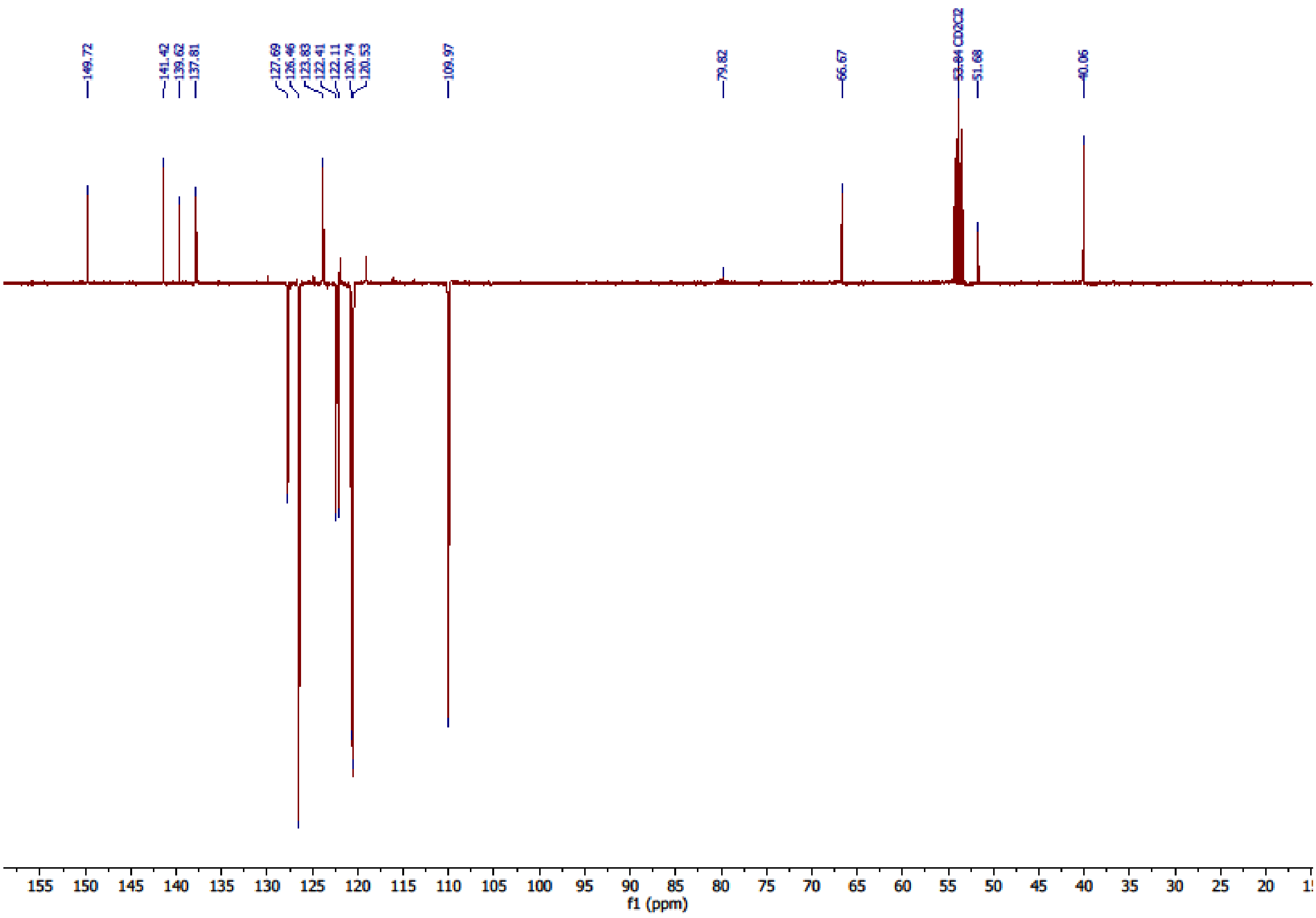

Figure S29. ^{13}C APT NMR of **CFC-F4**.

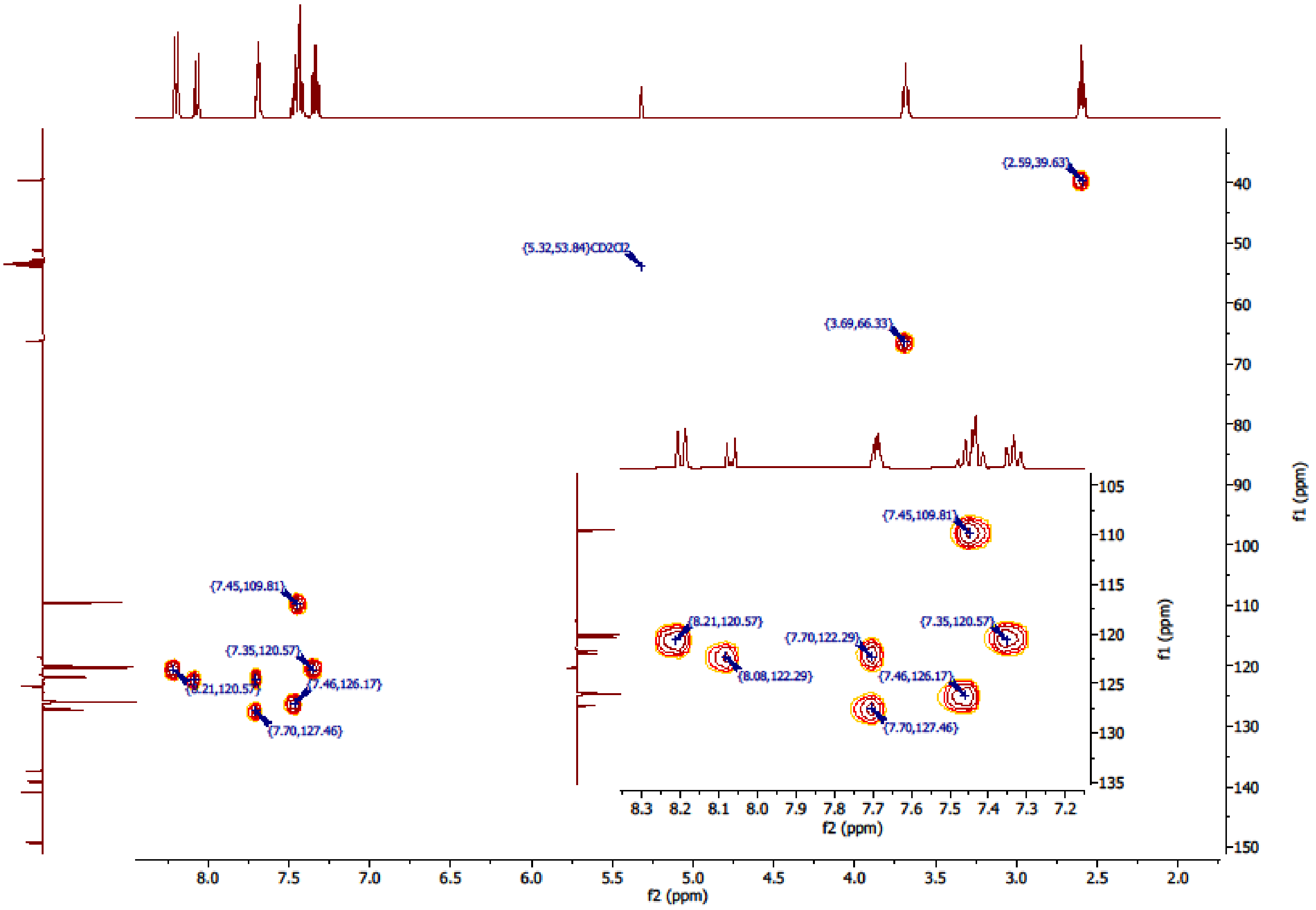

Figure S30. HSQC NMR of **CFC-F4**.

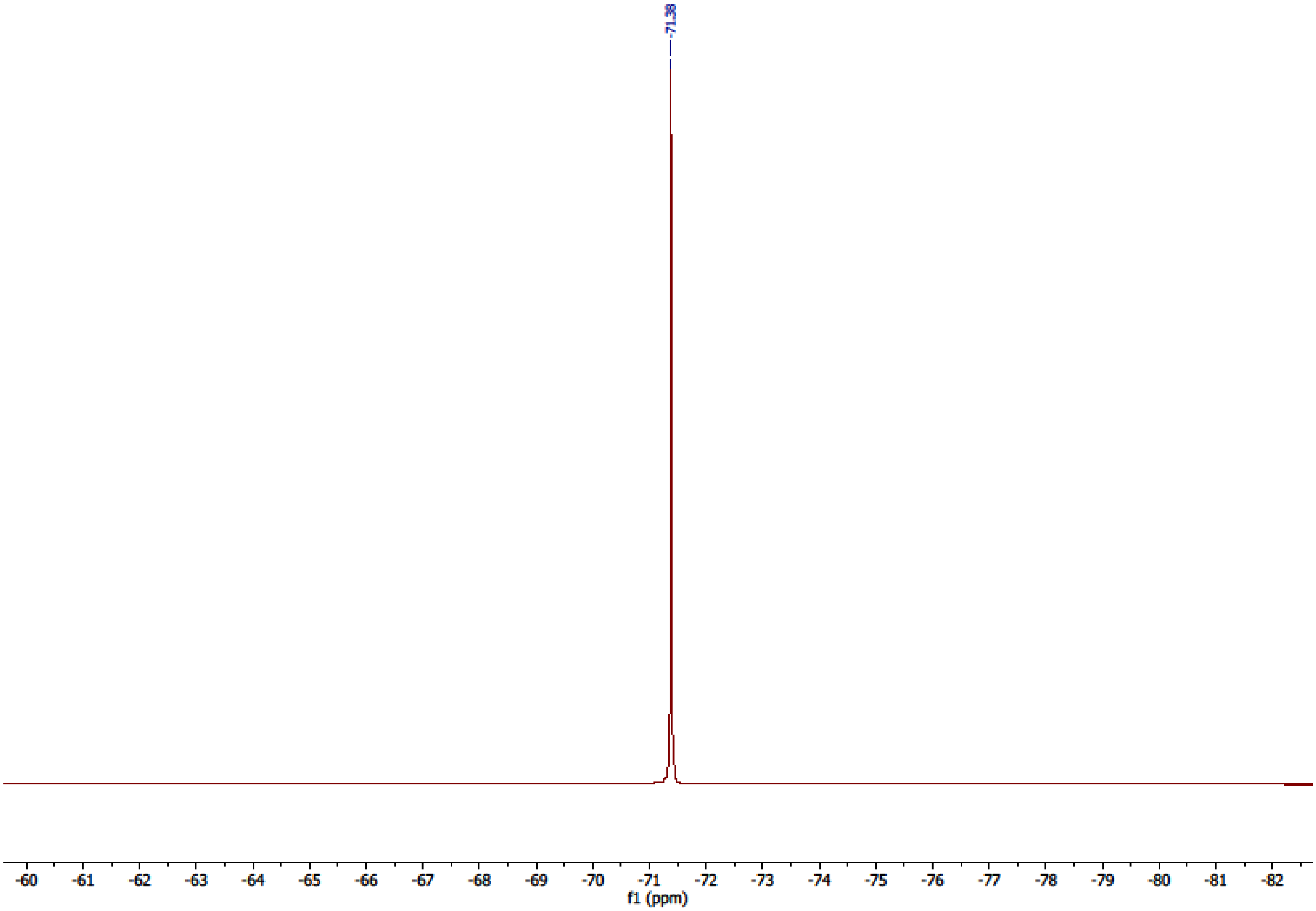

Figure S31. ^{19}F NMR of **CFC-F4**.

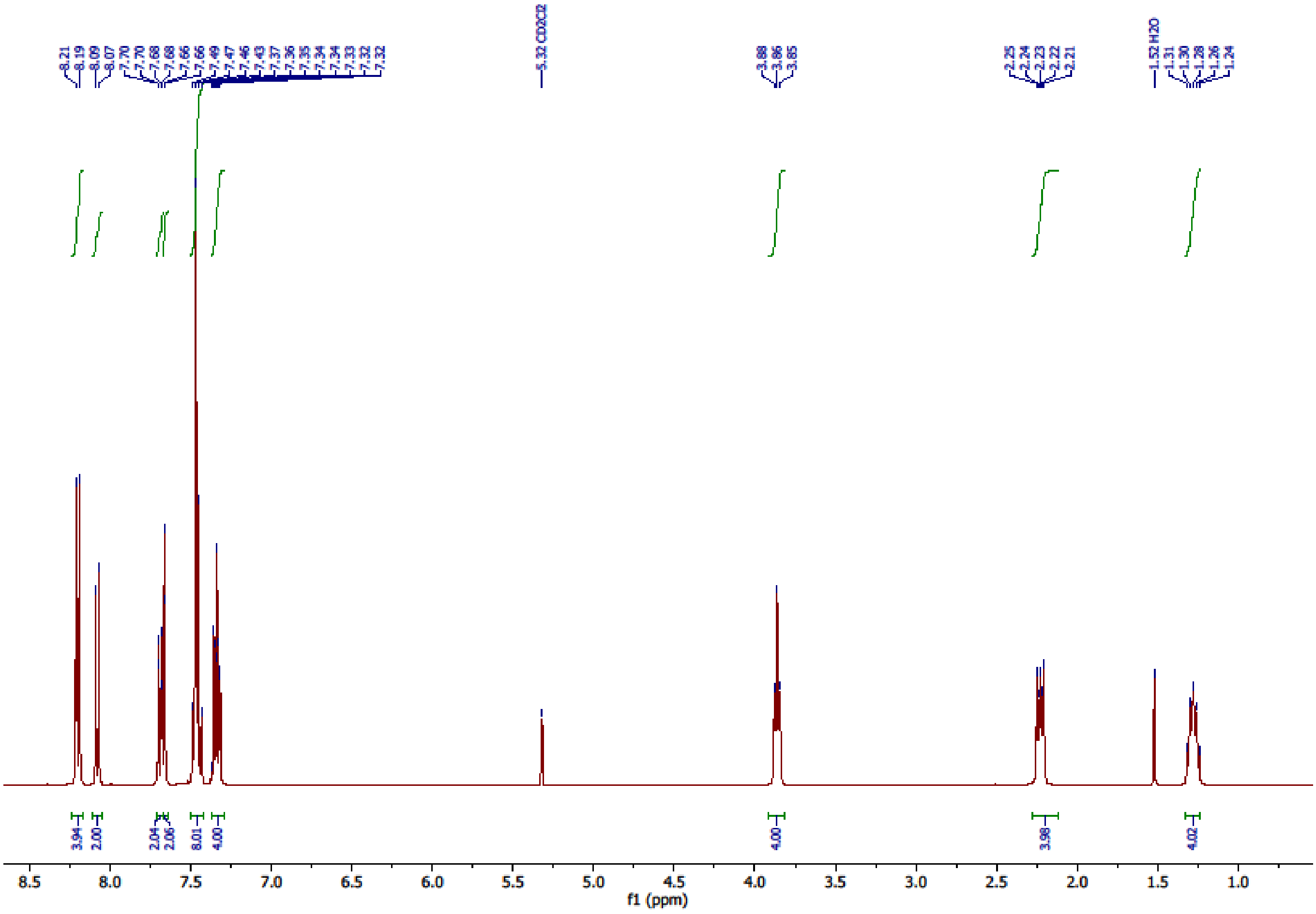

Figure S32. ^{1}H NMR of **CFC-F5**.

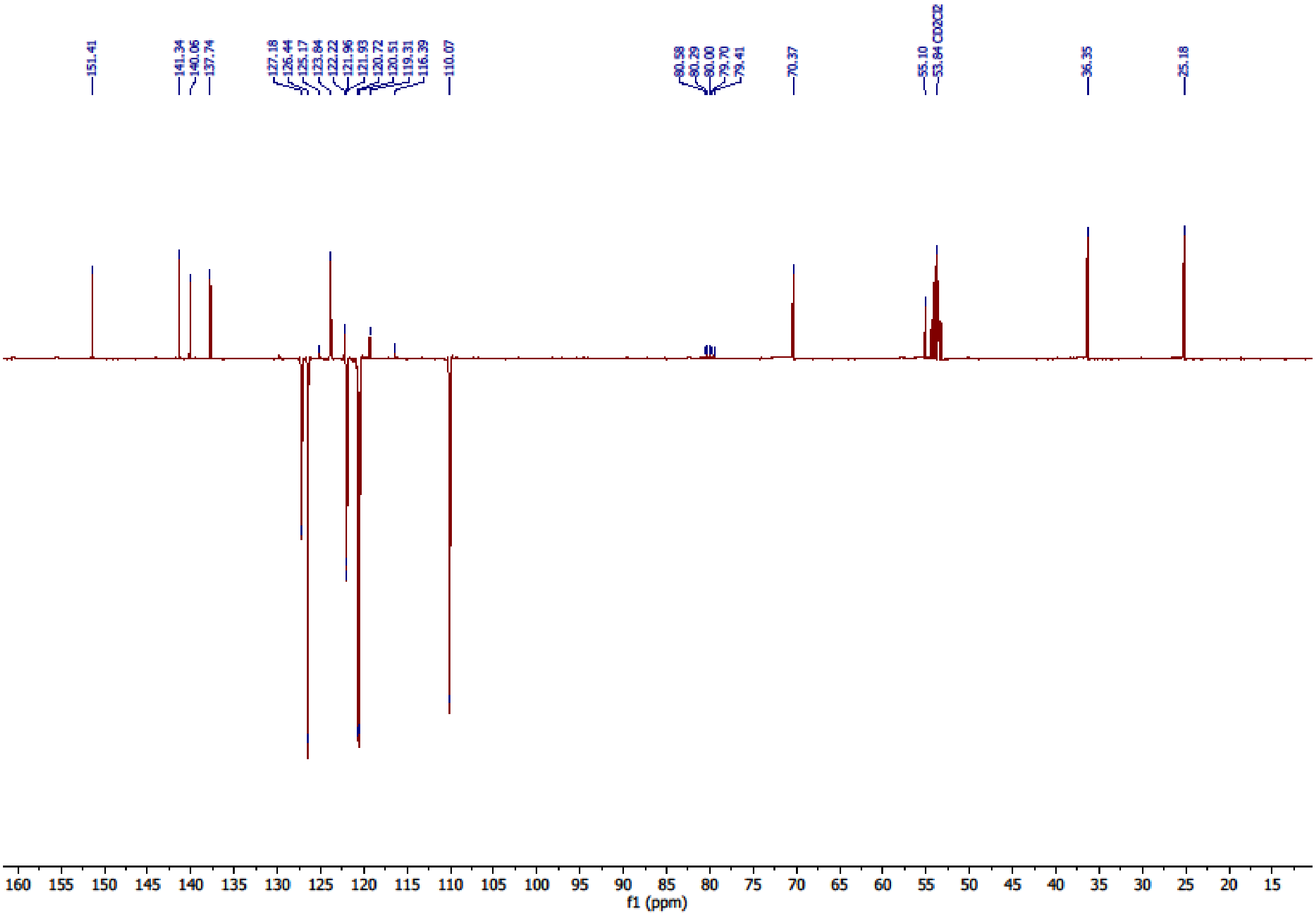

Figure S33. ^{13}C APT NMR of **CFC-F5**.

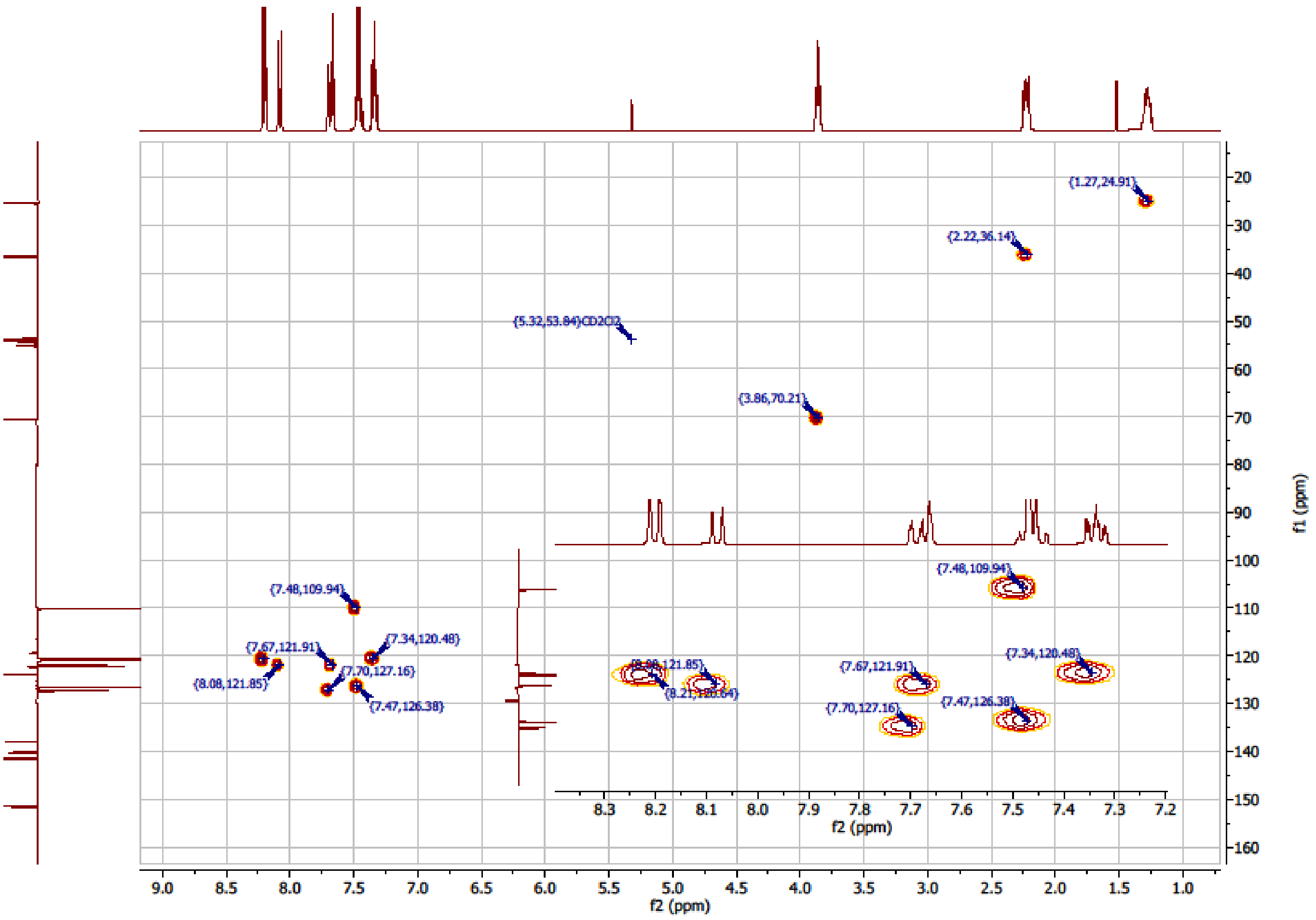

Figure S34. HSQC NMR of CFC-F5.

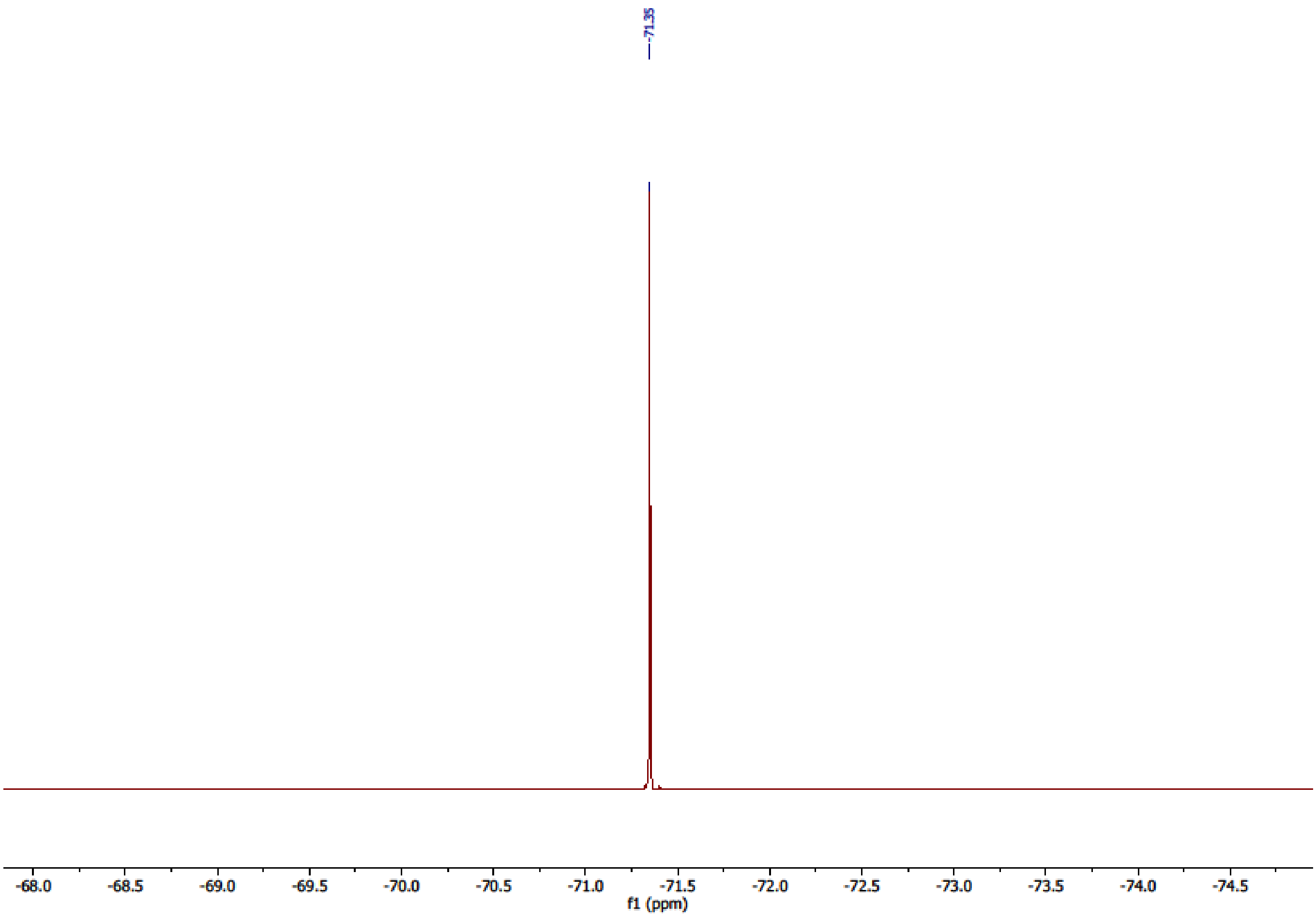

Figure S35. ^{19}F NMR of **CFC-F5**.

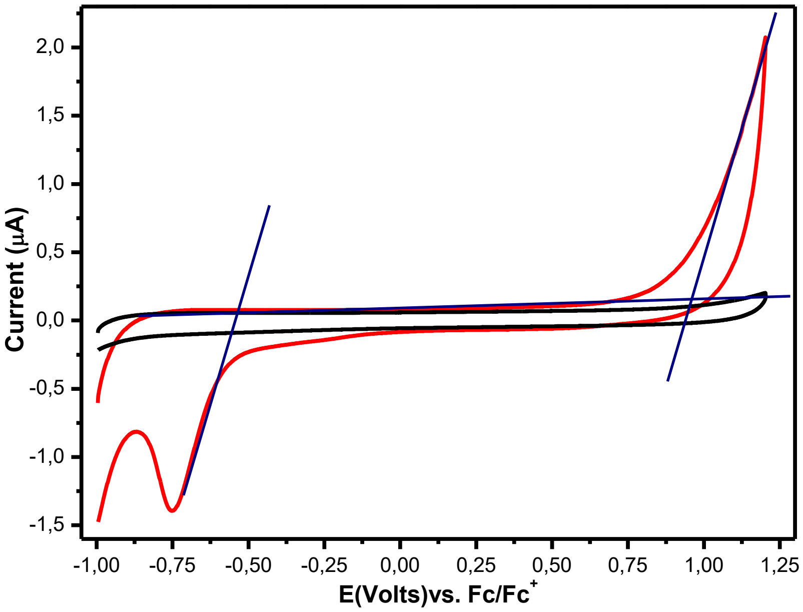

Figure S36. Cyclic voltammogram of electrolyte only (black line) and with 1000 ppm of H_2O (red line). Conditions: Pt working electrode, electrolyte $TBAPF_6$ 0.1 M solution in DCE, Scan rate: 100 mV/s.

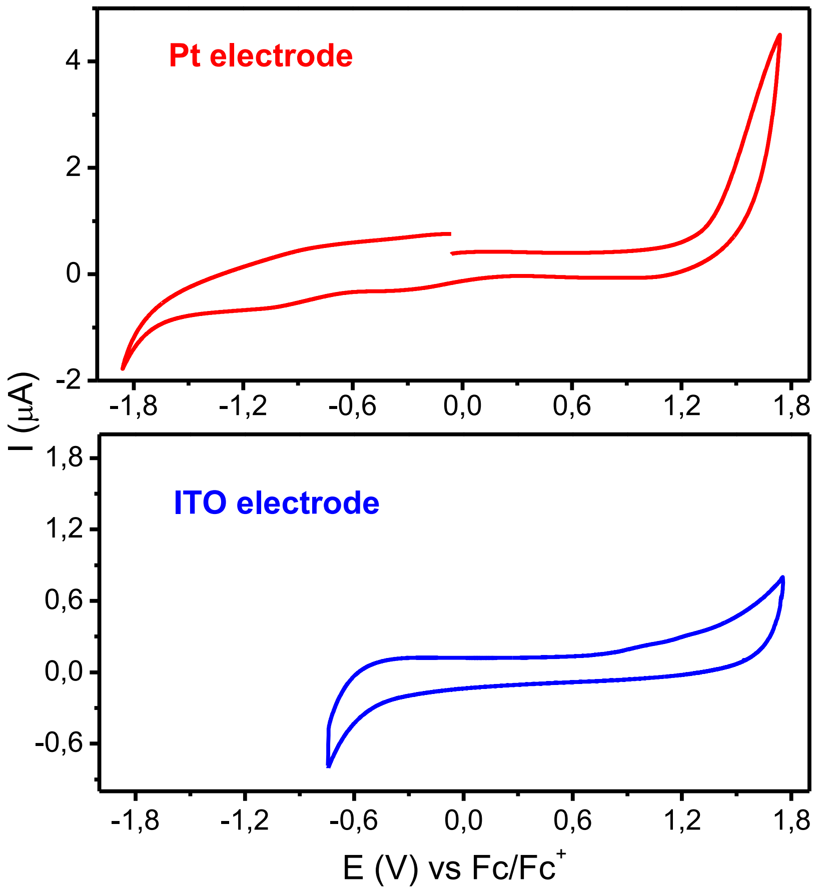

Figure S37. Blank cyclic voltammogram curves for the three-electrode systems with the addition of the supporting electrolyte

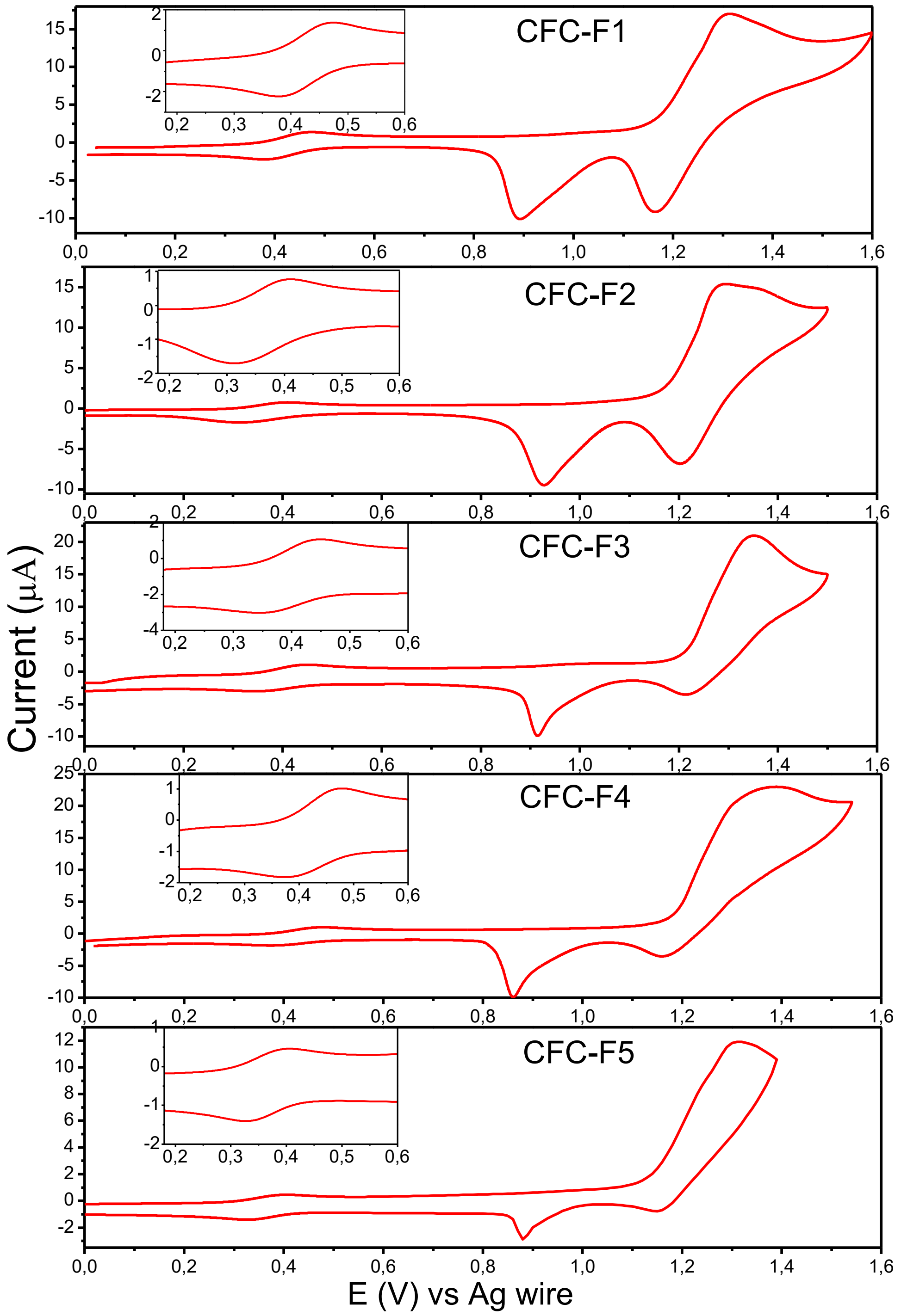

Figure S38. Cyclic voltammogram curves (insets = Fc/Fc+ reference system).

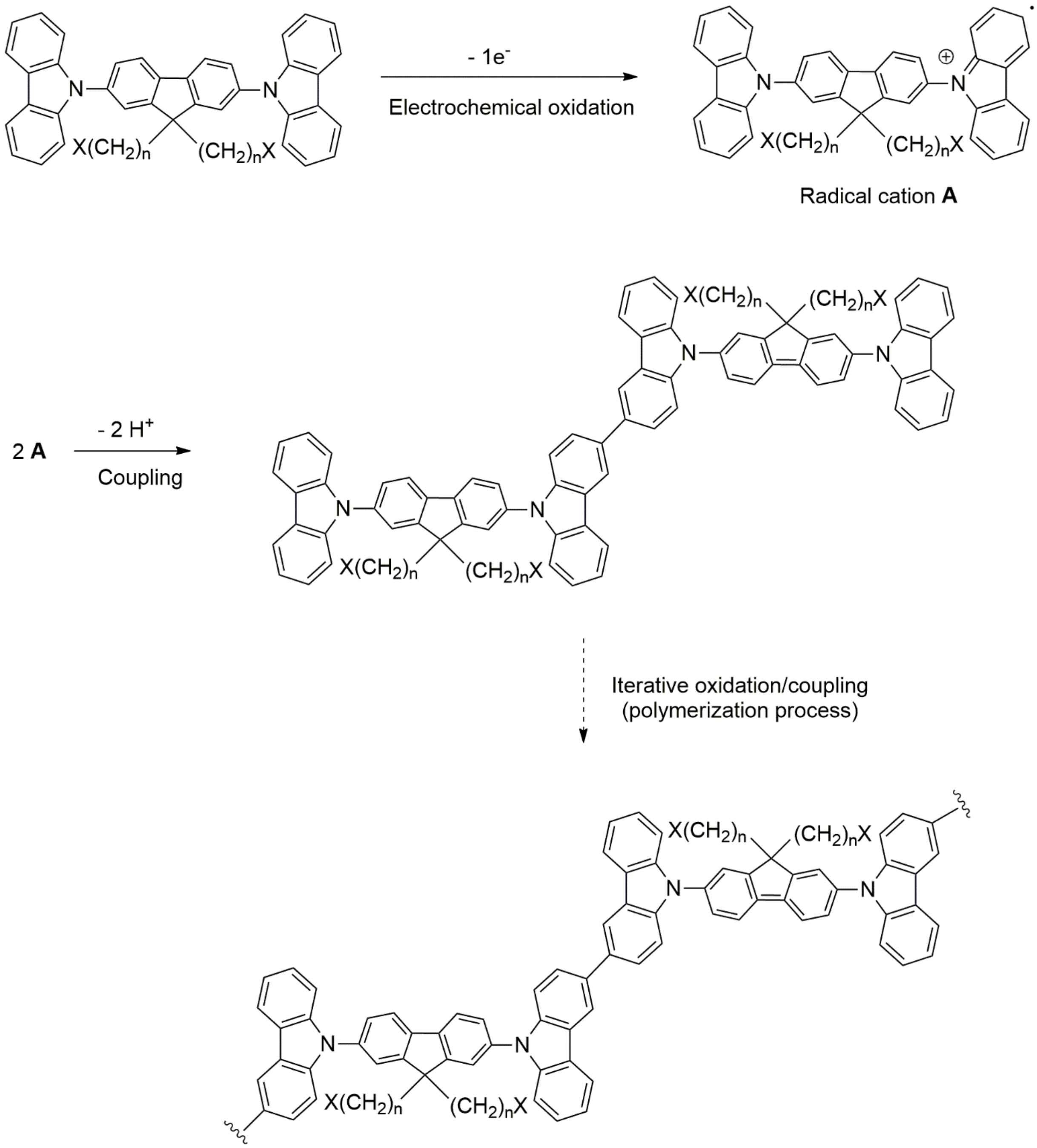

Figure S39. Pictorial view of the electropolymerization steps of **CFC-Fn** derivatives

potential range(V)	CFC-F1		CFC-F2	
	0 ppm H_2O	500 ppm H_2O	0 ppm H_2O	500 ppm H_2O
-0,3 to 1,1	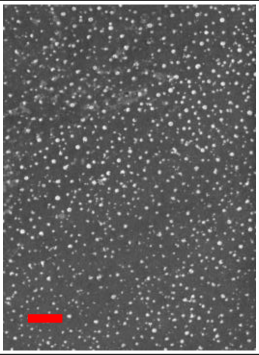	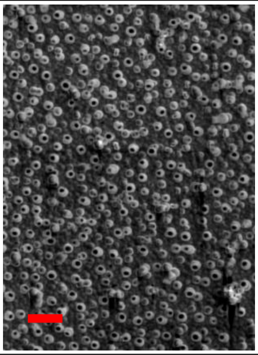	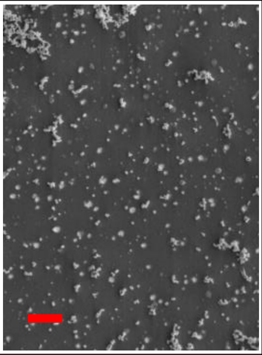	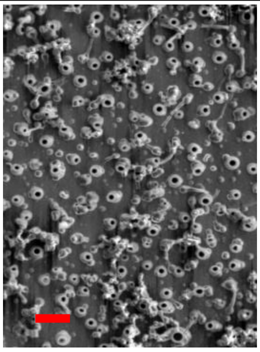
-0,3 to 0,85	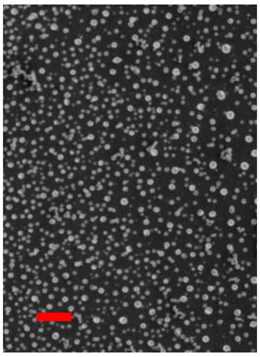	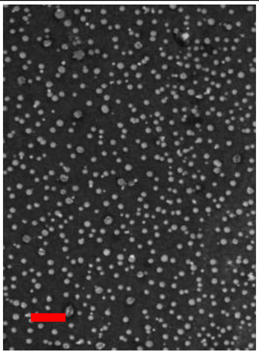	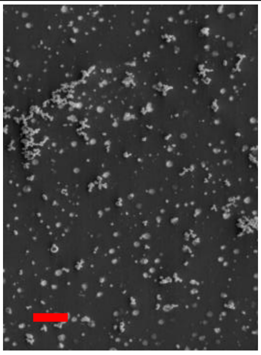	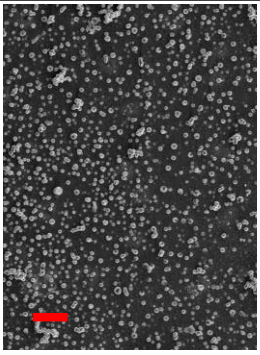

Figure S40. SEM images of the CFC-F1 and CFC-F2 surface on the ITO electrode without and 500 ppm of H_2O electrodeposited by cyclic voltammetry at different potential range. The monomers concentration was fixed at 5 mM in the DCE solution containing 0.1 M $TBAPF_6$ as the electrolyte. Scan rate: 20 mV/s. Scale bar: 4 µm.

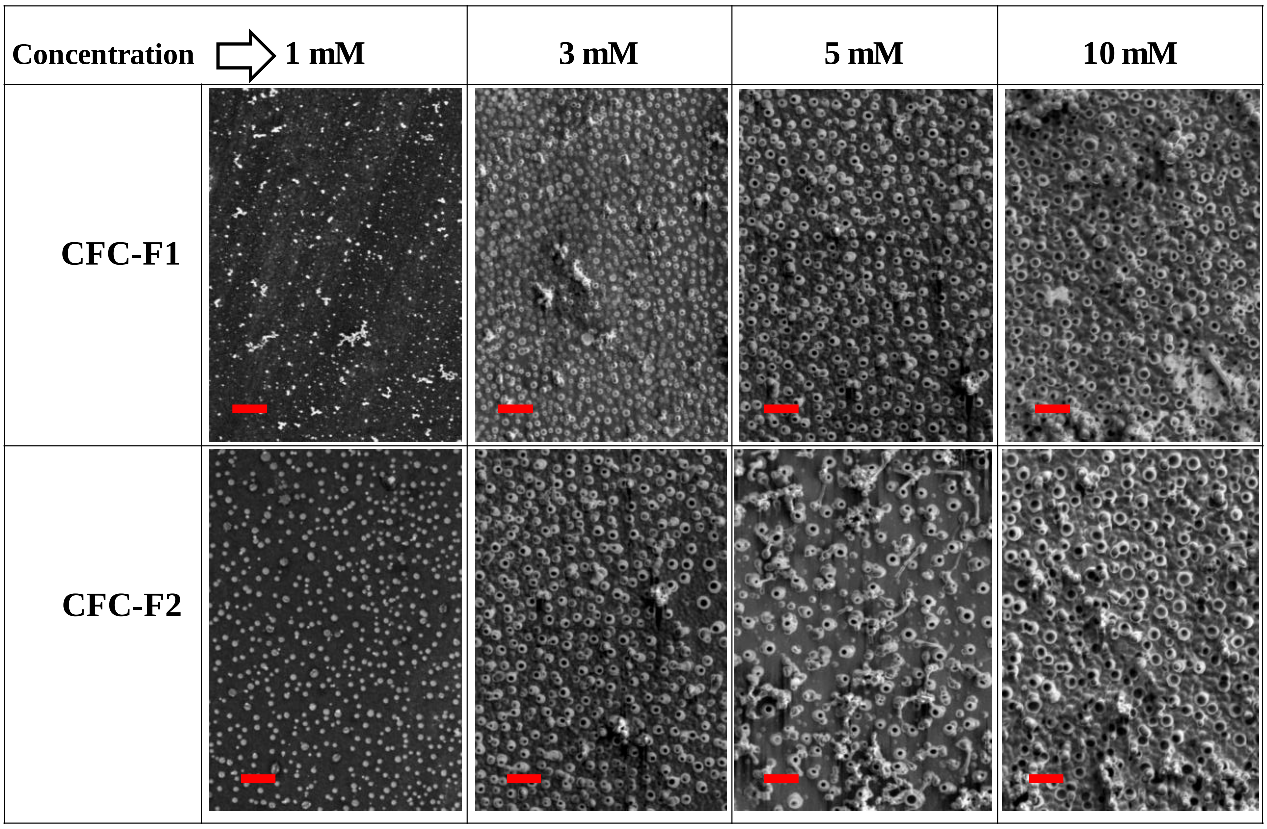

Figure S41. SEM images of the CFC-F1 and CFC-F2 surface on the ITO electrode at different monomers concentration wich 500 ppm of H_2O electrodeposited by cyclic voltammetry in the DCE solution containing 0.1 M $TBAPF_6$ as the electrolyte. Scan rate: 20 mV/s. Scale bar: 4 µm

Monomers and experimental conditions	showed in paper	second test: unfiltered	second test: filtered
**CFC-F1 3 mM and 500 ppm of water at 20 mV/s**	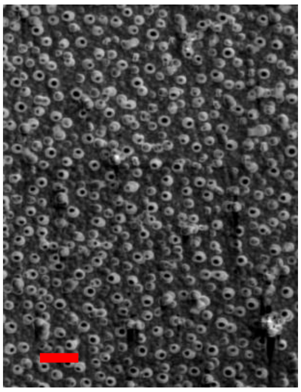	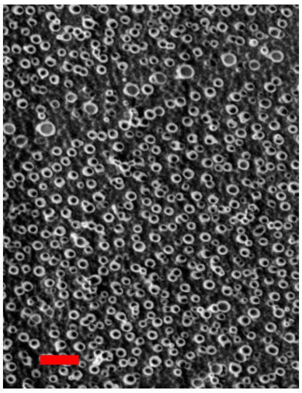	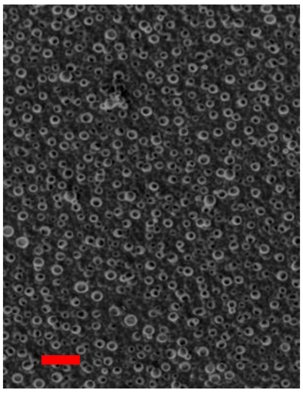
**CFC-F2 3 mM and 500 ppm of water at 5 mV/s**	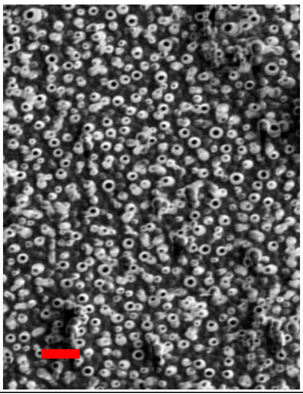	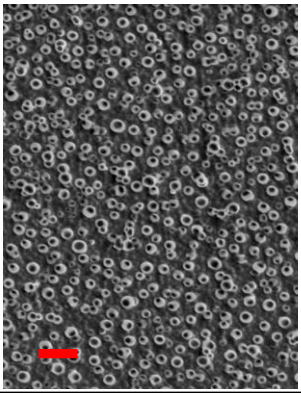	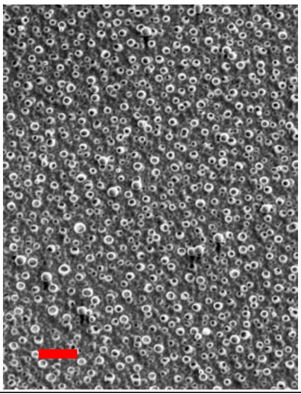

Figure S42. SEM images of the CFC-F1 and CFC-F2 surface on the ITO electrode at different experimental conditions. The second test is the same solution unfiltered and filtered. Scale bar: 4 µm
